# Synthesis of 4-*O*-Alkylated *N*-Acetylneuraminic Acid Derivatives

**DOI:** 10.1021/acs.joc.1c00235

**Published:** 2021-06-17

**Authors:** Emil Johansson, Rémi Caraballo, Mikael Elofsson

**Affiliations:** Department of Chemistry, Umeå University, Umeå SE90187, Sweden

## Abstract

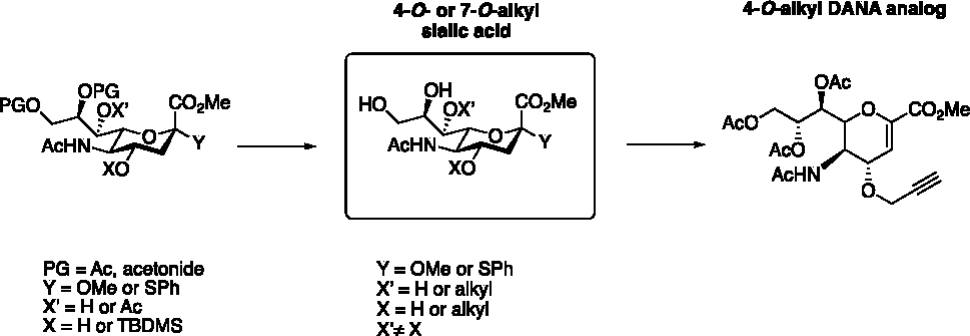

The synthesis of 4-*O*-alkyl analogues of *N*-acetylneuraminic acid (Neu5Ac) and the scope of the reaction are described. Activated alkyl halides and sulfonates and primary alkyl iodides give products in useful yields. The utility of the methodology is exemplified using a thiophenyl Neu5Ac building block to synthesize a 4-*O*-alkyl DANA analogue. These results expand the toolbox of Neu5Ac chemistry with value in drug discovery and for the design of novel tools to study the biology of Neu5Ac lectins.

*N*-Acetylneuraminic acid (Neu5Ac, **1**, Figure 1) is typically found at the terminal end of glycolipids and glycoproteins that decorate the surfaces of all mammalian cell types. Neu5Ac is involved in mediating or modulating a variety of physiological and pathophysiological processes.^1^ One of the most well-known roles of Neu5Ac is in the replication cycle of the influenza virus.^2^ Accordingly, substantial efforts have been placed on the development of Neu5Ac-based antivirals,^3^ where modifications of the C4-position of 2-deoxy-2,3-didehydro-*N*-acetylneuraminic acid (DANA, **2**, Figure 1) have been of central importance.^4−12^ This culminated in the development of Relenza (**3**, Figure 1), a C4-modified analogue of **2** designed to mimic the transition state of **1** during the neuraminidase catalyzed hydrolysis reaction required for release of virus progeny from infected cells.^5^ C4-modified analogues of **2** including nitrogen,^4^ sulfur,^4^ and deoxygenated^7^ compounds are efficiently accessed via selective ring opening at position 5 of the allylic oxazoline of 2,3-didehydro-*N*-acetylneuraminic acid (**4**, Figure 1).^4^ However, in the case of oxygen nucleophiles, opening occurs at position 2 of the oxazoline ring.^7^ Hence, the method is not applicable to the synthesis of 4-*O*-modified analogues of **2** (or **1**) with retained stereochemistry. C4-deoxy and C4-nitrogen analogues of **1** can, however, be accessed using the ring-opening methodology but require reinstallment of the glycosidic bond, which produces two stereoisomers of equal proportions.^13,14^

**Figure 1:**
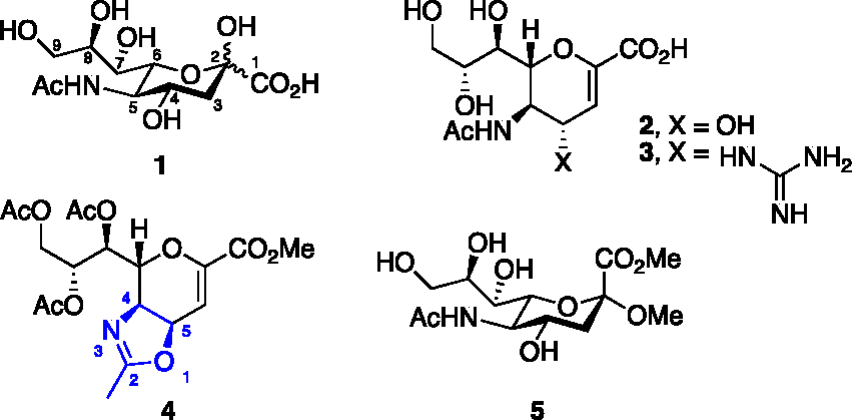
Structure of Neu5Ac and DANA analogues.

The interest in Neu5Ac analogues and their roles in biological systems is constantly increasing. Therefore, efficient methods that allow site-selective modifications of the Neu5Ac-template are of great utility for studying Neu5Ac biology and for drug discovery. Methods to selectively access C4-modified analogues of **1** are scarce, with relatively few reported examples. These include carba,^15,16^ keto,^16^ ether,^14,17−20^ nitrogen,^13^ and deoxygenated^21^ derivatives. A potential drawback in the development of direct methods could be the competing formation of intramolecular lactams^22−24^ and lactones^25^ that occur under both basic and acidic conditions. To date, examples of selectively 4-*O*-modified Neu5Ac analogues include 4-*O*-Ac, -benzyl,^26−30^ -allyl,^23^ -silyl,^15^ -methyl,^14,17,18^ -ethyl,^18^ -cyanomethyl,^19^ and -*tert*-butoxyacetate^20^ groups. The electrophiles used to produce these 4-*O*-modified analogues all have in common that they are activated, highly reactive, and (with a few exceptions) lack the presence of β-hydrogens. Further, the commercial availability of suitable electrophiles remains limited. Herein, we set out to study the scope and the 4-*O*-alkylation of Neu5Ac.

In an ongoing research project, we were interested in studying the potential of 4-*O*-alkyl analogues of **5** (Figure 1) as probes targeting cell attachment during adenovirus^31−33^ and coxsackievirus infections.^34,35^ We hypothesized that **6** (Scheme 1) would be a suitable substrate to study *O*-alkylation. This previously described protective group strategy is straightforward, high yielding, and allows removal of the protective groups in a final single step.^15^ Propargyl bromide was selected as the model electrophile. The lack of β-protons minimizes the competing E2 reaction, thus providing a fair measure of the effectiveness of the S_N_2 reaction. In addition, the generated alkynyl product can be further modified under mild conditions.^36,37^ Compound **5**(38) (Figure 1) was obtained from **1** and then converted by standard methods to the known derivative **7**(39) (Scheme 1). Treatment of **7** with wet trifluoroacetic acid in DCM afforded **9**, which upon acetylation gave the fully protected derivative **11** in 79% yield over two steps. Treatment of **11** with TBAF in THF afforded **6** in 81% yield.

**Scheme 1:**
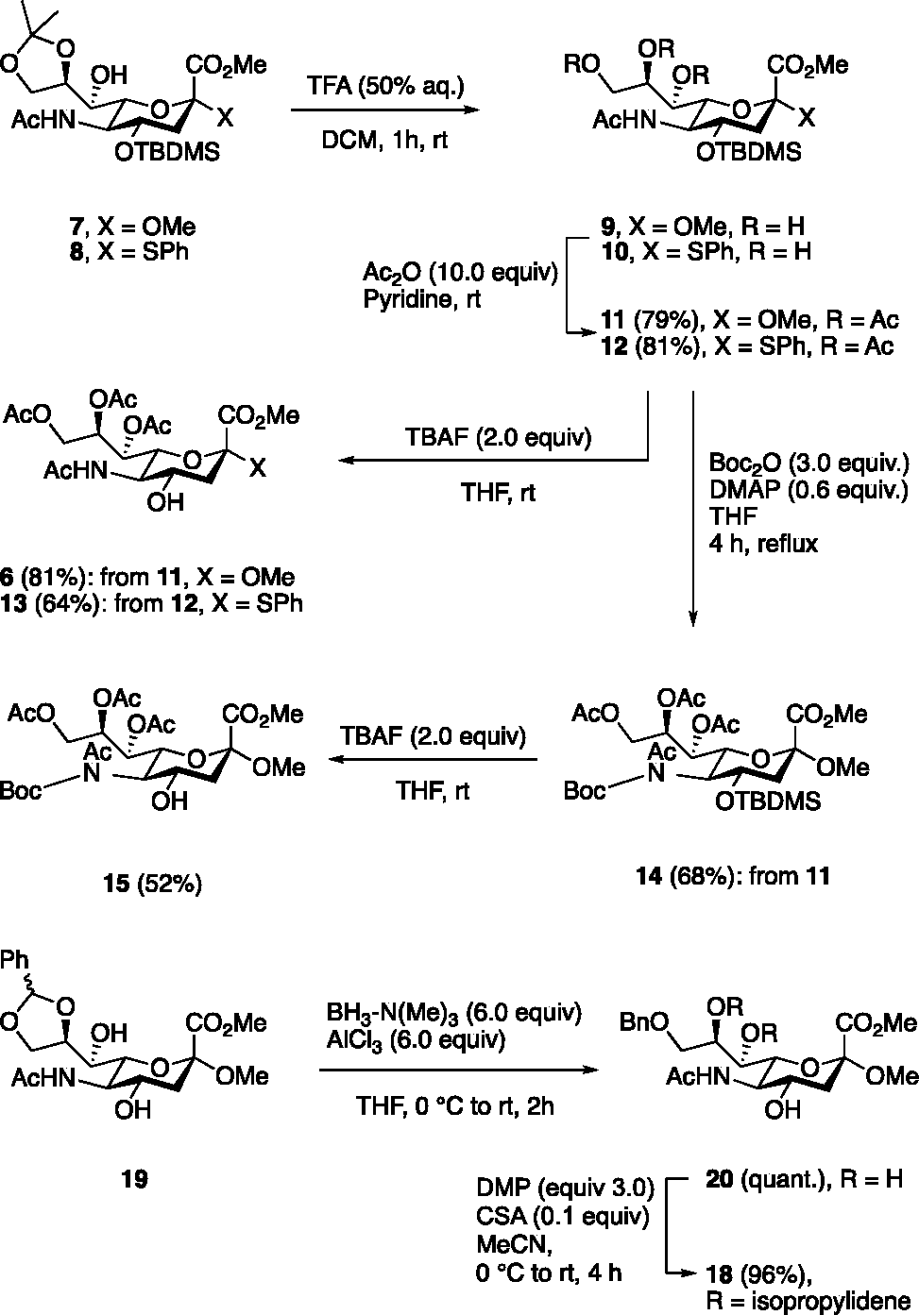
Synthesis of Neu5Ac Derivatives **13**, **15**, and **18** Selectively Protected at the 7-, 8-, and 9-Positions

Attempts to alkylate **6** using Ba(OH)_2_/BaO in DMF,^23,30,40^ K_2_CO_3_ in THF, or CsCO_3_ in MeCN, resulted in minimal amounts of **16**. However, promising observations were made in THF using KHMDS or NaH, with NaH providing superior conversion and product formation. Standard *O*-alkylation conditions were screened by treating **6** with NaH on ice prior to the addition of propargyl bromide (entries 1 and 2, Table 1). In DMF, this resulted in nearly complete decomposition and only trace amounts of **16** (entry 1). However, in THF, the 4-*O*-propargylated derivative **17** was isolated in 50% yield over two steps (entry 2). The *O*-deacetylation was performed to compensate for the formation of hydrolyzed species during the reaction (Figure S1) and, thus, facilitate isolation of the desired 4-*O*-alkyl product. The *O*-deacetylation of purified **16** gave **17** in 85% yield, the value that was used to estimate the yield for the *O*-alkylation (Table 1).

**Table 1:**
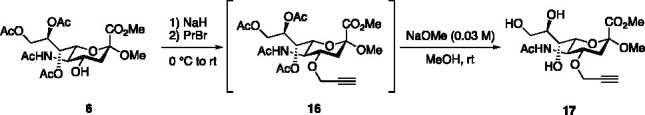

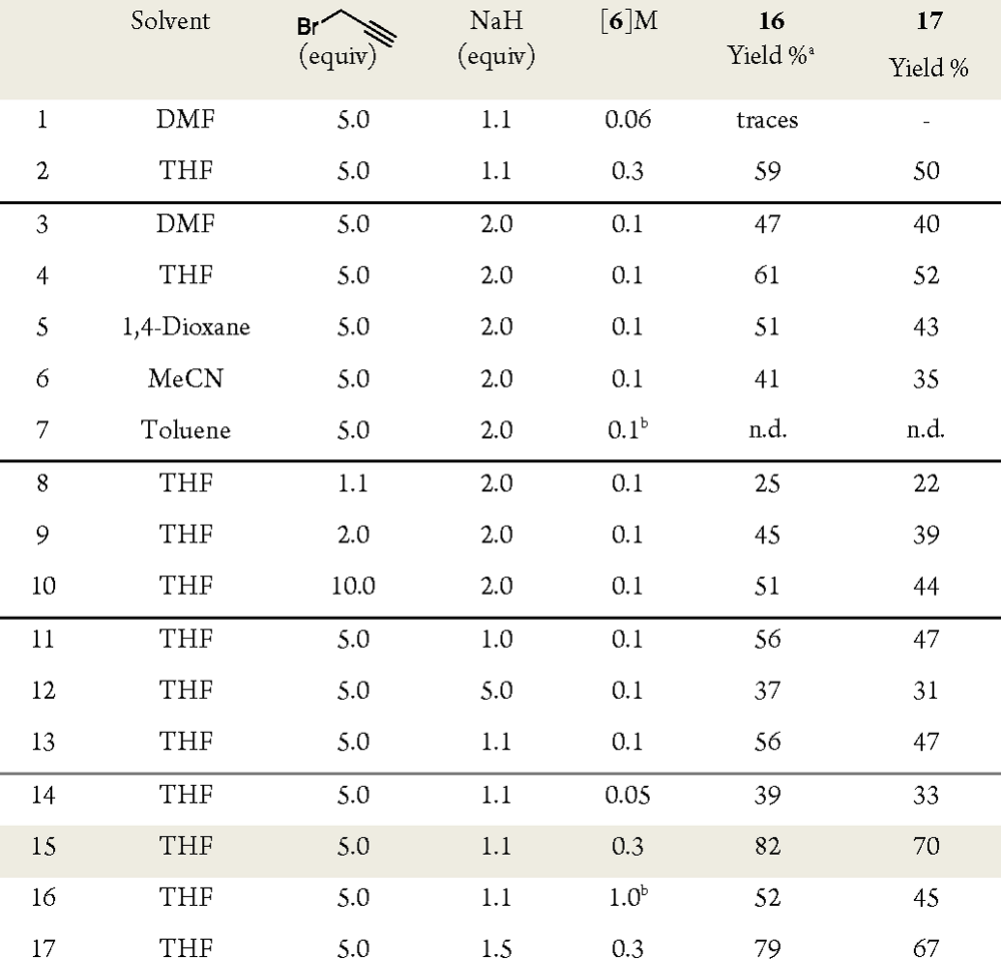
Screening and Optimization of Reaction Conditions

a Estimated yields are based on the isolated yield (85%) of the *O*-deacetylated **17**.

b Solubility issues; n.d. = not determined.

Alkoxide formation was studied by mixing compound **6** with NaH in DMF-*d*_7_ and in THF-*d*_8_, respectively, and recording ^1^H NMR spectra at two different 10 min and 1 h (Figure S2A–F). Within 10 min, compound **6** was essentially consumed in DMF-*d*_7_, resulting in a complex mixture of products (Figure S2A,B). In contrast, only minimal signs of degradation were observed in THF-*d*_8_ 10 min postaddition of NaH (Figure S2D,E), and the majority of **6** was largely intact after 1 h (Figure S2F).

This prompted us to reverse the addition order, and compound **6** was mixed with propargyl bromide in the selected solvent on ice before adding NaH. Furthermore, the stoichiometry of NaH was increased from 1.1 to 2.0 equiv to ensure complete deprotonation of both the hydroxyl and acetamide of **6**. These modifications drastically improved the yield of **17** in DMF (40%; entry 3, Table 1), while no significant effect was observed in THF (52% yield; entry 4). This highlights the importance of avoiding preformation of the alkoxide in DMF. The 4-*O*-alkylated product **17** was confirmed by 2D NMR analysis and by treatment with acetic anhydride in pyridine, which afforded **16** in 60% yield.

Common solvents for *O*-alkylation reactions were screened, and the yields of **17** were lower in both 1,4-dioxane (43%; entry 5, Table 1) and MeCN (35%; entry 6) compared to the reference reaction (entry 4). The reaction in toluene (entry 7) was slow, with incomplete conversion after 72 h of stirring, likely due to poor solubility, and was not processed further. Decreasing the stoichiometry of propargyl bromide to 1.1 and 2.0 equiv afforded **17** in 22% and 39% yields, respectively (entries 8 and 9), while increased stoichiometry gave **17** in 44% yield (10 equiv; entry 10) and resulted in a larger concentration of side products.

Reduced stoichiometry of NaH gave **17** in 47% yield (1.0 equiv; entry 11, Table 1) with incomplete conversion, while increased stoichiometry provided **17** in 31% yield (5.0 equiv; entry 12) with larger amounts of side products, suggesting the stoichiometry of NaH should be greater than one but less than two equivalents to ensure complete conversion and minimize the formation of side products. Indeed, 1.1 equiv of NaH gave a clean reaction and complete conversion, albeit without improvement of the yield (47%; entry 13). Decreased substrate concentration gave **17** in 33% yield (0.05 M; entry 14). Pleasingly, increased concentration produced **17** in 70% yield (0.3 M; entry 15), corresponding to a 35% improvement compared to the reference reaction. Higher concentration was associated with solubility issues but provided **17** in 45% yield (1.0 M; entry 16). With the optimized conditions in hand, the stoichiometry of NaH was adjusted to 1.5 equiv as the conversion was incomplete in some reactions when using 1.1 equiv. This resulted in complete conversion of **6**, providing **17** in 67% yield (entry 17). To conclude, the optimal conditions are a concentration of 0.3 M (in THF), 5.0 equiv of propargyl bromide, and 1.1–1.5 equiv of NaH.

In an attempt to further improve the yields of the *O*-alkylation, compounds **15** and **18** were prepared (Scheme 1). Compound **15** with its tertiary amide renders it resistant toward potential side products arising from lactamization.^22−24^ Compound **15** was accessed from **11** by treatment with Boc anhydride and DMAP in THF followed by cleavage of the TBDMS group using TBAF (Scheme 1). Surprisingly, the reactivity of **15** was completely abolished toward *O*-alkylation (entry 1, Table 2).

**Table 2:**
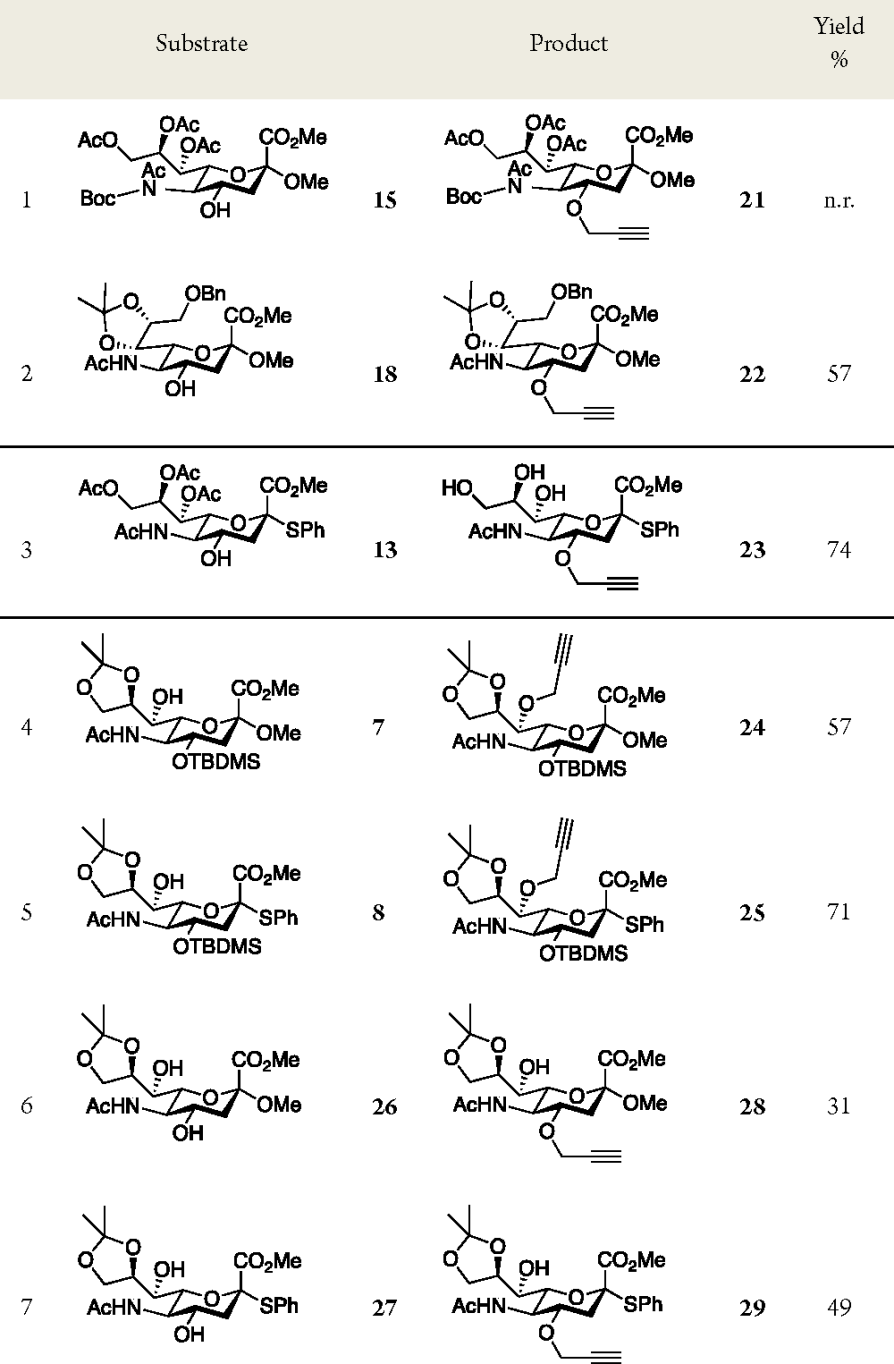
*O*-alkylation of Diversely Protected Neu5Ac Building Blocks^a^

a All reactions were conducted in THF (0.3 M substrate) and performed by treating a stirred solution of 5.0 equiv of propargyl bromide and substrate with 1.1–1.5 equiv of NaH (specific details in Supporting Information). n.r. = no reaction. Yield over two steps.

Prolonged reaction times (2.5 h), heating (60 °C for 16 h, with a heating mantle), and irradiation in a microwave reactor (100 °C for 20 min) were inefficient in causing conversion. Compound **18** was prepared from the known derivative **19**(41) including selective reduction with borane-trimethyl amine and aluminum chloride in THF affording **20** in quantitative yield that upon treatment with 2,2-dimethoxypropane and camphor sulfonic acid in MeCN gave the 9-*O*-benzyl-7,8-acetonide protected **18** in 96% yield. This protective group strategy is orthogonal allowing site-selective removal and functionalization of the glycerol side chain (C7, C8, and C9). Further, the protective groups have increased resistance toward hydrolysis under basic conditions. Upon *O*-alkylation **18** gave **22** in 57% yield (entry 2). Compound **13** was prepared in analogueous manner to **6** (Scheme 1), and upon *O*-alkylation afforded **23** in 74% yield (entry). Compound **23,** and analogues thereof, significantly broaden the scope due to their potential for modifications at the C2-position via glycosylation, or elimination to access 4-*O*-alkyl DANA analogues.^19^ The developed conditions were applied to synthetic intermediates **7** and **8** which provided **24** and **25** in 57% and 71% yields, respectively (entries 4 and 5). Thus, supporting access to 7-*O*-alkylated species. Synthetic intermediates **26** and **27** selectively afforded the 4-*O*-alkylated products **28** and **29** in 31% and 49% yields (entries 6 and 7), respectively. Thus, significantly decreasing the number of steps to access 4-*O*-alkylated analogues of **18.**

Representative examples of commercial alkyl halides and sulfonates were then screened to study the scope of the 4-*O*-alkylation of **6** (Figure 2). As expected, the activated alkyl bromides benzyl bromide, allyl bromide, and ethyl bromoacetate afforded the corresponding 4-*O*-ethers **30** (62% yield), **31** (52% yield), and **32** (78% yield) (Figure 2), respectively. An initial reaction with 6-iodo-1-hexyne gave **33** in a mere 10% yield. Dipolar aprotic solvents are known to increase the rate of substitution due to their ability to solvate cations,^42^ and the use of DMF indeed afforded **33** in 45% yield. Using 6-chloro-1-hexyne resulted in trace amounts of **33**, and the addition of TBAI, or KI, did not result in the isolation of **33** in either THF or DMF. Upon *O*-alkylation 5-bromo-1-pentene afforded **34** in 17% yield. Ethyl tosylate gave **35** in poor yield (8%) with substantial amounts of hydrolyzed starting material. However, propargyl mesylate gave **17** in 58% yield, supporting the use of activated alkyl sulfonates. Last, we attempted to substitute 2-bromopropane, which resulted in trace amounts of **36** (Figure 2), in line with the fact that 2° halides are less reactive in *O*-alkylation reactions due to excess β-protons favoring an E2 pathway over the desired substitution reaction.^43^

**Figure 2:**
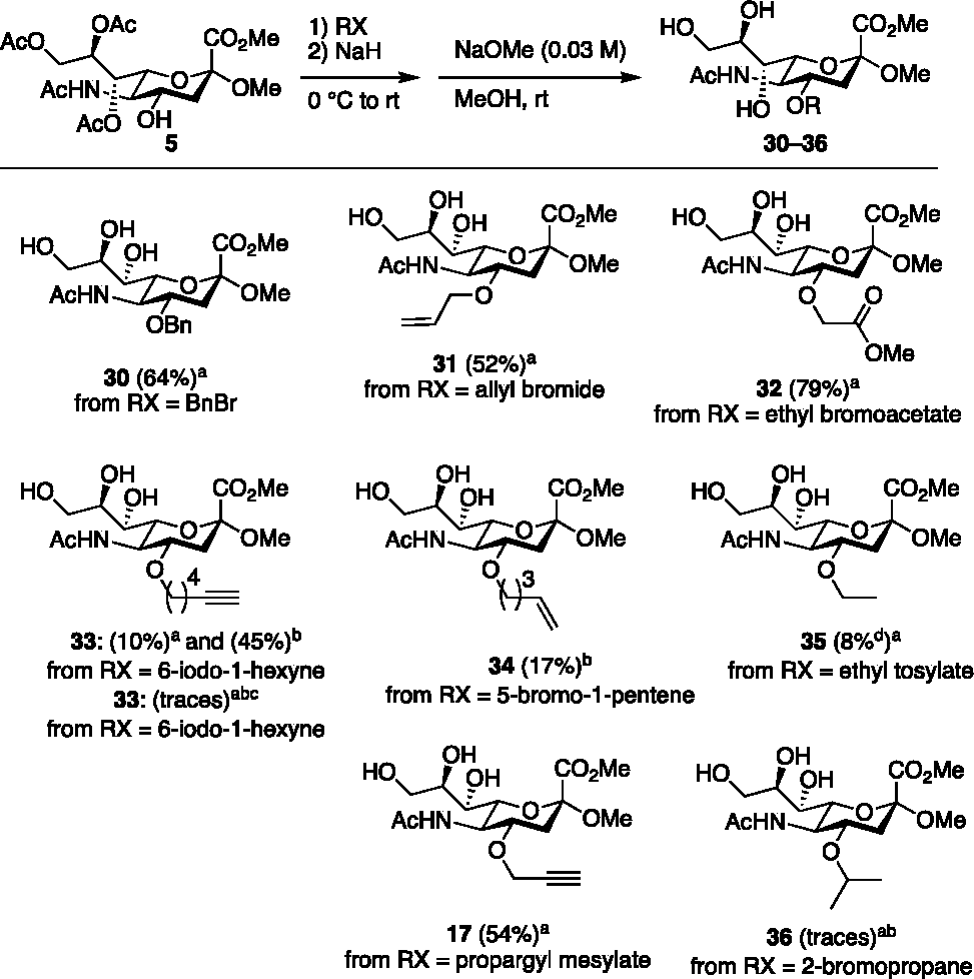
Scope of 4-*O*-alkylation. Outline of reaction (top). Synthesized substrates and used electrophiles (=RX). Footnote a represents THF as a solvent. Footnote b represents DMF as a solvent. Footnote c represents KI or TBAI as additives. Footnote d represents 80% pure.

To exemplify the utility of the developed methodology, we purified intermediate **37** and treated it with TfOH and NIS in DCM,^44^ affording the 4-*O*-propargyl DANA analogue **38** in 87% yield (Scheme 2), thus confirming access to 4-*O*-alkyl DANA analogues,^19^ via C3-elimination. These compounds have potential as antivirals toward human parainfluenza type-1.^45−50^

**Scheme 2:**
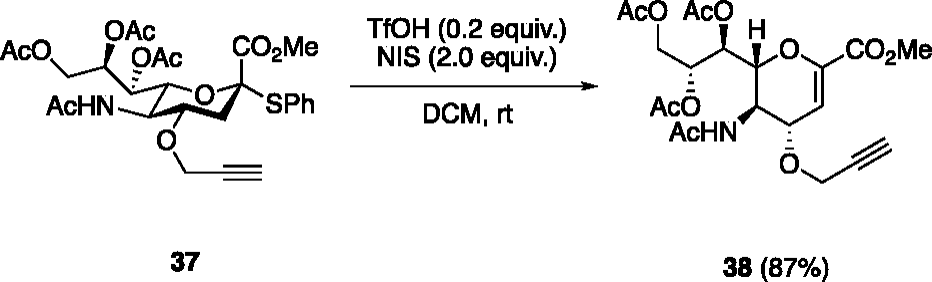
Synthesis of a 4-*O*-Propargyl DANA Analogue

In summary, we have systematically studied *O*-alkylation of Neu5Ac derivatives and provided insights into the scope of the reaction for preparation of tool compounds and starting points for drug discovery.^51^

## Experimental section

### General Chemical Procedures

^1^H NMR and ^13^C NMR spectra were recorded with a Bruker DRX-400 spectrometer at 400 and 100 MHz, respectively, or with a Bruker DRX-600 spectrometer at 600 and 150 MHz, respectively. NMR experiments were conducted at 298 K in CD_3_OD (residual solvent peak = 3.31 ppm, δH and 49.00 ppm, δC) or CDCl_3_ (residual solvent peak = 7.26 ppm, δH and 77.16 ppm, δC). Liquid chromatography–mass spectrometry (LC–MS) data were recorded by detecting positive/negative ions (electrospray ionization, ESI) on an Agilent 1290 Infinity II-6130 Quadrupole using H_2_O/CH_3_CN (0.1% formic acid) as the eluent system or on an Agilent 1290 Infinity-6150 Quadrupole using YMC Triart C18 (1.9 μm, 20 mm × 50 mm column) and H_2_O/CH_3_CN (0.1% formic acid) as the eluent system. High-resolution mass spectrometry (HRMS) data were recorded on an Agilent 1290 binary LC System connected to an Agilent 6230 Accurate-Mass Time-of-Flight (TOF) LC–MS (ESI+), which was calibrated with Agilent G1969–85001 ES-TOF Reference Mix containing ammonium trifluoroacetate, purine, and hexakis(1*H*,1*H*,3*H*-tetrafluoropropoxy)phosphazine in 90:10 CH_3_CN/H_2_O. Semipreparative high-performance liquid chromatography (HPLC) was performed on a Gilson system using a YMC-Actus Triart C18, 12 nm, S-5 μm, 250 mm × 20.0 mm with a flow rate of 20 mL min^–1^, detection at 214 nm, and eluent system A with aqueous 0.005% formic acid, and B with CH_3_CN 0.005% formic acid. Thin-layer chromatography (TLC) was performed on silica gel 60 F254 (Merck) with detection under ultraviolet (UV) light and/or development with 5% H_2_SO_4_ in EtOH and heat. Flash chromatography was performed using a Biotage Isolera One system and purchased prepacked silica gel cartridges (Biotage Sfär Silica). Lyophilization was performed by freezing the diluted CH_3_CN/water solutions in a dry ice–acetone bath or liquid nitrogen and then employing an Alpha 3–4 LSCbasic freeze-dryer. Organic solvents were dried using a Glass Contour Solvent System (SG Water USA). All commercial reagents were used as received. All target compounds were ≥95% pure according to HPLC UV traces, unless otherwise noted.

### General Procedure for *O*-Alkylation (GP1): Exemplified with the Synthesis of **16**

An oven-dried vial was charged with a magnetic stirring bar and compound **6** (40 mg, 0.086 mmol). The vial was placed under nitrogen, and THF (0.3 mL) followed by propargyl bromide (48 μL, 0.43 mmol, 5 equiv) were added. The mixture was cooled to an ice-bath temperature, and NaH (3.8 mg, 0.095 mmol, 1.1 equiv) was added in portions. After 10 min, the reaction was allowed to perform at room temperature for an additional 2 h (monitored by TLC/EtOAc; *R*_*f*_ = 0.43). After completion, the reaction was quenched by the addition of a few drops of sat. aq NH_4_Cl, and the solvents were removed under reduced pressure. The crude product was directly used in the deacetylation step, unless otherwise noted.

### General Procedure for Deacetylation (GP2): Exemplified with the Synthesis of **17**

Crude **16** was dissolved in dry methanol (4 mL), and sodium, methoxide (28 mg, 0.52 mmol, 6 equiv) was added in portions. The reaction was allowed to perform at room temperature for 1 h (monitored by TLC: Tol/CH_3_OH (4:1, v/v); *R*_*f*_ = 0.40). The mixture was then neutralized with Amberlyst H^+^-form, filtered, and concentrated to dryness. The compound was purified using flash chromatography (Tol/CH_3_OH, gradient 5–20%) to give compound **17**.

#### Chemical Synthesis

##### Methyl (Methyl 5-acetamido-4-*O*-(*tert*-butyldimethylsilyl)-3,5-dideoxy-8,9-*O*-isopropylidene-d-*glycero*-α-d-*galacto*-2-nonulopyranosid)onate (**7**).^1^

Compound **7** (1.39 g, 2.84 mmol, white foam) was synthesized in 71% yield following the procedure described in ref (1). ^1^H NMR (CDCl_3_, 600 MHz): δ 5.19 (d, 1H, *J* = 7.3 Hz), 4.32 (q, 1H, *J* = 6.4 Hz), 4.25 (d, 1H, *J* = 5.0 Hz), 4.12 (dd, 1H, *J* = 8.5, 6.3 Hz), 4.06 (dd, 1H, *J* = 8.4, 6.3 Hz), 3.85–3.73 (m, 2H), 3.80 (s, 3H), 3.57 (t, 1H, *J* = 5.7 Hz), 3.40 (s, 3H), 3.39 (d, 1H, *J* = 10.1 Hz), 2.59 (dd, 1H, *J* = 12.8, 4.2 Hz), 2.02 (s, 3H), 1.82 (dd, 1H, *J* = 12.5, 10.9 Hz), 1.37 (s, 3H), 1.36 (s, 3H), 0.87 (s, 9H), 0.10 (s, 3H), 0.09 (s, 3H). ^13^C{^1^H} NMR (CDCl_3_, 150 MHz): δ 172.5, 169.1, 108.7, 99.3, 75.4, 74.4, 70.1, 69.0, 67.0, 53.7, 52.5, 51.8, 40.6, 27.0, 25.7, 23.3, 18.0, −3.9, −4.6. HRMS (ESI-TOF) *m*/*z*: [M + Na]^+^ calcd for C_22_H_41_NO_9_SiNa, 514.2443; found, 514.2467.

##### Methyl (Phenyl 5-acetamido-4-*O*-(*tert*-butyldimethylsilyl)-3,5-dideoxy-8,9-*O*-isopropylidene-2-thio-d-*glycero*-α-d-*galacto*-2-nonulopyranosid)onate (**8**).^2^

Compound **8** (0.78 g, 1.89 mmol, pale-yellow foam) was synthesized in 73% yield following the procedure described in ref (2). ^1^H NMR (CD_3_OD, 600 MHz): δ 7.58 (dd, 2H, *J* = 8.3, 1.4 Hz, 2H), 7.42 (t, 1H, *J* = 7.2 Hz), 7.36 (t, 2H, *J* = 7.6 Hz), 4.15 (q, 1H, *J* = 6.7 Hz), 3.98 (dd, 1H, *J* = 8.2, 6.3 Hz), 3.88 (dd, 1H, *J* = 8.5, 6.7 Hz), 3.91–3.82 (m, 1H), 3.77–3.68 (m, 1H), 3.50 (d, 1H, *J* = 7.1 Hz), 3.48 (s, 3H), 3.45 (d, 1H, *J* = 10.6 Hz), 2.72 (dd, 1H, *J* = 12.7, 4.7 Hz), 1.92 (s, 3H), 1.76 (dd, 1H, *J* = 12.7, 11.0 Hz), 1.31 (s, 3H), 1.23 (s, 3H), 0.88 (s, 9H), 0.09 (s, 3H), 0.06 (s, 3H). ^13^C{^1^H} NMR (CD_3_OD, 150 MHz): δ 173.7, 170.5, 138.0, 137.9, 130.9, 130.8, 129.7, 109.9, 88.4, 77.1, 76.8, 71.1, 70.7, 68.0, 53.3, 52.6, 42.8, 27.0, 26.1, 25.7, 23.0, 18.7, −4.3, −4.7. HRMS (ESI-TOF) *m*/*z*: [M + H]^+^ calcd for C_27_H_44_NO_8_SSi, 570.2551; found, 570.2577.

##### Methyl (Methyl 5-acetamido-7,8,9-tri-*O*-acetyl-4-*O*-(*tert*-butyldimethylsilyl)-3,5-dideoxy-d-*glycero*-α-d-*galacto*-2-nonulopyranosid)onate (**11**)

To a solution of **7** (3.45 g, 7 mmol) in dichloromethane (130 mL) was added 50% aqueous TFA (5.1 mL, 33.3 mmol, 4.75 equiv). The reaction was stirred at room temperature for 1 h (monitored by TLC/EtOAc/CH_3_OH/H_2_O (10:2:1, v/v/v)). After completion, triethylamine (3 mL) was added to the mixture, and the solvents were concentrated to dryness. The residue was coevaporated three times with toluene and directly acetylated. Crude compound **9** was dissolved in pyridine (35 mL) and treated with an excess of acetic anhydride (10 mL, 105 mmol, 15 equiv). The reaction was allowed to perform at room temperature and under a nitrogen atmosphere overnight. After completion, the mixture was coevaporated three times with toluene. The residual oil was purified using flash chromatography (petroleum ether/EtOAc, gradient 5–70%) to give **11** (3.2 g, 79%) as an off-white solid. ^1^H NMR (CDCl_3_, 600 MHz,): δ 5.36 (ddd, 1H, *J* = 8.8, 5.7, 2.8 Hz), 5.29 (dd, 1H, *J* = 8.7, 2.3 Hz), 4.29 (dd, 1H, *J* = 12.4, 2.8 Hz), 4.03 (dd, 1H, *J* = 12.4, 5.7 Hz), 3.97 (dd, 1H, *J* = 11.0, 2.1 Hz), 3.81–3.72 (m, 1H), 3.78 (s, 3H), 3.52–3.44 (m, 1H), 3.24 (s, 3H), 2.49 (dd, 1H, *J* = 12.8, 4.32 Hz), 2.10 (s, 3H), 2.07 (s, 3H), 1.97 (s, 3H), 1.83 (s, 3H), 1.65 (dd, 1H, *J* = 12.85, 11.83 Hz), 0.83 (s, 9H), 0.09 (s, 3H), 0.00 (s, 3H). ^13^C{^1^H} NMR (CDCl_3_, 150 MHz): δ 173.1, 172.4, 171.8, 171.7, 169.6, 100.4, 73.3, 70.6, 69.8, 68.9, 63.6, 53.0, 52.6, 52.5, 43.0, 26.1, 23.1, 21.2, 20.9, 20.6, 18.7, −4.5, −4.8. HRMS (ESI-TOF) *m*/*z*: [M + Na]^+^ calcd for C_25_H_43_NO_12_SiNa, 600.2447; found, 600.2471.

##### Methyl (Phenyl 5-acetamido-7,8,9-tri-*O*-acetyl-4-*O*-(*tert*-butyldimethylsilyl)-3,5-dideoxy-2-thio-d-*glycero*-α-d-*galacto*-2-nonulopyranosid)onate (**12**)

To a solution of **8** (650 mg, 1.14 mmol) in dichloromethane (20 mL) was added 50% aqueous TFA (0.83 mL, 5.4 mmol, 4.75 equiv). The reaction was stirred at room temperature for 30 min (monitored by TLC/EtOAc/CH_3_OH/H_2_O (10:2:1, v/v/v)). After completion, triethylamine (0.5 mL) was then added to the mixture, and the solvents were concentrated to dryness. The residue was coevaporated three times with toluene and directly acetylated. Crude compound **10** was dissolved in pyridine (7 mL) and treated with an excess of acetic anhydride (1.6 mL, 17 mmol, 15 equiv). The reaction was allowed to perform at room temperature and under a nitrogen atmosphere overnight. After completion, the mixture was coevaporated three times with toluene. The residual oil was purified using flash chromatography (petroleum ether/EtOAc, gradient 5–70%) to give **12** (605 mg, 81%) as a pale-yellow solid. ^1^H NMR (CDCl_3_, 600 MHz): δ 7.50 (dd, 2H, *J* = 8.2, 1.4 Hz), 7.37 (d, 1H, *J* = 7.0 Hz), 7.32 (t, 2H, *J* = 7.5 Hz), 5.29 (dd, 1H, *J* = 7.3, 1.8 Hz), 5.25 (ddd, 1H, *J* = 7.1, 5.4, 2.6 Hz), 5.18 (d, 1H, *J* = 9.1 Hz), 4.38 (dd, 1H, *J* = 12.5, 2.6 Hz), 4.25 (dd, 1H, *J* = 12.5, 5.4 Hz), 4.06 (dd, 1H, *J* = 10.8, 1.8 Hz), 3.88 (dt, 1H, *J* = 10.3, 4.4 Hz), 3.53 (s, 3H), 3.37 (q, 1H, *J* = 9.6 Hz), 2.78 (dd, 1H, *J* = 13.0, 4.5 Hz), 2.17 (s, 3H), 2.05 (s, 3H), 2.02 (s, 3H), 1.91 (s, 3H), 1.84 (dd, 1H, *J* = 12.9, 11.2 Hz), 0.85 (s, 9H), 0.05 (s, 3H), 0.01 (s, 3H). ^13^C{^1^H} NMR (CDCl_3_, 150 MHz): δ 136.4, 129.8, 129.2, 128.9, 87.7, 73.7, 70.1, 68.6, 68.5, 62.2, 53.5, 52.5, 42.4, 25.7, 23.8, 21.1, 21.0, 18.0, −4.4, −4.8. HRMS (ESI-TOF) *m*/*z*: [M + H]^+^ calcd for C_30_H_46_NO_11_SSi, 656.2555; found, 656.2584.

##### Methyl (Methyl 5-acetamido-7,8,9-tri-*O*-acetyl-3,5-dideoxy-d-*glycero*-α-d-*galacto*-2-nonulopyranosid)onate (**6**)

**C**ompound **11** (3.2 g, 5.54 mmol) was dissolved in dry THF (73 mL), and the mixture was stirred at room temperature under a nitrogen atmosphere. A 1 M TBAF solution in THF (11 mL, 11 mmol, 2 equiv) was then added to the reaction. After 1.5 h, the reaction was complete, and the solvents were concentrated to dryness. The residue was purified using flash chromatography (CH_2_Cl_2_/CH_3_OH, gradient 0–8%) to give **6** (2.07 g, 81%) as a white solid. ^1^H NMR (CD_3_OD, 600 MHz): δ 5.40 (ddd, 1H, *J* = 8.7, 5.8, 2.8 Hz), 5.30 (dd, 1H, *J* = 8.6, 2.3 Hz), 4.33 (dd, 1H, *J* = 12.4, 2.8 Hz), 4.06 (dd, 1H, *J* = 12.4, 5.7 Hz), 4.03 (dd, 1H, *J* = 10.7, 2.3 Hz), 3.80 (s, 3H), 3.75 (t, 1H, *J* = 10.3 Hz), 3.41 (ddd, 1H, *J* = 12.2, 10.0, 4.4 Hz), 3.27 (s, 3H), 2.56 (dd, 1H, *J* = 12.9, 4.4 Hz), 2.13 (s, 3H), 2.10 (s, 3H), 2.01 (s, 3H), 1.90 (s, 3H), 1.67 (*app*t, 1H, *J* = 12.5 Hz). ^13^C{^1^H} NMR (CD_3_OD, 150 MHz): δ 173.8, 172.4, 171.8, 171.7, 169.8, 100.6, 73.4, 69.8, 69.2, 69.0, 63.6, 53.0, 52.8, 52.5, 42.0, 22.9, 21.2, 20.9, 20.6. HRMS (ESI-TOF) *m*/*z*: [M + Na]^+^ calcd for C_19_H_29_NO_12_Na, 486.1582; found, 486.1604.

##### Methyl (Phenyl 5-acetamido-7,8,9-tri-*O*-acetyl-3,5-dideoxy-2-thio-d-*glycero*-α-d-*galacto*-2-nonulopyranosid)onate (**13**)

Compound **12** (590 mg, 0.90 mmol) was dissolved in dry THF (12 mL), and the mixture was stirred at room temperature under a nitrogen atmosphere. A 1 M TBAF solution in THF (1.8 mL, 1.8 mmol, 2 equiv) was then added to the reaction. After 50 min, the reaction was complete, and the solvents were concentrated to dryness. The residue was purified using flash chromatography (Tol/CH_3_OH, 5–12%) to give **13** (310 mg, 64%) as a pale-yellow solid. ^1^H NMR (CD_3_OD, 600 MHz,): δ 7.52 (dd, 2H, *J* = 8.0, 1.4 Hz), 7.40 (d, 1H, *J* = 7.4 Hz), 7.35 (t, 2H, *J* = 7.5 Hz), 5.29 (dd, 1H, *J* = 7.5, 1.9 Hz), 5.26 (ddd, 1H, *J* = 7.7, 5.3, 2.6 Hz), 4.40 (dd, 1H, *J* = 12.4, 2.6 Hz), 4.14 (dd, 1H, *J* = 12.4, 5.3 Hz), 3.83 (dd, 1H, *J* = 10.7, 1.8 Hz), 3.70 (t, 1H, *J* = 10.3 Hz), 3.53 (s, 3H), 3.41 (ddd, 1H, *J* = 11.4, 10.0, 4.6 Hz), 2.80 (dd, 1H, *J* = 13.0, 4.5 Hz), 2.12 (s, 3H), 2.03 (s, 3H), 2.01 (s, 3H), 1.89 (s, 3H), 1.80 (dd, 1H, *J* = 13.0, 11.5 Hz). ^13^C{^1^H} NMR (CD_3_OD, 150 MHz): δ 173.8, 172.5, 171.7, 171.6, 169.7, 137.4, 130.8, 130.6, 130.0, 89.1, 75.7, 71.1, 69.8, 69.3, 63.2, 53.0, 52.6, 42.4, 22.9, 21.0, 20.9, 20.7. HRMS (ESI-TOF) *m*/*z*: [M + H]^+^ calcd for C_24_H_32_NO_11_S, 542.1691; found, 542.1717.

##### Methyl (Methyl 5-(*N*-*tert*-butoxycarbonylacetamido)-7,8,9-tri-*O*-acetyl-4-*O*-(*tert*-butyldimethylsilyl)-3,5-dideoxy-d-*glycero*-α-d-*galacto*-2-nonulopyranosid)onate (**14**)

Compound **11** (500 mg, 0.87 mmol), di-*tert*-butyl dicarbonate (567 mg, 2.6 mmol, 3 equiv), and DMAP (63.4 mg, 0.52 mmol, 0.6 equiv) were charged in a round-bottom flask, and dry THF (14 mL) was added. The mixture was heated to reflux temperature for 8 h (monitored by TLC: petroleum ether/EtOAc (3:2, v/v), *R*_*f*_ = 0.50). The mixture was then diluted in diethyl ether (40 mL), washed with 0.5 M HCl (aq,15 mL), and sat. NaHCO_3_ (aq, 15 mL). The organic layer was dried over anhydrous sodium sulfate, filtered, and concentrated to dryness. The residue was purified using flash chromatography (petroleum ether/EtOAc, gradient 5–45%) to give **14** (400 mg, 68%) as a white solid. In ^1^H and ^13^C NMR experiments, a splitting of the signals is observed due to the presence of *N*-Boc rotamers (2:5 ratio). Chemical shifts are only reported for the main rotamer. ^1^H NMR (CD_3_OD, 600 MHz): δ 3.97–3.73 (m, 8H), 3.70–3.55 (m, 12H), 3.50 (dd, 1H, *J* = 6.6, 1.7 Hz), 3.38 (t, 2H, *J* = 4.9 Hz), 2.70 (dd, 1H, *J* = 12.8, 4.6 Hz), 2.00 (s, 3H), 1.75 (t, 1H, *J* = 12.3 Hz). ^13^C{^1^H} NMR (CD_3_OD, 150 MHz): δ 175.2, 172.4, 171.8, 171.6, 169.5, 153.9, 100.4, 85.9, 71.8, 69.8, 68.2, 67.6, 62.9, 56.4, 53.2, 52.5, 44.4, 28.4, 28.3, 27.3, 26.3, 26.0, 21.4, 21.0, 20.6, −4.1, −4.6. HRMS (ESI-TOF) *m*/*z*: [M + Na]^+^ calcd for C_30_H_51_NO_14_SiNa, 700.2971; found, 700.3003.

##### Methyl (Methyl 5-(*N*-*tert*-butoxycarbonylacetamido)-7,8,9-tri-*O*-acetyl-3,5-dideoxy-d-*glycero*-α-d-*galacto*-2-nonulopyranosid)onate (**15**)

Compound **14** (400 mg, 0.59 mmol) was dissolved in dry THF (7.7 mL), and the mixture was stirred at room temperature under a nitrogen atmosphere. A 1 M TBAF solution in THF (1.2 mL, 1.2 mmol, 2 equiv) was then added to the reaction. After 1 h, the reaction was complete, and the solvents were concentrated to dryness. The residue was purified using flash chromatography (Tol/CH_3_OH, 4–10%) to give **15** (172 mg, 52%) as a white solid. ^1^H NMR (CDCl_3_, 600 MHz): δ 5.48–5.40 (m, 2H), 4.78 (ddd, 1H, *J* = 12.3, 10.4, 4.6 Hz), 4.29 (dd, 1H, *J* = 12.6, 2.4 Hz), 4.19 (d, 1H, *J* = 10.3 Hz), 4.10 (dd, 1H, *J* = 12.4, 4.5 Hz), 4.04 (dd, 1H, *J* = 11.0, 2.0 Hz), 3.80 (s, 3H), 3.78 (q, 1H, *J* = 10.6 Hz), 3.31 (s, 3H), 2.58 (dd, 1H, *J* = 12.8, 4.6 Hz), 2.14 (s, 3H), 2.13 (s, 3H), 2.04 (s, 3H), 2.03 (s, 3H), 1.90 (*app*t, 1H, *J* = 12.7 Hz), 1.39 (s, 9H). ^13^C{^1^H} NMR (CDCl_3_, 150 MHz): δ 170.9, 170.8, 170.1, 169.9, 168.3, 155.3, 99.1, 80.3, 72.8, 69.5, 68.3, 67.5, 62.7, 52.9, 52.6, 50.8, 38.2, 28.3, 21.3, 21.0. HRMS (ESI-TOF) *m*/*z*: [M + Na]^+^ calcd for C_24_H_37_NO_14_Na, 586.2106; found, 586.2130.

##### Methyl (Methyl 5-acetamido-7,8,9-tri-*O*-acetyl-3,5-dideoxy-4-propargyloxy-d-*glycero*-α-d-*galacto*-2-nonulopyranosid)onate (**16**)

Compound **17** (30 mg, 0.08 mmol) was dissolved in pyridine (1.3 mL) and treated with an excess of acetic anhydride (0.15 mL, 1.6 mmol, 20 equiv). The reaction was allowed to perform at room temperature and under a nitrogen atmosphere overnight. The mixture was diluted in EtOAc (10 mL), washed with 1 M HCl (aq, 5 mL) twice, and then with brine (5 mL). The organic layer was dried over anhydrous sodium sulfate, filtered, and concentrated to dryness to give semipure **16** (24 mg, 60%) as a colorless film. Analytical samples were obtained after purification of the semipure product using reverse-phase HPLC (H_2_O/acetonitrile (0.005% formic acid), gradient 15–80% over 25 min). ^1^H NMR (CDCl_3_, 600 MHz): δ 5.44 (ddd, 1H, *J* = 8.5, 5.5, 2.7 Hz), 5.39–5.26 (m, 2H), 4.33 (dd, 1H. *J* = 12.5, 2.6 Hz), 4.27 (dd, 1H, *J* = 10.7, 2.1 Hz), 4.23 (dd, 1H, *J* = 16.1, 2.3 Hz), 4.16 (dd, 1H, *J* = 12.5, 5.5 Hz), 4.13 (dd, 1H, *J* = 16.0, 2.3 Hz), 3.88 (dd, 1H, *J* = 11.8, 9.7, 4.5 Hz), 3.82 (s, 3H), 3.49 (t, 1H, *J* = 2.4 Hz), 3.32 (s, 3H), 2.76 (dd, 1H, *J* = 12.8, 4.5 Hz), 2.42 (t, 1H, *J* = 2.4 Hz), 2.15 (s, 3H), 2.14 (s, 3H), 2.04 (s, 3H), 1.97 (s, 3H), 1.68 (dd, 1H, *J* = 12.8, 11.9 Hz). ^13^C{^1^H} NMR (CDCl_3_, 150 MHz): δ 170.8, 170.7, 170.2, 168.2, 99.3, 79.8, 74.7, 73,1, 71.5, 68.6, 68.0, 62.6, 56.6, 52.7, 52.5, 51.4, 37.9, 23.9, 21.3, 21.1, 20.9. HRMS (ESI-TOF) *m*/*z*: [M + Na]^+^ calcd for C_22_H_31_NO_12_Na, 524.1738; found, 524.1756.

##### Methyl (Methyl 5-acetamido-3,5-dideoxy-4-propargyloxy-d-*glycero*-α-d-*galacto*-2-nonulopyranosid)onate (**17**)

Compound **17** was synthesized starting from **6** (40 mg, 0.086 mmol) using the general procedure for *O*-alkylation (GP1; TLC/EtOAc; *R*_*f*_ = 0.43) and the general procedure for deacetylation (GP2; TLC: Tol/CH_3_OH (4:1, v/v); *R*_*f*_ = 0.40), successively. The compound was purified using flash chromatography (Tol/CH_3_OH, gradient 5–20%) to give **17** as a white solid and in 70% yield. ^1^H NMR (CD_3_OD, 600 MHz): δ 4.27–4.19 (m, 2H), 3.90–3.80 (m, 3H), 3.85 (s, 3H), 3.74–3.62 (m, 3H), 3.51 (dd, 1H, *J* = 9.0, 1.6 Hz), 3.35 (s, 3H), 2.91 (t, 1H, *J* = 2.5 Hz), 2.86 (dd, 1H, *J* = 12.9, 4.6 Hz), 1.98 (s, 3H), 1.65 (dd, 1H, *J* = 12.9, 11.7 Hz). ^13^C{^1^H} NMR (CD_3_OD, 150 MHz): δ 174.7, 170.7, 100.3, 80.7, 76.1, 75.8, 74.6, 72.4, 70.1, 64.7, 57.6, 53.3, 52.0, 51.9, 38.5, 22.7. HRMS (ESI-TOF) *m*/*z*: [M + Na]^+^ calcd for C_16_H_25_NO_9_Na, 398.1422; found, 398.1438.

##### Methyl (Methyl 5-acetamido-9-*O*-benzyl-3,5-dideoxy-d-*glycero*-α-d-*galacto*-2-nonulopyranosid)onate (**20**).^3^

Compound **20** was synthesized in a quantitative yield following the procedure described in ref (3). ^1^H NMR (CD_3_OD, 600 MHz): δ 7.36 (d, 1H *J* = 7.6 Hz), 7.33 (t, 2H, *J* = 7.3 Hz), 7.26 (t, 2H, *J* = 7.2 Hz), 4.59 (ABq, 2H, *J* = 12.2 Hz), 3.99 (ddd, 1H, *J* = 9.1, 5.9, 2.3 Hz), 3.83 (s, 3H), 3.79 (dd, 1H *J* = 10.4, 2.4 Hz), 3.76 (t, 1H, *J* = 10.3 Hz), 3.68–3.61 (m, 2H), 3.61–3.56 (m, 2H), 3.32 (s, 3H), 2.65 (dd, 1H, *J* = 12.9, 4.6 Hz), 1.99 (s, 3H), 1.71 (dd, 1H, *J* = 12.9, 11.9 Hz). ^13^C{^1^H} NMR (CD_3_OD, 150 MHz): δ 175.1, 170.8, 139.8, 129.3, 128.8, 128.6, 100.3, 74.7, 74.4, 73.0, 71.4, 70.1, 68.5, 53.8, 53.3, 52.0, 41.4, 22.7. HRMS (ESI-TOF) *m*/*z*: [M + Na]^+^ calcd for C_20_H_29_NO_9_Na, 450.1735; found, 450.1743.

##### Methyl (Methyl 5-acetamido-9-benzyloxy-3,5-dideoxy-7,8-*O*-isopropylidene-d-*glycero*-α-d-*galacto*-2-nonulopyranosid)onate (**18**)

Compound **20** (307 mg, 0.72 mmol) and (1*R*)-(−)-10-camphorsulfonic acid (17 mg, 0.072 mmol, 0.1 equiv) were dissolved in freshly distilled acetonitrile (1.45 mL). The stirring mixture was cooled to an ice bath temperature, and 2,2-dimethoxypropane (0.26 mL, 2.15 mmol, 3.0 equiv) was added under a nitrogen atmosphere. The reaction mixture was then allowed to warm to room temperature, and after 4 h, triethylamine was added. The volatiles were removed under a vacuum, and the residue was purified using flash chromatography (Tol/acetone, isocratic 7:3) to afford **18** (323 mg, 96%). ^1^H NMR (CD_3_OD, 600 MHz): δ 7.38 (d, 2H, *J* = 7.4 Hz), 7.32 (t, 2H, *J* = 7.4 Hz), 7.26 (t, 1H, *J* = 7.3 Hz), 4.63 (ABq, 2H, *J* = 12.3 Hz), 4.50 (td, 1H, *J* = 7.1, 4.3 Hz), 4.17 (d, 1H, *J* = 6.9 Hz), 4.02 (dd, 1H, *J* = 10.4, 7.6 Hz), 3.96 (dd, 1H, *J* = 10.5, 4.3 Hz), 3.84–3.78 (m, 1H), 3.50 (ddd, 1H, *J* = 12.4, 10.0, 4.3 Hz), 2.52 (dd, 1H, *J* = 12.6, 4.4 Hz), 1.94 (s, 3H), 1.59 (*app*t, 1H, *J* = 12.5 Hz), 1.48 (s, 3H), 1.34 (s, 3H). ^13^C{^1^H} NMR (CD_3_OD, 150 MHz): δ 173.8, 170.3, 139.6, 129.4, 129.1, 128.7, 110.4, 100.5, 77.8, 76.0, 74.2, 74.1, 70.2, 68.4, 54.2, 52.8, 52.3, 41.7, 26.7, 26.0, 23.0. HRMS (ESI-TOF) *m*/*z*: [M + Na]^+^ calcd for C_23_H_33_NO_9_Na, 490.2048; found, 490.2062.

##### Methyl (Methyl 5-acetamido-9-benzyloxy-3,5-dideoxy-7,8-*O*-isopropylidene-4-propargyloxy-d-*glycero*-α-d-*galacto*-2-nonulopyranosid)onate (**22**)

Compound **22** was synthesized starting from **18** (40.3 mg, 0.086 mmol) using the general procedure for *O*-alkylation (GP1) with 1.1 equiv of NaH and 5 equiv of propargyl bromide (TLC: petroleum ether/EtOAc (1:4, v/v); *R*_*f*_ = 0.2). The compound was purified using flash chromatography (petroleum ether/EtOAc, gradient 30–90%) to give **22** (24.9 mg, 57%) as a white solid. ^1^H NMR (CD_3_OD, 600 MHz): δ 7.38 (d, 2H, *J* = 7.5 Hz), 7.33 (t, 2H, *J* = 7.6 Hz), 7.27 (t, 1H, *J* = 7.3 Hz), 4.63 (ABq, 2H, *J* = 12.1 Hz), 4.50 (dt, 1H, *J* = 7.2, 4.4, 1H), 4.19–4.15 (m, 3H), 4.01 (dd, 1H, *J* = 10.4, 7.5 Hz), 3.96 (dd, 1H, *J* = 10.3, 4.5 Hz), 3.86 (t, 1H, *J* = 10.2 Hz), 3.79 (s, 3H), 3.78 (d, 1H, *J* = 6.9 Hz), 3.61 (ddd, 1H, *J* = 12.1, 10.1, 4.3 Hz), 3.20 (s, 3H), 2.86 (t, 1H, *J* = 2.4 Hz), 2.73 (dd, 1H, *J* = 12.6, 4.3 Hz), 1.92 (s, 3H), 1.50 (dd, 1H, *J* = 12.9, 12.0 Hz), 1.47 (s, 3H), 1.34 (s, 3H). ^13^C{^1^H} NMR (CD_3_OD, 150 MHz): δ 177.2, 173.5, 170.1, 139.6, 129.3, 129.1, 128.7, 110.5, 100.5, 80.7, 77.7, 76.0, 75.9, 75.1, 74.2, 74.0, 70.2, 57.4, 52.9, 52.4, 52.3, 38.9, 26.7, 25.9, 23.0. HRMS (ESI-TOF) *m*/*z*: [M + Na]^+^ calcd for C_26_H_35_NO_9_Na, 528.2204; found, 528.2218.

##### Methyl (Phenyl 5-acetamido-3,5-dideoxy-4-propargyloxy-2-thio-d-*glycero*-α-d-*galacto*-2-nonulopyranosid)onate (**23**)

Compound **23** was synthesized starting from **13** (46.7 mg, 0.086 mmol) using the general procedure for *O*-alkylation (GP1 with 1.5 equiv of NaH and 5 equiv of propargyl bromide; TLC/EtOAc; *R*_*f*_ = 0.45) and the general procedure for deacetylation (GP2; TLC: Tol/CH_3_OH (4:1, v/v); *R*_*f*_ = 0.40), successively. The compound was purified using flash chromatography (CH_2_Cl_2_/CH_3_OH, gradient 0–10%) to give **23** (26.5 mg, 74%) as a pale-yellow solid. ^1^H NMR (CD_3_OD, 600 MHz): δ 7.56 (dd, 2H, *J* = 8.0, 1.4 Hz), 7.43 (dd, 1H, *J* = 7.8, 7.0 Hz), 7.37 (t, 2H, *J* = 7.5 Hz), 4.25 (d, 2H, *J* = 2.4 Hz), 3.90 (t, 1H, *J* = 10.4 Hz), 3.82–3.75 (m, 2H), 3.70–3.58 (m, 2H), 3.65 (s, 3H), 3.51–3.44 (m, 2H), 3.09 (dd, 1H, *J* = 12.9, 4.7 Hz), 2.92 (t, 1H, *J* = 2.41 Hz), 1.96 (s, 3H), 1.80 (dd, 1H, *J* = 12.9, 11.3 Hz). ^13^C{^1^H} NMR (CD_3_OD, 150 MHz): δ 174.7, 170.9, 137.9, 131.2, 130.1, 129.9, 87.9, 80.6, 77.2, 76.3, 76.2, 72.9, 70.0, 64.5, 57.7, 53.3, 51.7, 38.9, 22.7. HRMS (ESI-TOF) *m*/*z*: [M + Na]^+^ calcd for C_21_H_27_NO_8_SNa, 476.1350; found, 476.1368.

##### Methyl (Methyl 5-acetamido-4-*O*-(*tert*-butyldimethylsilyl)-3,5-dideoxy-8,9-*O*-isopropylidene-7-propargyloxy-d-*glycero*-α-d-*galacto*-2-nonulopyranosid)onate (**24**)

Compound **24** was synthesized starting from **7** (40.3 mg, 0.086 mmol) using the general procedure for *O*-alkylation (GP1 with 1.5 equiv of NaH and 5 equiv of propargyl bromide; TLC: petroleum ether/EtOAc (2:3, v/v); *R*_*f*_ = 0.61). The compound was purified using flash chromatography (petroleum ether/EtOAc, gradient 0–60%) to give **24** (22.1 mg, 57%) as a pale-yellow solid. ^1^H NMR (CDCl_3_, 600 MHz): δ 5.62 (d, 1H, *J* = 7.5 Hz), 4.54 (ABdq, 2H, *J* = 16.2, 2.4 Hz), 4.36–4.26 (m, 2H), 4.24 (dd, 1H, *J* = 10.8, 2.0 Hz), 4.16–4.09 (m, 3H), 3.82 (s, 3H), 3.32–3.24 (m, 1H), 3.31 (s, 3H), 2.57 (dd, 1H *J* = 12.8, 4.7 Hz), 2.49 (t, 1H, *J* = 2.3 Hz), 1.93 (s, 3H), 1.68 (*app*t, 1H, *J* = 12.3 Hz), 1.43 (s, 3H), 1.35 (s, 3H), 0.86 (s, 9H), 0.05 (s, 3H), 0.01 (s, 3H). ^13^C{^1^H} NMR (CDCl_3_, 150 MHz): δ 170.2, 168.4, 107.9, 99.4, 81.5, 79.1, 74.3, 74.2, 72.6, 66.1, 65.1, 59.9, 55.9, 52.6, 52.0, 41.8, 26.5, 25.8, 25.0, 24.0, 18.0, −4.5, −4.8. HRMS (ESI-TOF) *m*/*z*: [M + Na]^+^ calcd for C_25_H_43_NO_9_SiNa, 552.2599; found, 552.2614.

##### Methyl (Methyl 5-acetamido-4-*O*-(*tert*-butyldimethylsilyl)-3,5-dideoxy-8,9-*O*-isopropylidene-7-propargyloxy-d-*glycero*-α-d-*galacto*-2-nonulopyranosid)onate (**25**)

Compound **25** was synthesized starting from **8** (49.2 mg, 0.086 mmol) using the general procedure for *O*-alkylation (GP1 with 1.5 equiv of NaH and 5 equiv of propargyl bromide; TLC: petroleum ether/EtOAc (3:2, v/v); *R*_*f*_ = 0.24). The compound was purified using flash chromatography (petroleum ether/EtOAc, gradient 0–50%) to give **25** (37.1 mg, 71%) as a pale-yellow solid. ^1^H NMR (CD_3_OD, 600 MHz): δ 7.55 (dd, 2H, *J* = 8.3, 1.4 Hz), 7.44 (t, 1H, *J* = 7.4 Hz), 7.38 (t, 2H, *J* = 7.5 Hz), 4.40 (ABdq, 2H, *J* = 15.0, 2.5 Hz), 4.11 (ddd, 1H, *J* = 7.6, 6.4, 4.2 Hz), 4.03 (dd, 1H, *J* = 8.5, 7.7 Hz, 1H), 3.89 (dd, 1H, *J* = 8.5, 6.5 Hz), 3.80 (t, 1H, *J* = 10.7 Hz), 3.77 (d, 1H, *J* = 4.1 Hz), 3.59 (s, 3H), 3.66–3.57 (m, 1H), 3.48 (d, 1H, *J* = 10.6 Hz), 2.90 (t, 1H, *J* = 2.4 Hz), 2.67 (dd, 1H, *J* = 12.9, 4.6 Hz), 1.93 (s, 3H), 1.74 (dd, 1H, *J* = 12.9, 11.2 Hz), 1.38 (s, 3H), 1.25 (s, 3H), 0.87 (s, 9H), 0.07 (s, 3H), 0.04 (s, 3H). ^13^C{^1^H} NMR (CD_3_OD, 150 MHz): δ 173.0, 170.5, 138.0, 131.1, 130.5, 129.9, 109.3, 88.6, 81.0, 78.8, 77.3, 76.6, 76.2, 71.3, 67.1, 61.1, 53.3, 52.9, 42.7, 26.8, 26.1, 25.6, 23.3, 18.7, −4.4, −4.7. HRMS (ESI-TOF) *m*/*z*: [M + Na]^+^ calcd for C_30_H_45_NO_8_SSiNa, 630.2527; found, 630.2553.

##### Methyl (Methyl 5-acetamido-3,5-dideoxy-8,9-*O*-isopropylidene-d-*glycero*-α-d-*galacto*-2-nonulopyranosid)onate (**26**).^1^

Compound **26** was synthesized in 88% yield following the procedure described in ref (1). ^1^H NMR (CD_3_OD, 600 MHz): δ 4.25 (q, 1H, *J* = 6.3 Hz), 4.08 (dd, 1H, *J* = 8.3, 6.4 Hz), 4.00 (dd, 1H, *J* = 8.3, 6.5 Hz), 3.80 (s, 3H), 3.77 (d, 1H, *J* = 10.3 Hz), 3.63 (ddd, 1H, *J* = 12.0, 10.1, 4.7 Hz), 3.60 (d, 1H, *J* = 6.2 Hz), 3.55 (dd, 1H, *J* = 10.5, 1.5 Hz), 3.35 (s, 3H), 2.61 (dd, 1H, *J* = 12.8, 4.6 Hz), 1.99 (s, 3H), 1.67 (dd, 1H, *J* = 12.7, 11.9 Hz), 1.37 (s, 3H), 1.34 (s, 3H). ^13^C{^1^H} NMR (CD_3_OD, 150 MHz): δ 174.8, 170.1, 109.8, 100.6, 77.4, 75.4, 70.6, 68.6, 67.4, 53.8, 52.8, 52.1, 41.4, 27.1, 25.8, 22.7. HRMS (ESI-TOF) *m*/*z*: [M + Na]^+^ calcd for C_16_H_27_NO_9_Na, 400.1578; found, 400.1596.

##### Methyl (Phenyl 5-acetamido-3,5-dideoxy-8,9-*O*-isopropylidene-2-thio-d-*glycero*-α-d-*galacto*-2-nonulopyranosid)onate (**27**).^4^

Compound **27** was synthesized in 92% yield following the procedure described in ref (4). ^1^H NMR (CD_3_OD, 600 MHz): δ 7.58 (dd, 2H, *J* = 7.6, 1.6 Hz), 7.42 (t, 1H, *J* = 7.2 Hz), 7.36 (t, 2H, *J* = 7.6 Hz), 4.17 (q, 1H, *J* = 6.7 Hz), 3.97 (dd, 1H, *J* = 8.3, 6.2 Hz, 1H), 3.88 (dd, 1H, *J* = 8.4, 6.7 Hz), 3.79 (t, 1H, *J* = 10.3 Hz), 3.61 (ddd, 1H, *J* = 11.3, 10.1, 4.7 Hz), 3.51–3.47 (m, 1H), 3.48 (s, 3H), 3.24 (d, 1H, *J* = 10.7 Hz), 2.81 (dd, 1H, *J* = 12.7, 4.8 Hz), 1.97 (s, 3H), 1.77 (dd, 1H, *J* = 12.8, 11.3 Hz), 1.31 (s, 3H), 1.22 (s, 3H). ^13^C{^1^H} NMR (CD_3_OD, 150 MHz): δ 174.8, 170.5, 137.9, 130.9, 130.8, 129.7, 109.8, 88.6, 77.6, 76.7, 71.2., 69.0, 68.0, 53.5, 52.6, 42.0, 27.0, 25.7, 22.7. HRMS (ESI-TOF) *m*/*z*: [M + Na]^+^ calcd for C_21_H_29_NO_8_SNa, 478.1506; found, 478.1523.

##### Methyl (Methyl 5-acetamido-3,5-dideoxy-8,9-*O*-isopropylidene-4-propargyloxy-d-*glycero*-α-d-*galacto*-2-nonulopyranosid)onate (**28**)

Compound **28** was synthesized starting from **26** (32.6 mg, 0.086 mmol) using the general procedure for *O*-alkylation (GP1 with 1.1 equiv of NaH and 5 equiv of propargyl bromide; TLC: petroleum ether/EtOAc (1:4, v/v); *R*_*f*_ = 0.21). The compound was purified using flash chromatography (petroleum ether/EtOAc, gradient 30–90%) to give **28** (11 mg, 31%) as a white solid. ^1^H NMR (CD_3_OD, 600 MHz): δ 4.24 (q, 1H, *J* = 6.3 Hz), 4.21 (t, 2H, *J* = 2.2 Hz), 4.04 (ABdq, 2H, *J* = 8.3, 6.3 Hz), 3.88 (t, 1H, *J* = 10.3 Hz), 3.81 (s, 3H), 3.69 (ddd, 1H, *J* = 11.4, 10.0, 4.5 Hz), 3.65 (d, 1H, *J* = 10.7 Hz), 3.60 (d, 1H, *J* = 6.3 Hz), 3.35 (s, 3H), 2.88 (t, 1H, *J* = 2.4 Hz), 2.81 (dd, 1H, *J* = 12.7, 4.7 Hz), 1.97 (s, 3H), 1.61 (dd, 1H, *J* = 12.8, 11.6 Hz), 1.37 (s, 3H), 1.34 (s, 3H). ^13^C{^1^H} NMR (CD_3_OD, 150 MHz): δ 174.31, 170.0, 109.8, 100.6, 80.7, 77.4, 76.0, 75.8, 75.1, 70.4, 67.4, 57.5, 52.8, 52.1, 51.9, 38.4, 27.1, 25.8, 22.8. HRMS (ESI-TOF) *m*/*z*: [M + Na]^+^ calcd for C_19_H_29_NO_9_Na, 438.1735; found, 438.1749.

##### Methyl (Phenyl 5-acetamido-3,5-dideoxy-8,9-*O*-isopropylidene-4-propargyloxy-2-thio-d-*glycero*-α-d-*galacto*-2-nonulopyranosid)onate (**29**)

Compound **29** was synthesized starting from **27** (39.3 mg, 0.086 mmol) using the general procedure for *O*-alkylation (GP1 with 1.1 equiv of NaH and 5 equiv of propargyl bromide; TLC: petroleum ether/EtOAc (1:4, v/v); *R*_*f*_ = 0.29). The compound was purified using flash chromatography (petroleum ether/EtOAc, gradient 20–90%) to give **29** (20.9 mg, 49%) as a pale-yellow solid. ^1^H NMR (CD_3_OD, 600 MHz): δ 7.58 (dd, 2H, *J* = 8.3, 1.6 Hz), 7.42 (dd, 1H, *J* = 7.7,6.9 Hz), 7.36 (t, 2H, *J* = 7.6 Hz), 4.26–4.18 (m, 2H), 4.15 (q, 1H, *J* = 6.7 Hz, 1H), 3.97 (dd, 1H, *J* = 8.4, 6.3 Hz), 3.91–3.85 (m, 2H), 3.65 (dt, 1H, *J* = 10.7, 4.9 Hz), 3.50 (s, 3H), 3.52–3.48 (m, 1H), 3.45 (dd, 1H, *J* = 10.9, 1.5 Hz), 3.02 (dd, 1H, *J* = 12.7, 4.9 Hz), 2.87 (t, 1H, *J* = 2.5 Hz), 1.95 (s, 3H), 1.70 (dd, 1H, *J* = 12.7, 11.2 Hz), 1.31 (s, 3H), 1.22 (s, 3H). ^13^C{^1^H} NMR (CD_3_OD, 150 MHz): δ 174.3, 170.3, 137.9, 131.0, 130.7, 129.7, 109.9, 88.5, 80.7, 77.4, 76.7, 76.4, 76.1, 71.0, 68.0, 57.7, 52.6, 51.6, 39.1, 27.0, 25.7, 22.8. HRMS (ESI-TOF) *m*/*z*: [M + Na]^+^ calcd for C_24_H_31_NO_8_SNa, 516.1663; found, 516.1683.

##### Methyl (Methyl 5-acetamido-4-benzyloxy-3,5-dideoxy-d-*glycero*-α-d-*galacto*-2-nonulopyranosid)onate (**30**)

Compound **30** was synthesized starting from **6** (40 mg, 0.086 mmol) using the general procedure for *O*-alkylation (GP1 with 1.1 equiv of NaH and 5 equiv of benzyl bromide; TLC/EtOAc; *R*_*f*_ = 0.57) and the general procedure for deacetylation (GP2), successively. The compound was purified using flash chromatography (Tol/CH_3_OH, gradient 5–20%) to give **30** (22.6 mg, 64%) as a white solid. ^1^H NMR (CD_3_OD, 600 MHz): δ 7.47–7.21 (m, 5H), 4.58 (ABq, 2H, *J* = 11.9 Hz), 3.94 (t, 1H, *J* = 10.3 Hz), 3.81 (s, 3H), 3.87–3.79 (m, 2H), 3.68–3.62 (m, 2H), 3.57 (ddd, 1H, *J* = 11.7, 10.1, 4.6 Hz), 3.52 (dd, 1H, *J* = 9.0, 1.7 Hz), 3.34 (s, 3H), 2.78 (dd, 1H, *J* = 12.8, 4.6 Hz), 1.96 (s, 3H), 1.68 (dd, 1H, *J* = 12.8, 11.7 Hz). ^13^C{^1^H} NMR (CD_3_OD, 150 MHz): δ 174.6, 170.7, 139.6, 129.4, 128.9, 128.8, 100.4, 76.1, 74.6, 72.4, 72.2, 70.1, 64.7, 53.3, 52.0, 38.6, 22.7. HRMS (ESI-TOF) *m*/*z*: [M + Na]^+^ calcd for C_20_H_29_NO_9_Na, 450.1735; found, 450.1749.

##### Methyl (Methyl 5-acetamido-4-allyloxy-3,5-dideoxy-d-*glycero*-α-d-*galacto*-2-nonulopyranosid)onate (**31**)

Compound **31** was synthesized starting from **6** (40 mg, 0.086 mmol) using the general procedure for *O*-alkylation (GP1 with 1.1 equiv of NaH and 5 equiv of allyl bromide; TLC/EtOAc; *R*_*f*_ = 0.43) and the general procedure for deacetylation (GP2), successively. The compound was purified using flash chromatography (Tol/CH_3_OH, gradient 5–20%) to give **31** (17 mg, 52%) as a white solid. ^1^H NMR (CD_3_OD, 600 MHz): δ 5.89 (ddt, 1H, *J* = 17.3, 10.4, 5.5 Hz), 5.27 (qd, 1H, *J* = 17.2, 1.8 Hz), 5.16 (qd, 1H, *J* = 10.5, 1.4 Hz), 4.12 (ddt, 1H, *J* = 12.9, 5.5, 1.6 Hz), 3.99 (ddd, 1H, *J* = 12.8, 5.7, 1.6 Hz), 3.91–3.80 (m, 3H), 3.84 (s, 3H), 3.68–3.60 (m, 2H), 3.56–3.48 (m, 2H), 3.35 (s, 3H), 2.79 (dd, 1H, *J* = 12.8, 4.6 Hz), 1.98 (s, 3H), 1.64 (dd, 1H, *J* = 12.8, 11.7 Hz, 1H). ^13^C{^1^H} NMR (CD_3_OD, 150 MHz): δ 174.7, 170.7, 136.1, 117.2, 100.4, 75.9, 74.7, 72.4, 71.2, 70.2, 64.7, 53.3, 52.1, 52.0, 38.6, 22.7. HRMS (ESI-TOF) *m*/*z*: [M + Na]^+^ calcd for C_16_H_27_NO_9_Na, 400.1578; found, 400.1587.

##### Methyl (Methyl 5-acetamido-3,5-dideoxy-4-(2-methoxy-2-oxoethoxy)-d-*glycero*-α-d-*galacto*-2-nonulopyranosid)onate (**32**)

Compound **32** was synthesized starting from **6** (40 mg, 0.086 mmol) using the general procedure for *O*-alkylation (GP1 with 1.1 equiv of NaH and 5 equiv of methyl bromoacetate; TLC/EtOAc; *R*_*f*_ = 0.35) and the general procedure for deacetylation (GP2), successively. The compound was purified using flash chromatography (Tol/CH_3_OH, gradient 5–20%) to give **32** (28 mg, 79%) as a pale-yellow solid. ^1^H NMR (CD_3_OD, 600 MHz): δ 4.21 (ABq, 2H, *J* = 16.8 Hz), 3.89–3.81 (m, 3H), 3.84 (s, 3H), 3.75 (s, 3H), 3.67–3.56 (m, 3H), 3.52 (dd, 1H, *J* = 8.9, 1.6 Hz), 3.35 (s, 3H), 2.82 (dd, 1H, *J* = 12.9, 4.7 Hz), 2.01 (s, 3H), 1.69 (dd, 1H, *J* = 12.9, 11.7 Hz). ^13^C{^1^H} NMR (CD_3_OD, 150 MHz): δ 175.1, 173.0, 170.7, 100.3, 77.3, 74.8, 72.3, 70.2, 67.2, 64.7, 53.4, 52.4, 52.3, 52.0, 38.3, 22.8. HRMS (ESI-TOF) *m*/*z*: [M + Na]^+^ calcd for C_16_H_27_NO_11_Na, 432.1476; found, 432.1491.

##### Methyl (Methyl 5-acetamido-3,5-dideoxy-4-(hex-5-yn-1-yloxy)-d-*glycero*-α-d-*galacto*-2-nonulopyranosid)onate (**33**)

Compound **33** was synthesized starting from **6** (40 mg, 0.086 mmol) using the general procedure for *O*-alkylation (GP1 with 3 equiv of NaH, 5 equiv of 6-iodo-1-hexyne and DMF as solvent) and the general procedure for deacetylation (GP2; TLC: Tol/CH_3_OH (4:1, v/v); *R*_*f*_ = 0.37), successively. The compound was purified using flash chromatography (Tol/CH_3_OH, gradient 5–20%) to give **33** (14.4 mg, 40%) as a pale-yellow solid. ^1^H NMR (CD_3_OD, 600 MHz): δ 3.90–3.81 (m, 3H), 3.85 (s, 3H), 3.68–3.60 (m, 3H), 3.51 (dd, 1H, *J* = 9.0, 1.7 Hz), 3.48–3.38 (m, 2H), 3.03 (s, 3H), 2.78 (dd, 1H, *J* = 12.8, 4.6 Hz), 2.23–2.15 (m, 3H), 1.99 (s, 3H), 1.70–1.53 (m, 5H). ^13^C{^1^H} NMR (CD_3_OD, 150 MHz): δ 174.7, 170.8, 100.4, 84.9, 76.5, 74.7, 72.4, 70.2, 69.7, 64.8, 53.3, 52.1, 52.0, 38.5, 30.1, 26.5, 22.7, 18.8. HRMS (ESI-TOF) *m*/*z*: [M + Na]^+^ calcd for C_19_H_31_NO_9_Na, 440.1891; found, 440.1904.

##### Methyl (Methyl 5-acetamido-3,5-dideoxy-4-(pent-4-en-1-yloxy)-d-*glycero*-α-d-*galacto*-2-nonulopyranosid)onate (**34**)

Compound **34** was synthesized starting from **6** (40 mg, 0.086 mmol) using the general procedure for *O*-alkylation (GP1 with 2 equiv of NaH, 5 equiv of 5-bromo-1-pentene and DMF as solvent) and the general procedure for deacetylation (GP2; TLC: Tol/CH_3_OH (4:1, v/v); *R*_*f*_ = 0.33). The compound was purified using flash chromatography (Tol/CH_3_OH, gradient 5–20%) to give **34** (6 mg, 17%) as a pale-yellow solid. ^1^H NMR (CD_3_OD, 600 MHz): δ 5.84 (ddt, 1H, *J* = 17.1, 10.3, 6.7 Hz), 5.03 (qd, 1H, *J* = 17.1, 1.8 Hz), 4.97 (dd, 1H, *J* = 10.2, 2.1 Hz), 3.90–3.83 (m, 3H), 3.87 (s, 3H), 3.69–3.61 (m, 3H), 3.53 (dd, 1H, *J* = 8.9, 1.8 Hz), 3.49–3.40 (m, 2H), 3.37 (s, 3H), 2.78 (dd, 1H, *J* = 12.8, 4.6 Hz), 2.16–2.10 (m, 2H), 1.99 (s, 3H), 1.69–1.60 (m, 3H). ^13^C{^1^H} NMR (CD_3_OD, 150 MHz): δ 174.6, 170.8, 139.4, 115.3, 100.4, 76.5, 74.7, 72.4, 70.2, 69.6, 64.8, 53.3, 52.1, 52.0, 38.5, 31.3, 30.4, 22.7. HRMS (ESI-TOF) *m*/*z*: [M + Na]^+^ calcd for C_18_H_31_NO_9_Na, 428.1891; found, 428.1904.

##### Methyl (Methyl 5-acetamido-3,5-dideoxy-4-ethoxy-d-*glycero*-α-d-*galacto*-2-nonulopyranosid)onate (**35**)

Compound **35** was synthesized starting from **6** (40 mg, 0.086 mmol) using the general procedure for *O*-alkylation (GP1 with 1.1 equiv of NaH and 5 equiv of ethyl tosylate; TLC/EtOAc; *R*_*f*_ = 0.38) and the general procedure for deacetylation (GP2). The compound was purified using flash chromatography (Tol/CH_3_OH, gradient 5–30%) to give **35** (2.5 mg, 8%) as a pale-yellow oil. After flash chromatography and reverse-phase HPLC purification (H_2_O/acetonitrile w. 0.005% formic acid, gradient 5–65% over 25 min), the product still contained 20% of tosyl-containing byproducts. ^1^H NMR (CD_3_OD, 600 MHz): δ 3.88–3.79 (m, 3H), 3.85 (s, 3H), 3.69–3.62 (m, 2H), 3.60 (dd, 1H, *J* = 10.8, 1.7 Hz), 3.53–3.43 (m, 3H), 3.35 (s, 3H), 2.77 (dd, 1H, *J* = 12.8, 4.6 Hz), 1.98 (s, 3H), 1.62 (dd, 1H, *J* = 12.9, 11.7 Hz), 1.16 (t, 3H, *J* = 7.1 Hz). ^13^C{^1^H} NMR (CD_3_OD, 150 MHz): δ 174.8, 170.8, 100.4, 76.2, 74.8, 72.4, 70.2, 65.7, 64.7, 53.3, 52.2, 52.0, 38.6, 22.6, 15.9. HRMS (ESI-TOF) *m*/*z*: [M + Na]^+^ calcd for C_15_H_27_NO_9_Na, 388.1578; found, 388.1594.

##### Methyl (Phenyl 5-acetamido-7,8,9-tri-*O*-acetyl-3,5-dideoxy-4-propargyloxy-2-thio-d-*glycero*-α-d-*galacto*-2-nonulopyranosid)onate (**37**)

Compound **37** was isolated during the synthesis of **23**. The compound was purified using flash chromatography (*n*-hept/EtOAc, gradient). ^1^H NMR (CDCl_3_, 400 MHz): δ 7.52–7.47 (m, 2H), 7.40–7.28 (m, 3H), 5.43 (d, 1H, *J* = 8.8 Hz), 5.30–5.22 (m, 2H), 4.38 (dd, 1H, *J* = 12.5, 2.1 Hz), 4.23 (dd, 1H, *J* = 12.3, 4.8 Hz), 4.21 (dd, 1H, *J* = 16.2, 2.2 Hz), 4.15–4.04 (m, 2H), 3.86 (td, 1H, *J* = 10.8, 4.2 Hz), 3.54 (s, 3H), 3.39 (*app*q, 1H, *J* = 9.7 Hz), 2.97 (dd, 1H, *J* = 12.9, 4.6 Hz), 2.42 (t, 1H, *J* = 2.4 Hz), 2.13 (s, 3H), 2.04 (s, 3H), 2.02 (s, 3H), 1.93 (s, 3H), 1.74 (dd, 1H, *J* = 12.9, 11.2 Hz). ^13^C{^1^H} NMR (CDCl_3_, 100 MHz): δ 171.3, 170.7, 170.7, 170.6, 170.1, 168.2, 136.5, 129.9, 129.0, 128.9, 87.7, 79.7, 74.8, 73.8, 70.0, 68.3, 62.2, 56.7, 52.7, 51.3, 38.4, 23.8, 21.1, 21.0, 20.9. HRMS (ESI-TOF) *m*/*z*: [M + Na]^+^ calcd for C_27_H_33_NO_11_SNa, 602.1666; found, 602.1667.

##### Methyl 5-Acetamido-7,8,9-tri-*O*-acetyl-2,6-anhydro-3,5-dideoxy-4-propargyloxy-d-*glycero*-α-d-*galacto*-non-2-enonate (**38**)

Compound **37** (19 mg, 0.033 mmol) was dissolved in dry dichloromethane (630 μL) in the presence of molecular sieves (3 Å, 90 mg). The mixture was stirred at room temperature and under a nitrogen atmosphere for 16 h. At room temperature, *N*-iodosuccinimide (14.8 mg, 0.066 mmol, 2 equiv) and triflic acid (1 μL, 0.007 mmol, 0.2 equiv) were added, and the reaction was allowed to perform for 30 min. After completion, the mixture was diluted with dichloromethane (5 mL) and washed with a 20% solution of sodium thiosulfate (1 mL) and brine (1 mL) twice, successively. The organic layer was dried over anhydrous sodium sulfate, filtered, and concentrated to dryness. The compound was purified using flash chromatography (*n*-hept/acetone, gradient 10–50%) to give **37** (13.4 mg, 87%). ^1^H NMR (CDCl_3_, 400 MHz): δ 6.16 (d, 1H, *J* = 3.7 Hz), 5.63 (d, 1H, *J* = 8.4 Hz), 5.53 (dd, 1H, *J* = 5.1, 4.8 Hz), 5.40 (dt, 1H, *J* = 8.1, 3.5 Hz), 4.50 (dd, 1H, *J* = 12.1, 3.6 Hz), 4.48 (dd, 1H, *J* = 7.2, 4.7 Hz), 4.37 (dd, 1H, *J* = 5.3, 3.8 Hz), 4.33 (dd, 1H, *J* = 16.1, 2.3 Hz), 4.26 (dd, 1H, *J* = 16.1, 2.4 Hz), 4.22–4.13 (m, 2H), 3.80 (s, 3H), 2.46 (t, 1H, *J* = 2.3 Hz), 2.11 (s, 3H), 2.04 (s, 3H), 2.04 (s, 3H), 1.98 (s, 3H). ^13^C{^1^H} NMR (CDCl_3_, 100 MHz): δ 170.7, 170.2, 170.2, 169.9, 162.1, 144.1, 108.5, 79.4, 76.0, 75.3, 70.6, 69.9, 68.3, 62.0, 56.5, 52.7, 47.8, 23.5, 21.0, 20.9, 20.8. HRMS (ESI-TOF) *m*/*z*: [M + Na]^+^ calcd for C_21_H_27_NO_11_Na, 492.1476; found, 492.1472.

## Supporting Information Available

LC–MS total ion current chromatograms and ^1^H and ^13^C NMR spectra of compounds (PDF)
FAIR data, including the primary NMR FID files, for compounds **6**–**8**, **11**–**18**, **20**, **22**–**35**, **37**, and **38** (ZIP)
