# Supplementary material for: Synthesis of 4-O-Alkylated N-Acetylneuraminic Acid Derivatives
Source: J Org Chem. 2021 Jun 17;86(13):9145–54. doi: 10.1021/acs.joc.1c00235 (PMC8279483; doi:10.1021/acs.joc.1c00235)
Supplement: Supplementary file 1 — jo1c00235_si_001.pdf [file jo1c00235_si_001.pdf]

Supplementary Information for:

Synthesis of 4-*O*-alkylated *N*-acetylneuraminic acid derivatives

Emil Johansson‡, Rémi Caraballo‡, and Mikael Elofsson*
‡ Authors contributed equally.
*Professor Mikael Elofsson, Department of Chemistry, Umeå University, SE90187 Umeå, Sweden; orcid.org/0000-0002-3219-4669;

E-mail: mikael.elofsson@umu.se

This PDF file includes:

Supplementary Figures (S1 and S2)
^{1}H-NMR and ^{13}C-NMR spectra

Supplementary Figures

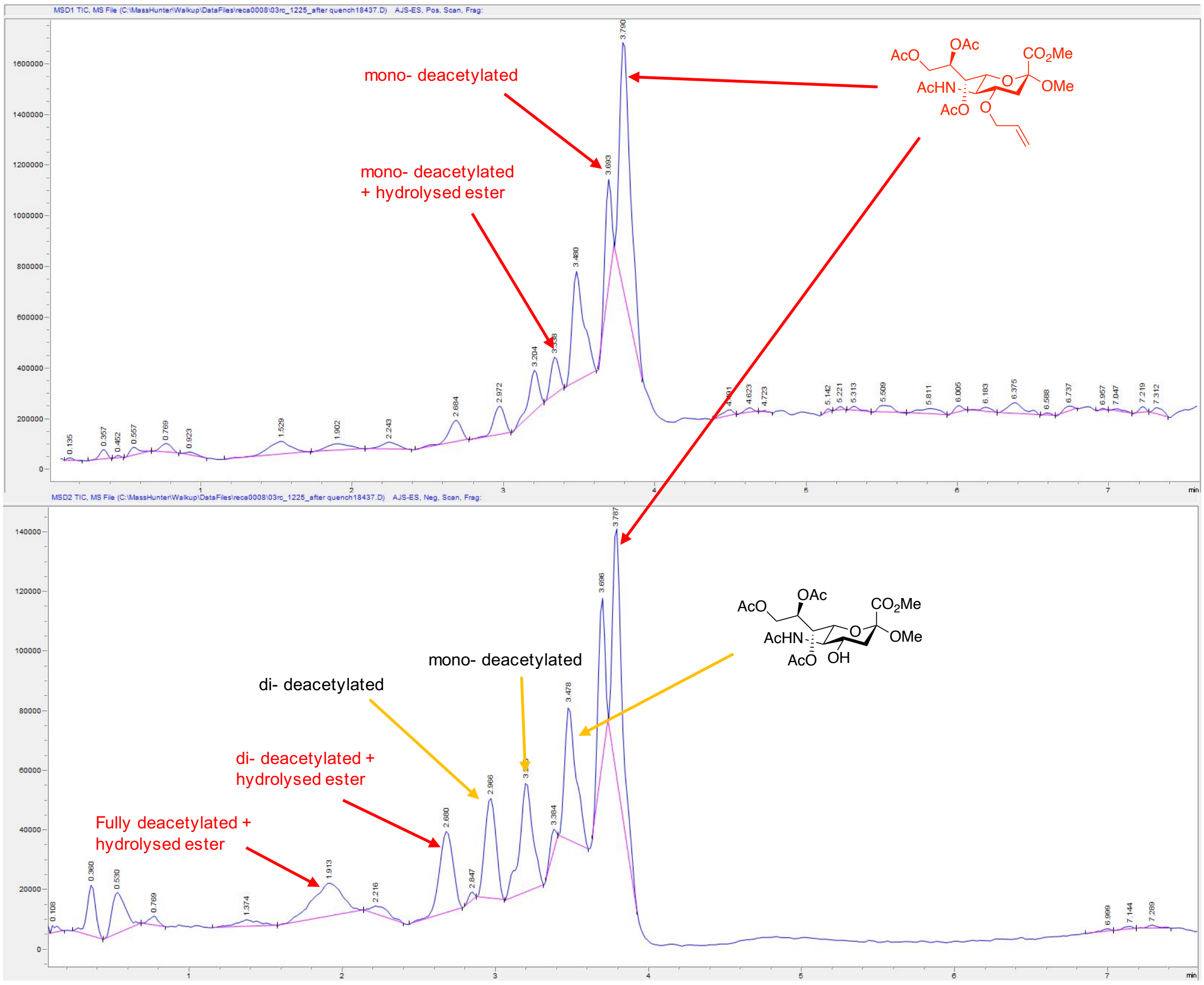

Figure S1. LC-MS total ion current chromatograms on positive (top) and negative (bottom) ionization modes of the *O*-alkylation reaction (after quenching) between compound **6** and allyl bromide (5 equiv.) in THF and in the presence of 1.1 equiv. of NaH. The alkylated product and side products resulting from deacetylation and ester hydrolysis of the alkylated product are highlighted in red. The starting material (**6**) and side products resulting from deacetylation and ester hydrolysis of **6** are highlighted in black.

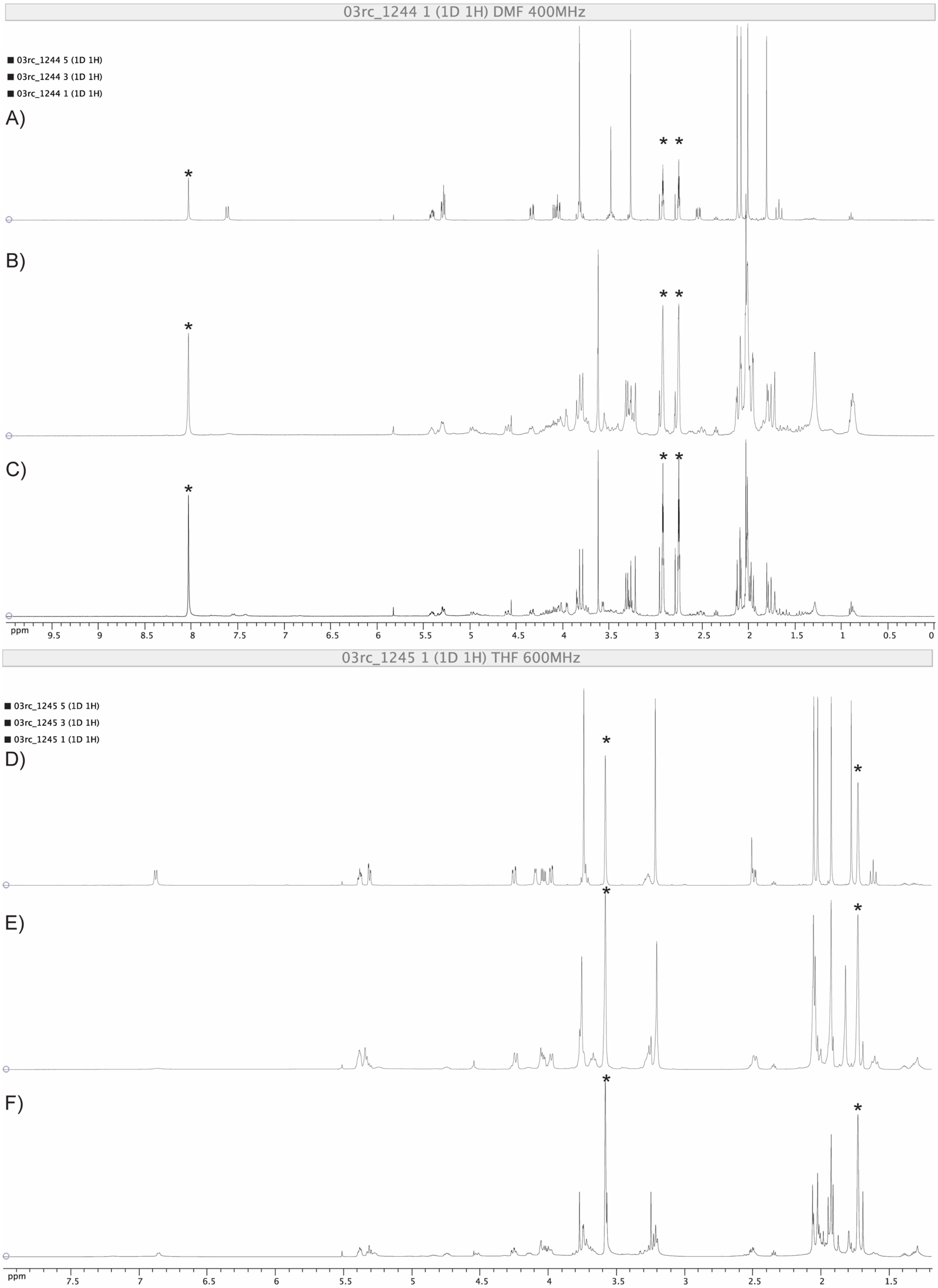

Figure S2. 1H-NMR of **6** in DMF-d_7 (A-C) and THF-d_8 (D-F). A and D) reference at t=0. B and E) 10 min post-addition of NaH. C and F) 1 h post addition of NaH.

^{1}H-NMR and ^{13}C-NMR

Compound **7**

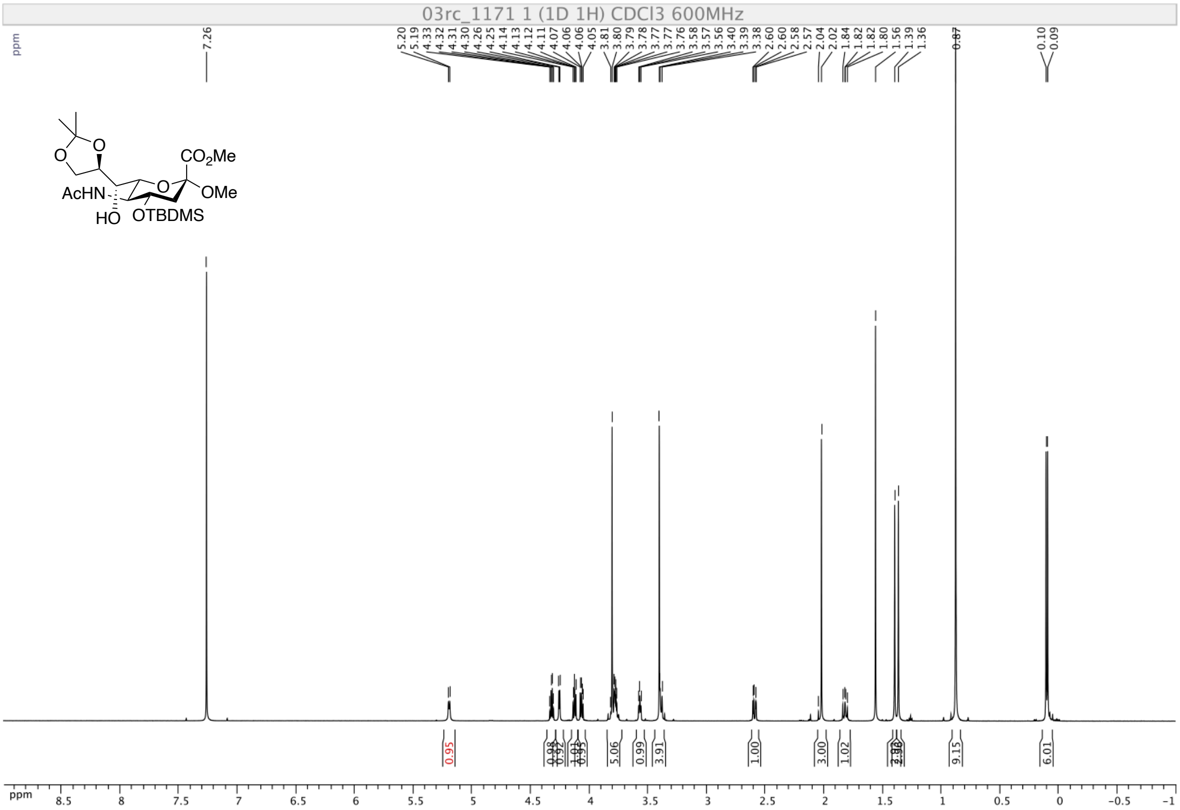

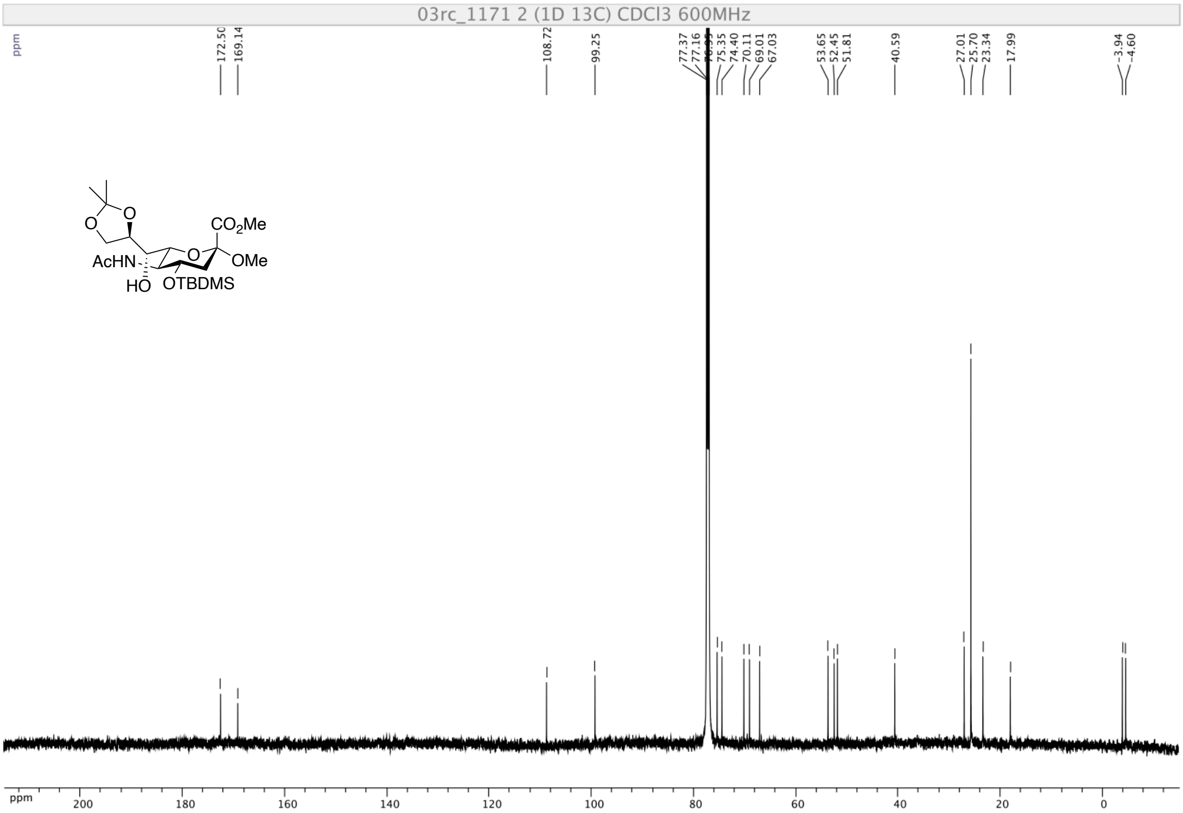

Compound **8**

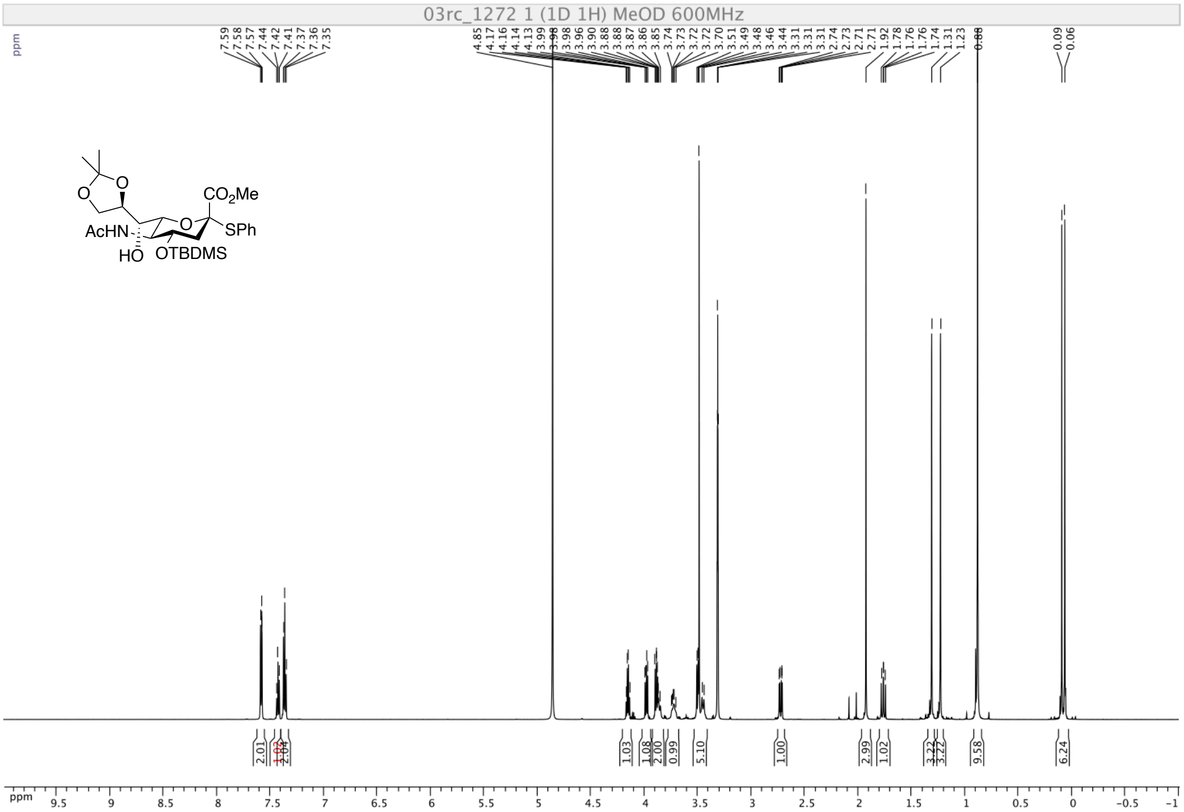
03rc_1272 1 (1D 1H) MeOD 600MHz
CO2Me
AcHN
SPh
HO
OTBDMS
2.01
2.04
1.03
1.08
2.00
0.99
5.10
1.00
2.99
1.02
3.22
3.27
9.58
6.24
ppm

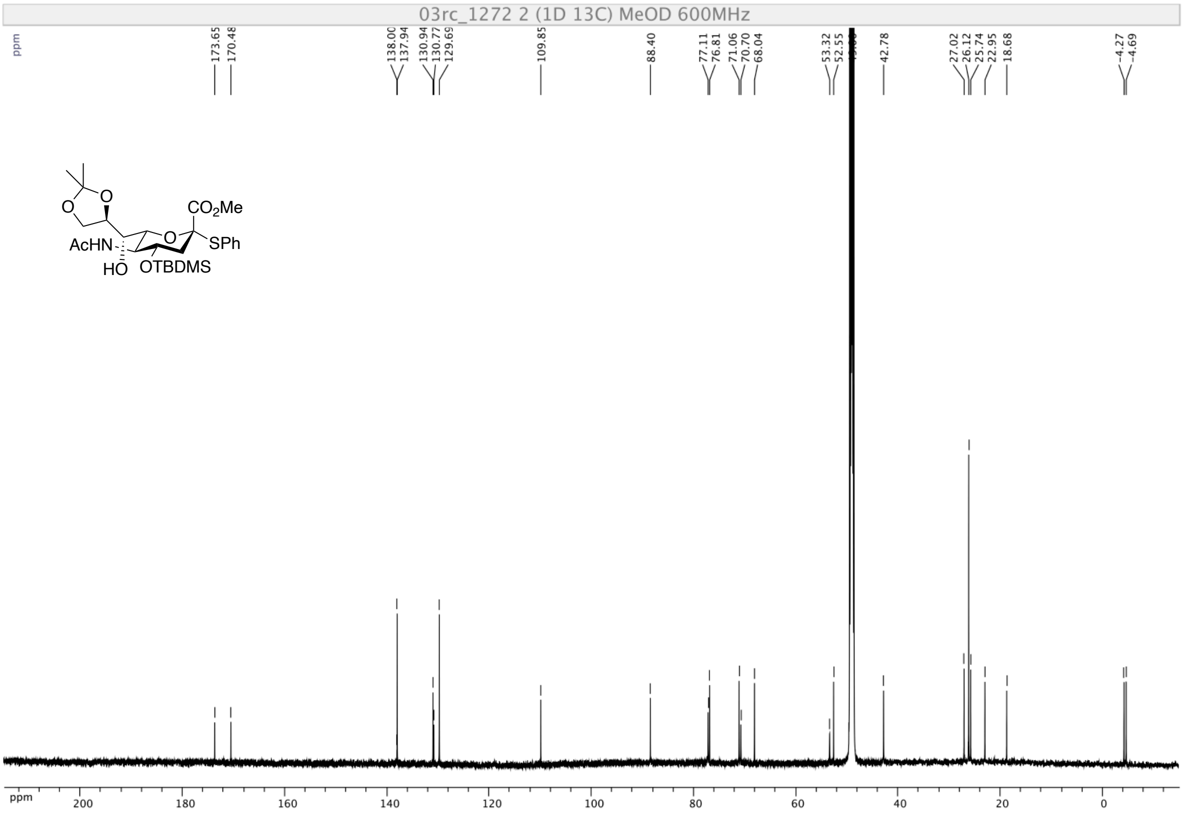
03rc_1272 2 (1D 13C) MeOD 600MHz
173.65
170.48
138.00
137.94
130.94
130.77
129.69
109.85
88.40
77.11
76.81
71.06
70.70
68.04
53.32
52.55
42.78
27.02
26.12
25.74
22.95
18.68
-4.27
-4.69
CO2Me
AcHN
SPh
HO
OTBDMS
ppm

Compound **11**

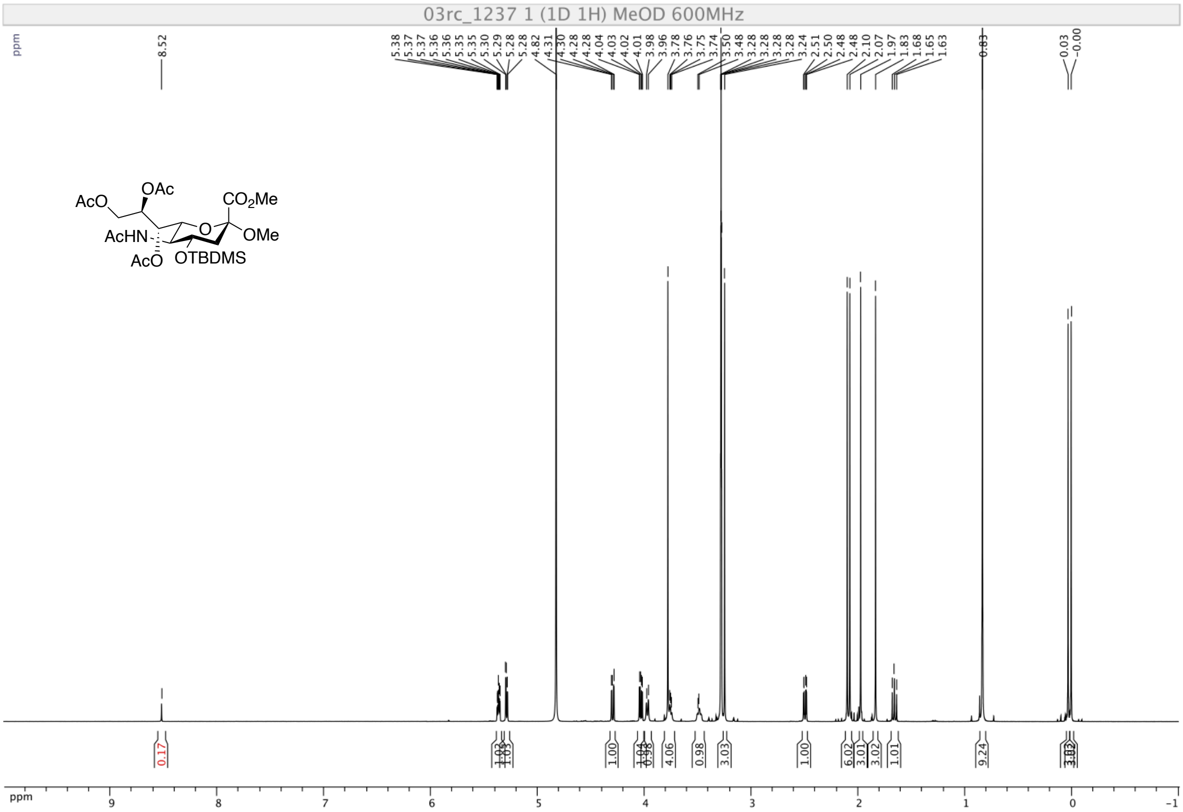
03rc_1237 1 (1D 1H) MeOD 600MHz
8.52
5.38 5.37 5.37 5.36 5.36 5.35 5.35 5.30 5.29 5.28 5.28 4.82 4.31 4.30 4.28 4.28 4.04 4.03 4.02 4.01 3.98 3.96 3.78 3.76 3.75 3.74 3.50 3.48 3.28 3.28 3.28 3.28 3.28 3.24 2.51 2.50 2.48 2.48 2.10 2.07 1.97 1.83 1.68 1.65 1.63
0.83
0.03 -0.00
AcO
OAc
CO2Me
AcHN
O
OMe
AcO
OTBDMS
0.17
1.00
4.06
0.98
3.03
1.00
6.02
3.01
3.02
1.01
9.24
ppm

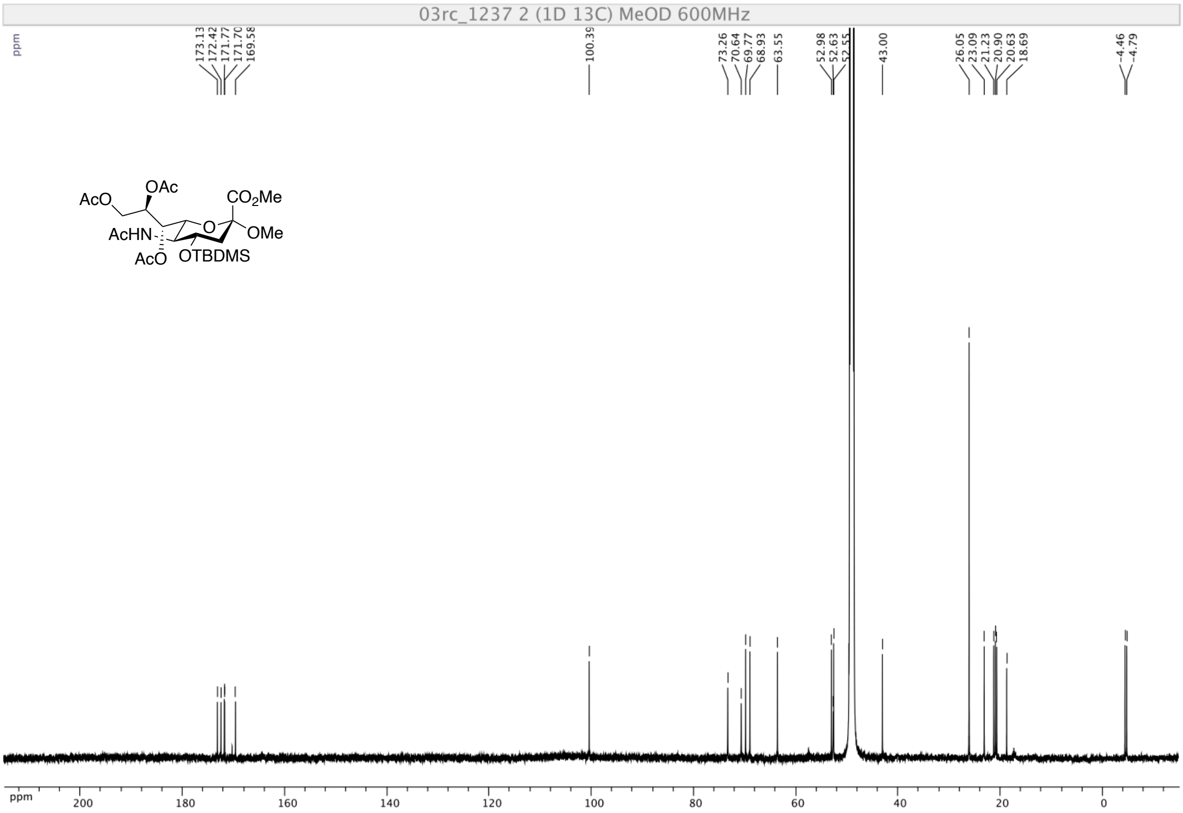
03rc_1237 2 (1D 13C) MeOD 600MHz
173.13 172.42 171.77 171.70 169.58
100.39
73.26 70.64 69.77 68.93 63.55 52.98 52.63 52.55
43.00
26.05 23.09 21.23 20.90 20.63 18.69
-4.46 -4.79
AcO
OAc
CO2Me
AcHN
O
OMe
AcO
OTBDMS
ppm

Compound **12**

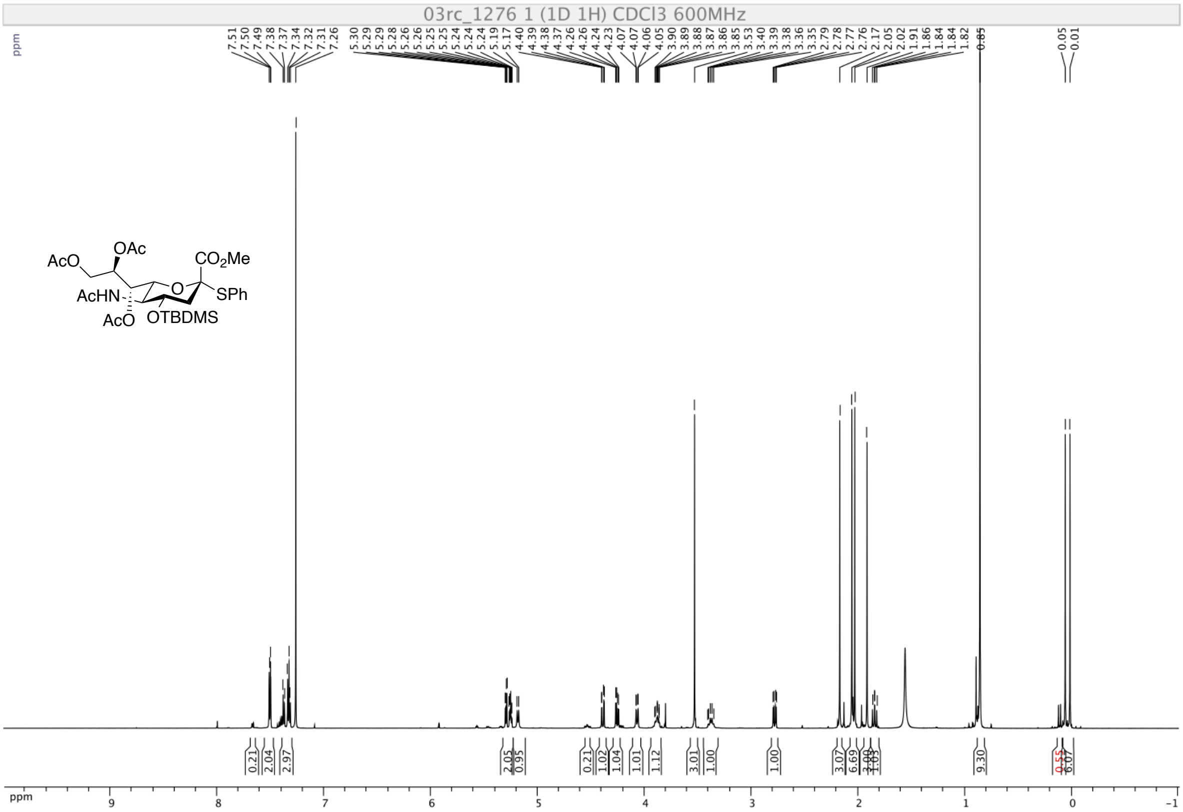
03rc_1276 1 (1D 1H) CDCl3 600MHz
AcO OAc CO2Me AcHN O SPh AcO OTBDMS
ppm

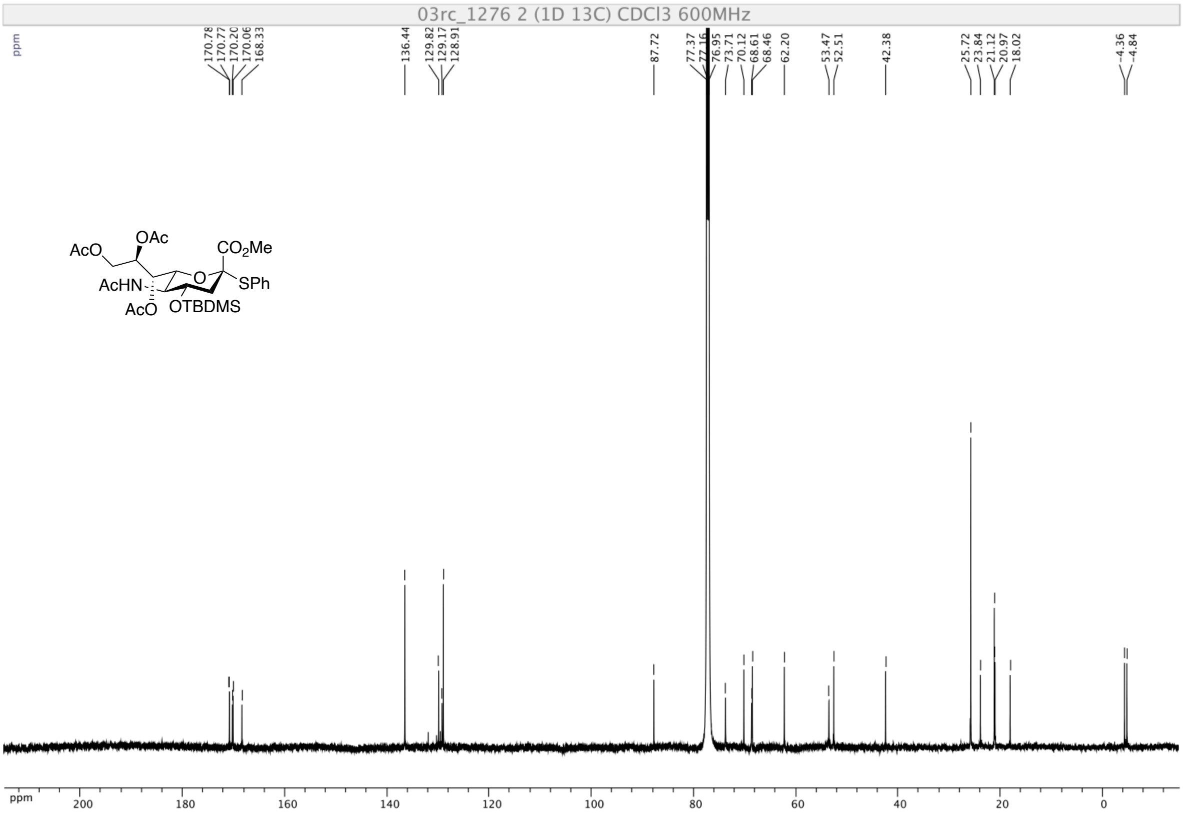
03rc_1276 2 (1D 13C) CDCl3 600MHz
AcO OAc CO2Me AcHN O SPh AcO OTBDMS
ppm

Compound **6**

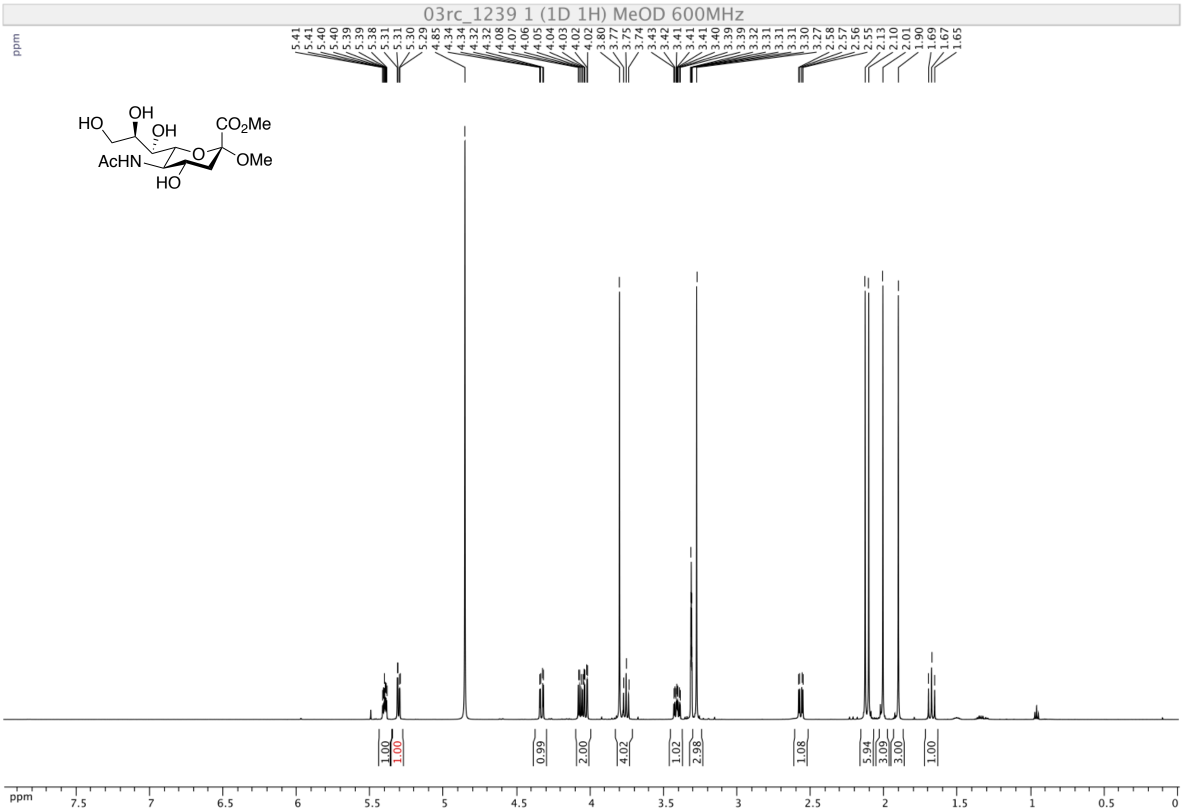
03rc_1239 1 (1D 1H) MeOD 600MHz
HO
OH
OH
CO_2Me
O
OMe
AcHN
HO
5.41 5.41 5.40 5.40 5.39 5.39 5.38 5.31 5.31 5.30 5.29 4.85 4.34 4.34 4.32 4.32 4.08 4.07 4.06 4.05 4.04 4.03 4.02 4.02 3.80 3.77 3.75 3.74 3.43 3.42 3.41 3.41 3.41 3.40 3.39 3.39 3.32 3.31 3.31 3.31 3.30 3.27 2.58 2.57 2.56 2.55 2.13 2.10 2.01 1.90 1.69 1.67 1.65
1.00 1.00 0.99 2.00 4.02 1.02 2.98 1.08 5.94 3.09 3.00 1.00
ppm 7.5 7 6.5 6 5.5 5 4.5 4 3.5 3 2.5 2 1.5 1 0.5 0

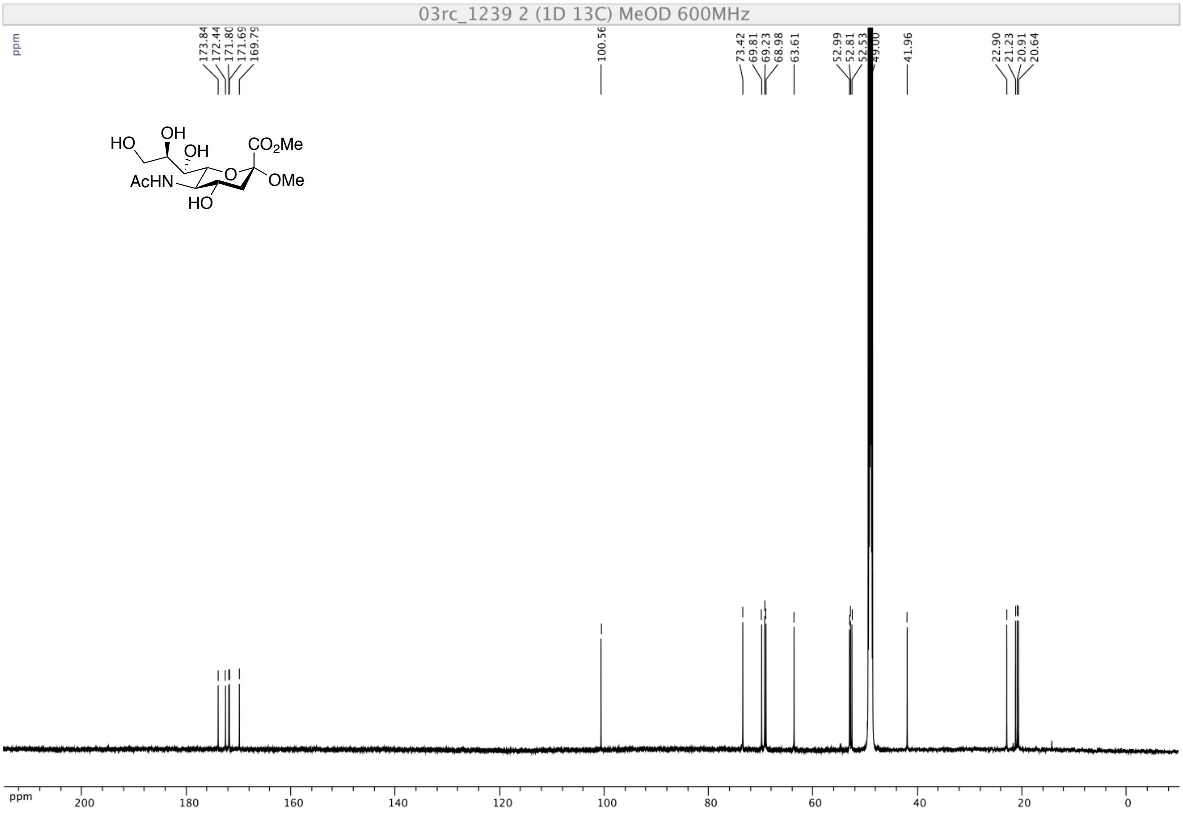
03rc_1239 2 (1D 13C) MeOD 600MHz
HO
OH
OH
CO_2Me
O
OMe
AcHN
HO
173.84 172.44 171.80 171.69 169.79 100.56 73.42 69.81 69.23 68.98 63.61 52.99 52.81 52.53 49.00 41.96 22.90 21.23 20.91 20.64
ppm 200 180 160 140 120 100 80 60 40 20 0

Compound **13**

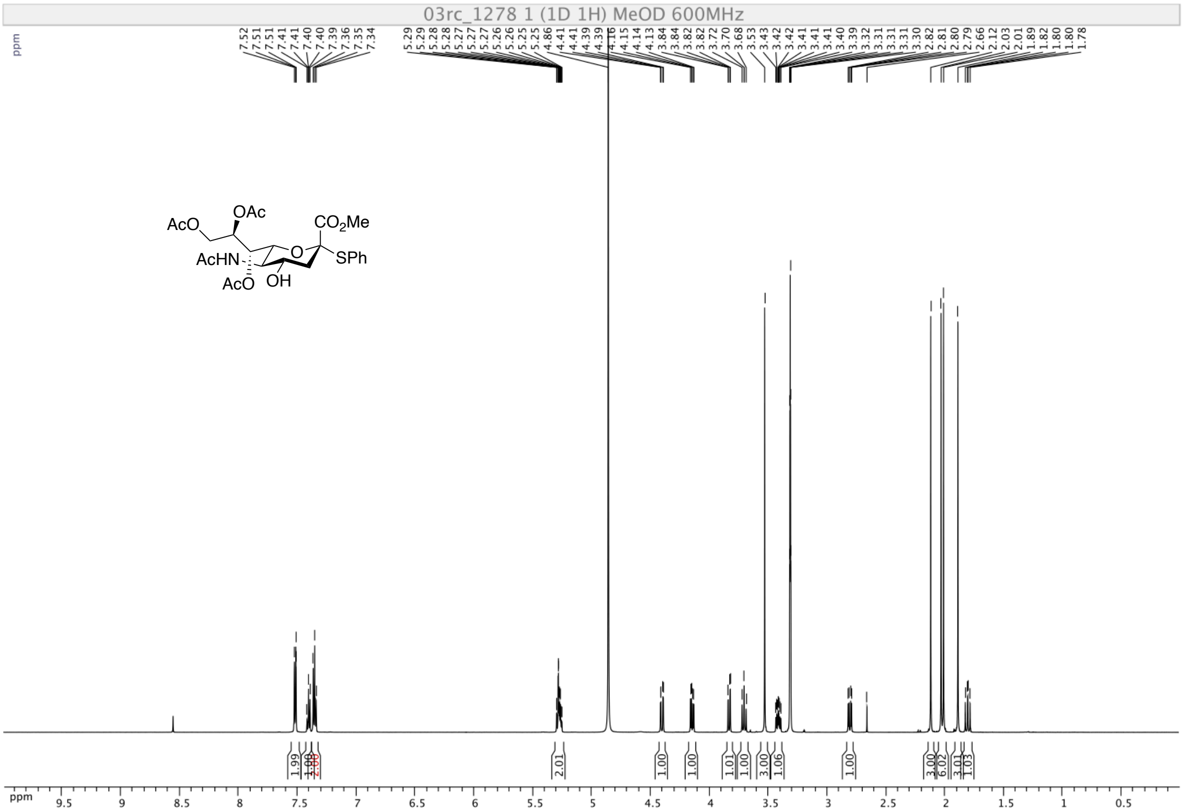
03rc_1278 1 (1D 1H) MeOD 600MHz
ppm
AcO
OAc
CO2Me
AcHN
O
SPh
AcO
OH

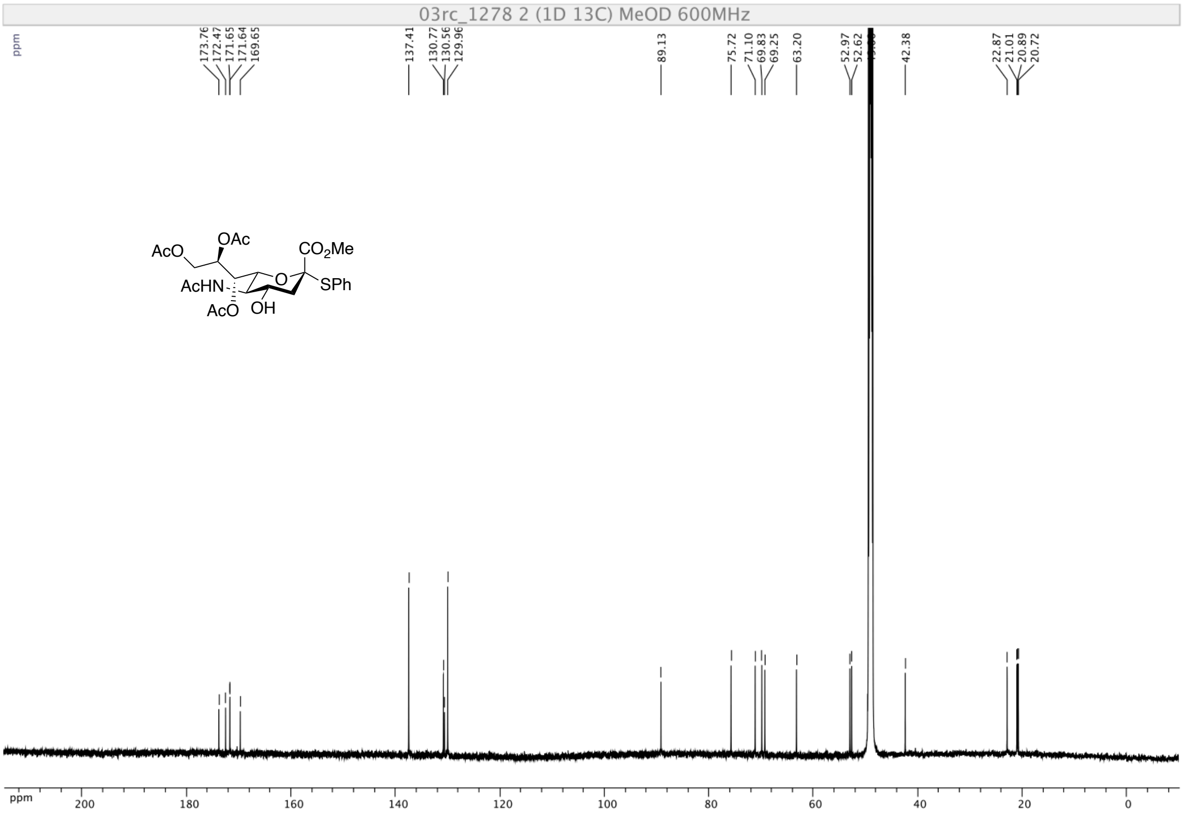
03rc_1278 2 (1D 13C) MeOD 600MHz
ppm
AcO
OAc
CO2Me
AcHN
O
SPh
AcO
OH

Compound **14**

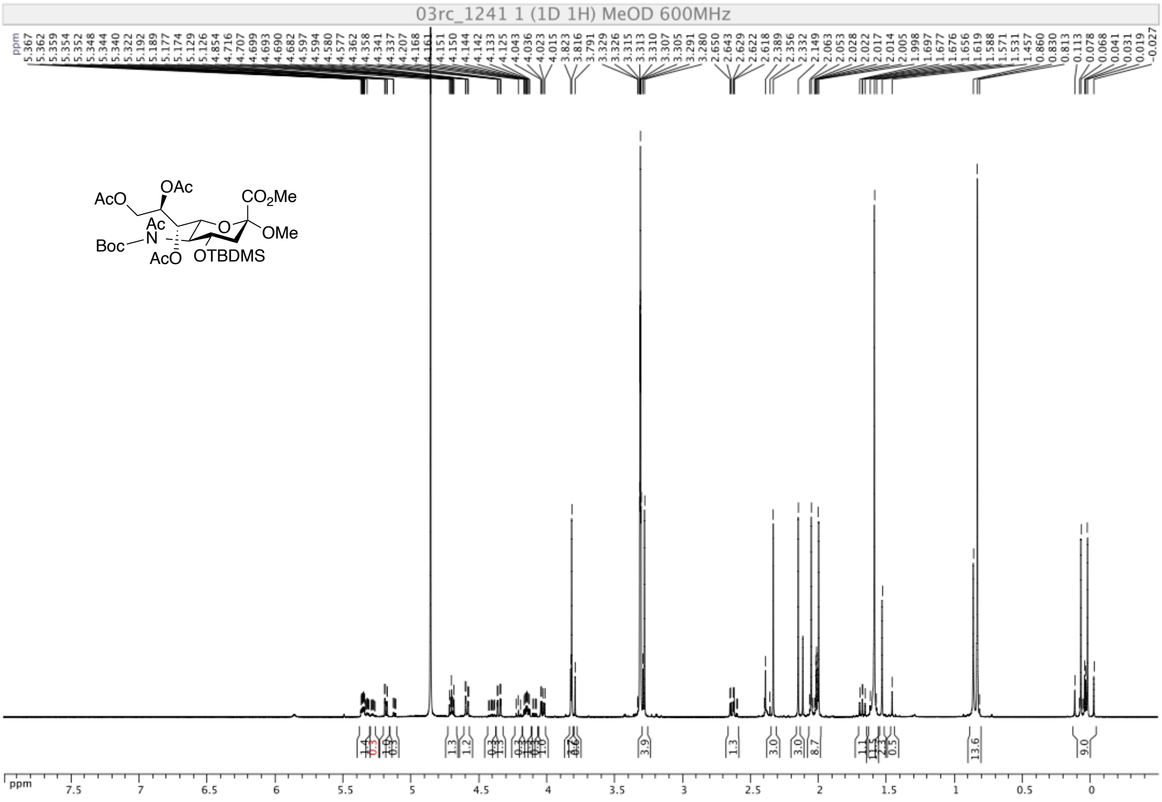
03rc_1241 1 (1D 1H) MeOD 600MHz
ppm
5.367 5.362 5.359 5.354 5.352 5.348 5.344 5.340 5.322 5.192 5.189 5.177 5.174 5.129 5.126 4.854 4.716 4.707 4.699 4.693 4.690 4.682 4.597 4.594 4.580 4.577 4.362 4.358 4.341 4.337 4.207 4.168 4.161 4.151 4.150 4.144 4.142 4.133 4.125 4.043 4.036 4.023 4.015 3.823 3.816 3.791 3.329 3.326 3.315 3.313 3.310 3.307 3.305 3.291 3.280 2.650 2.643 2.629 2.622 2.618 2.389 2.356 2.332 2.149 2.063 2.053 2.028 2.022 2.017 2.014 2.005 1.998 1.697 1.677 1.676 1.656 1.619 1.588 1.571 1.531 1.457 0.860 0.830 0.813 0.113 0.078 0.068 0.041 0.031 0.019 -0.027
AcO
OAc
CO2Me
Ac
N
O
OMe
Boc
AcO
OTBDMS

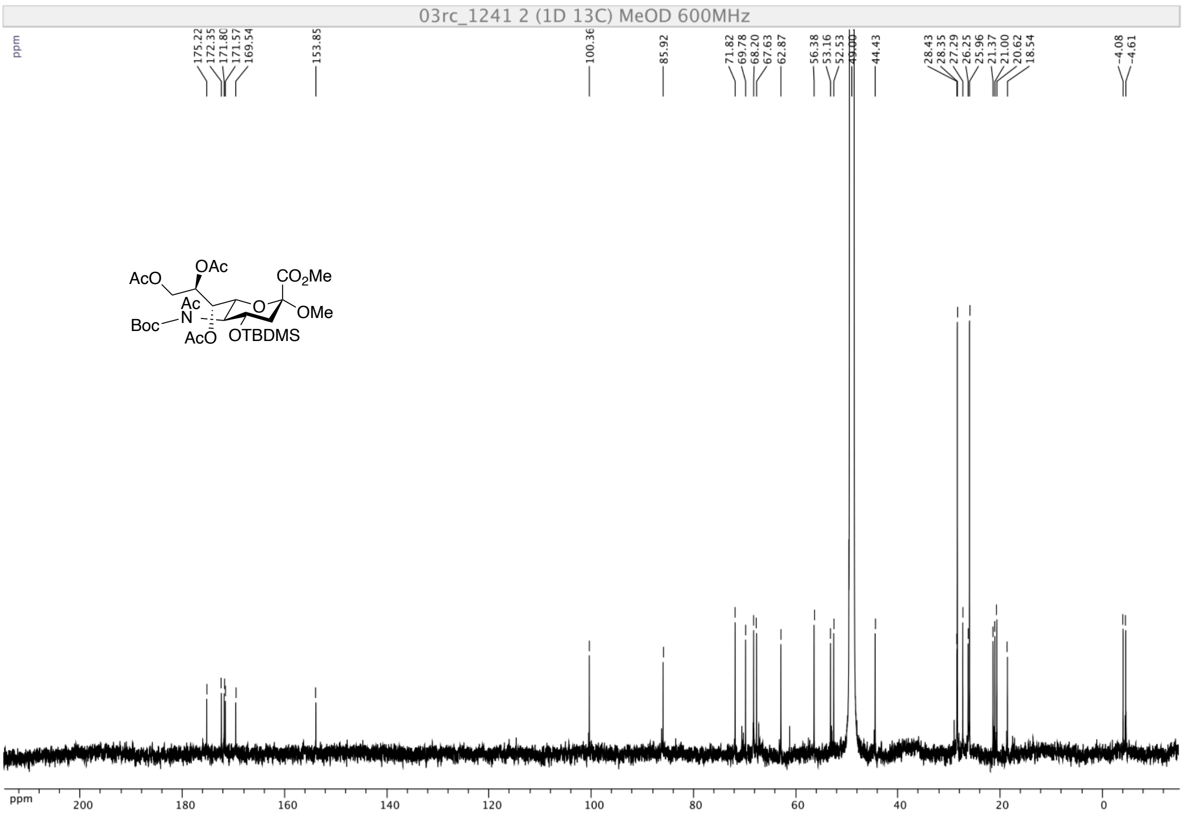
03rc_1241 2 (1D 13C) MeOD 600MHz
ppm
175.22 172.35 171.80 171.57 169.54 153.85 100.36 85.92 71.82 69.78 68.20 67.63 62.87 56.38 53.16 52.53 49.00 44.43 28.43 28.35 27.29 26.25 25.96 21.37 21.00 20.62 18.54 -4.08 -4.61
AcO
OAc
CO2Me
Ac
N
O
OMe
Boc
AcO
OTBDMS

Compound **15**

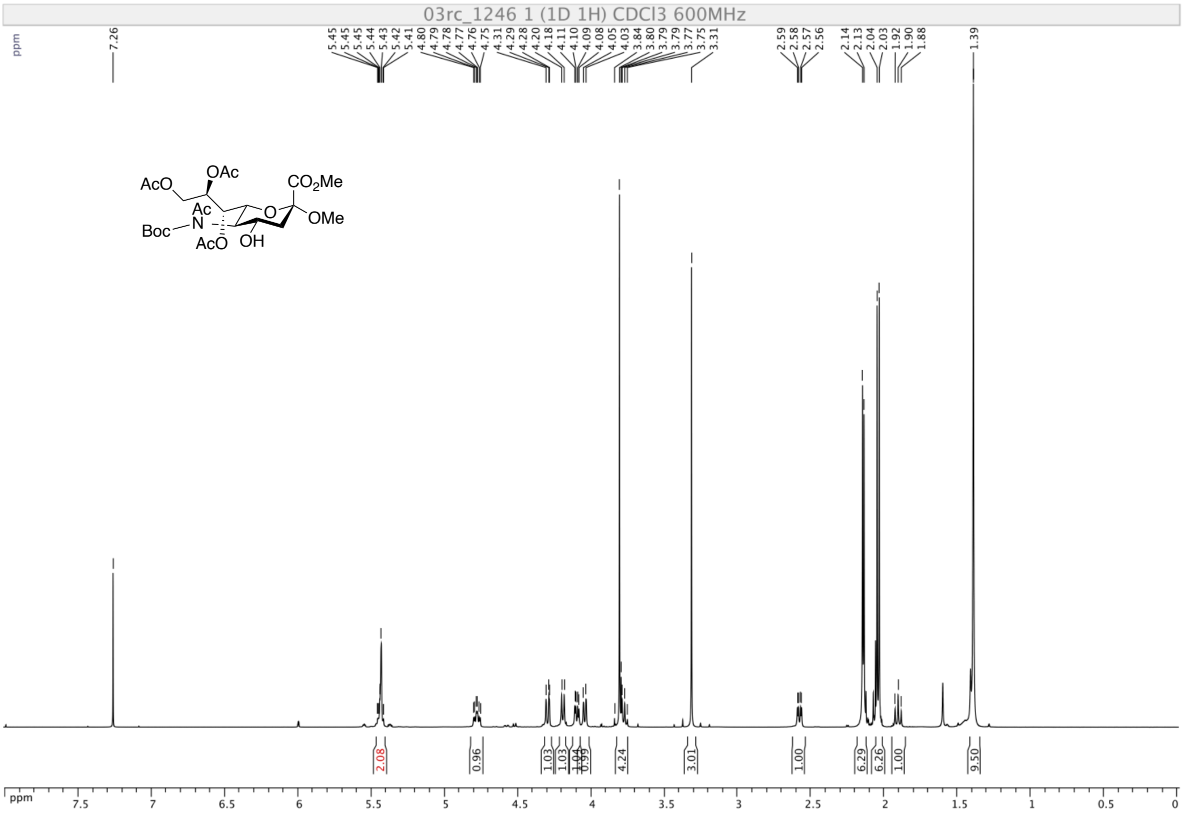
03rc_1246 1 (1D 1H) CDCl3 600MHz
AcO
OAc
CO2Me
Ac
N
Boc
AcO
OH
O
OMe
ppm

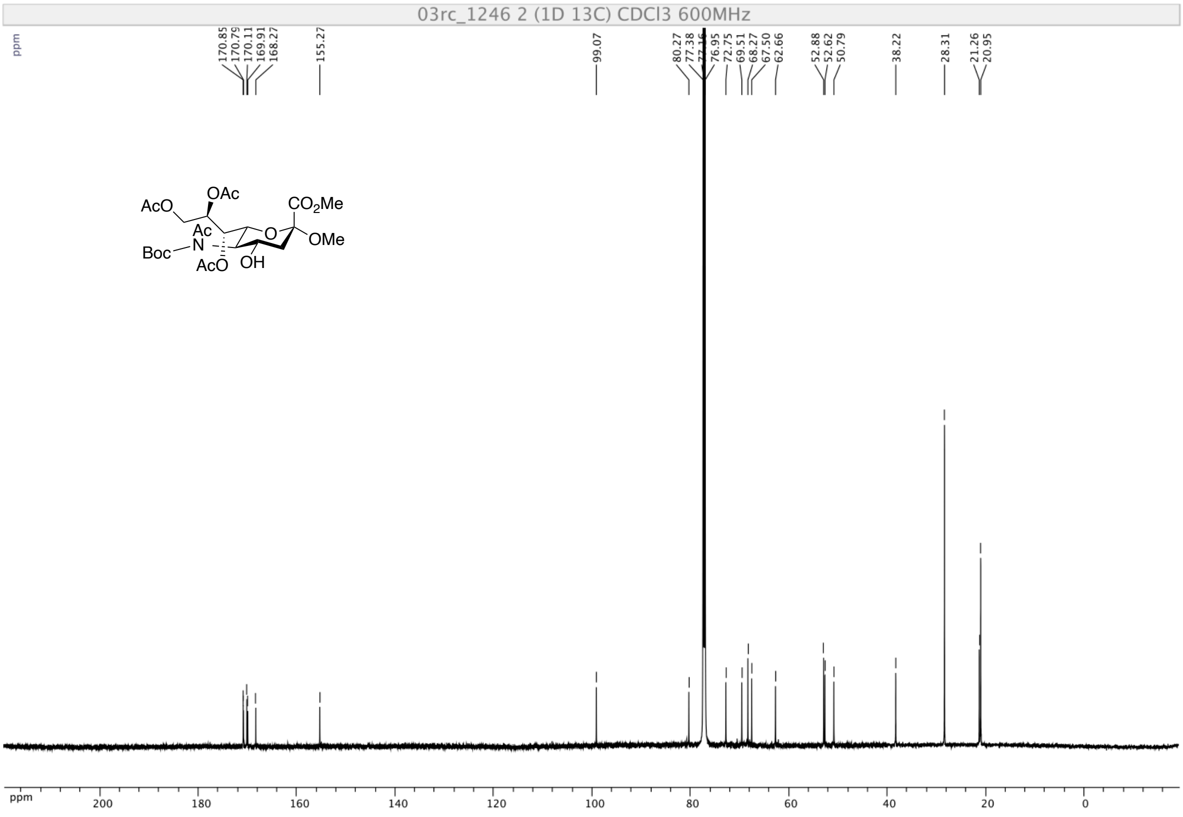
03rc_1246 2 (1D 13C) CDCl3 600MHz
AcO
OAc
CO2Me
Ac
N
Boc
AcO
OH
O
OMe
ppm

Compound **16**

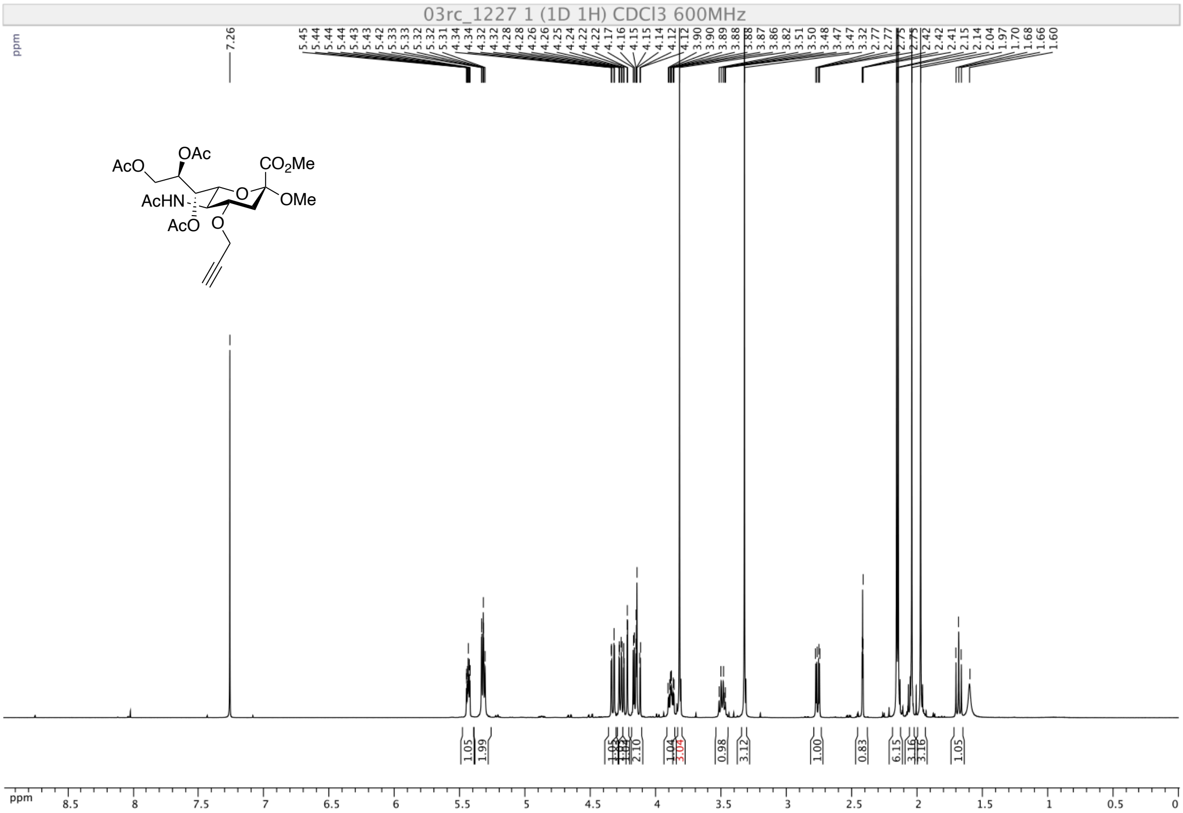
03rc_1227 1 (1D 1H) CDCl3 600MHz
ppm
7.26
5.45 5.44 5.44 5.43 5.43 5.42 5.33 5.33 5.32 5.32 5.31 4.34 4.32 4.28 4.28 4.26 4.26 4.25 4.24 4.22 4.22 4.17 4.16 4.15 4.15 4.14 4.12 4.12 3.90 3.90 3.89 3.88 3.88 3.87 3.86 3.82 3.51 3.50 3.48 3.47 3.47 3.32 2.77 2.77 2.75 2.75 2.42 2.41 2.15 2.14 2.04 1.97 1.70 1.68 1.66 1.60
AcO
OAc
CO2Me
AcHN
O
OMe
AcO
O
ppm 8.5 8 7.5 7 6.5 6 5.5 5 4.5 4 3.5 3 2.5 2 1.5 1 0.5 0

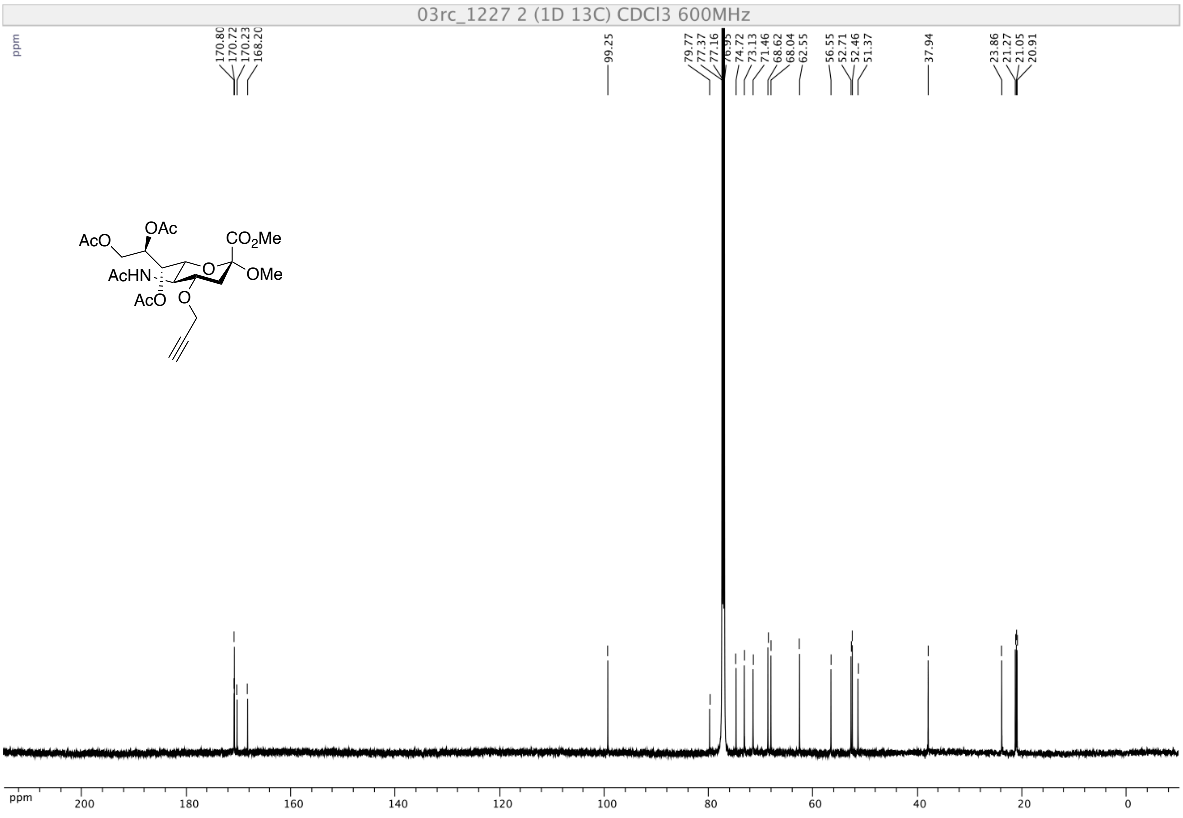
03rc_1227 2 (1D 13C) CDCl3 600MHz
ppm
170.80 170.72 170.23 168.20
99.25
79.77 77.37 77.16 76.95 74.72 73.13 71.46 68.62 68.04 62.55
56.55 52.71 52.46 51.37
37.94
23.86 21.27 21.05 20.91
AcO
OAc
CO2Me
AcHN
O
OMe
AcO
O
ppm 200 180 160 140 120 100 80 60 40 20 0

Compound **17**

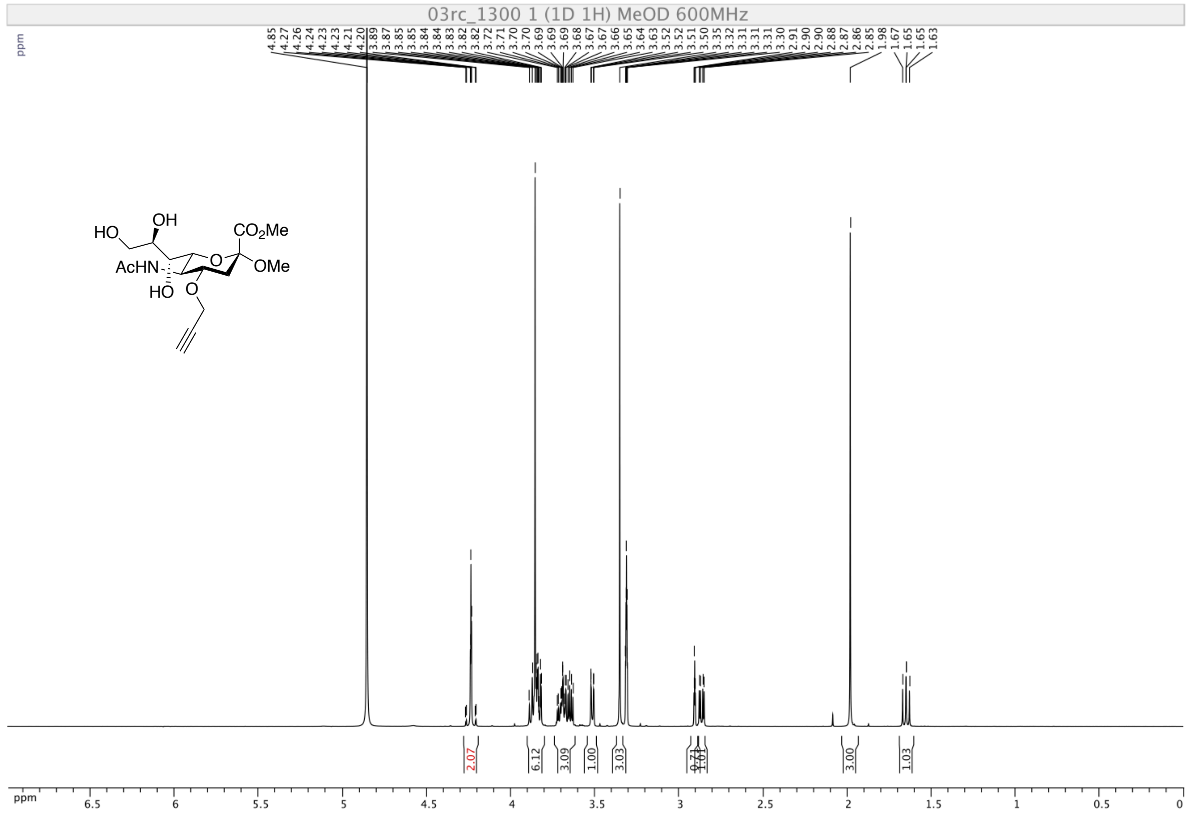

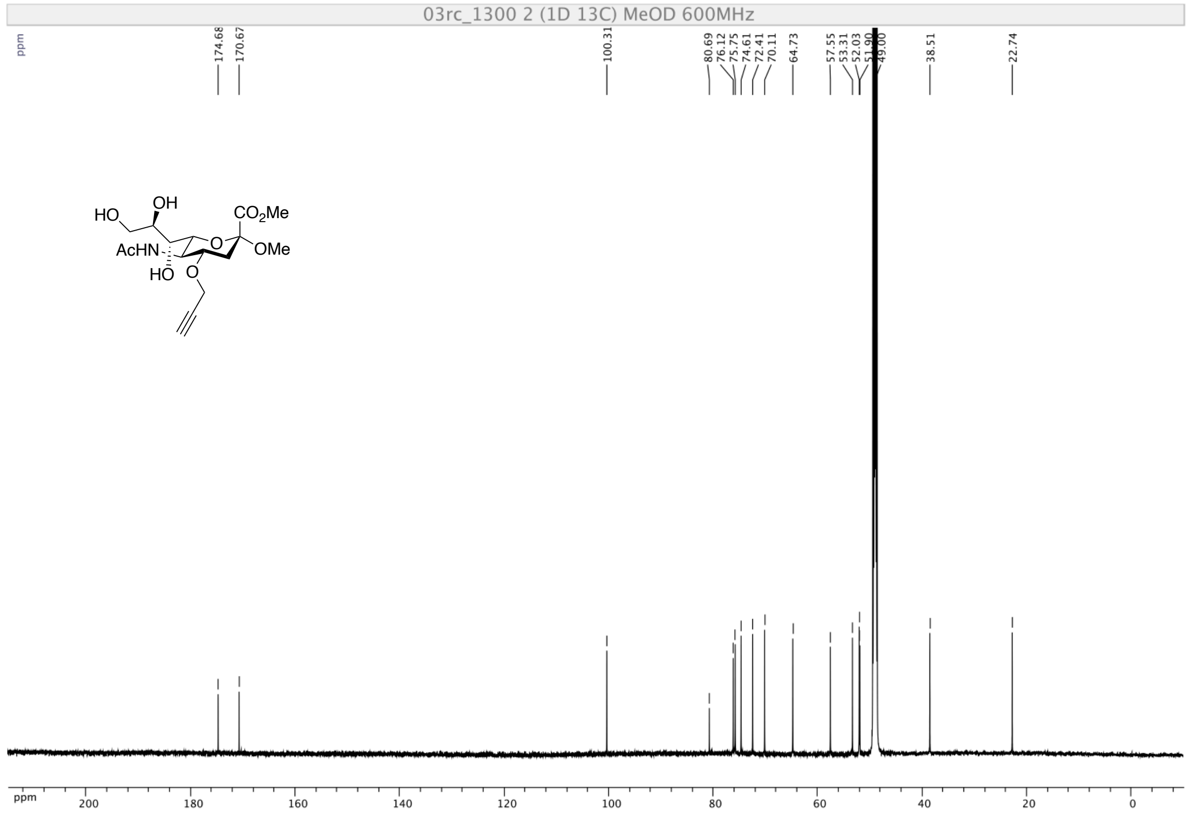

Compound **20**

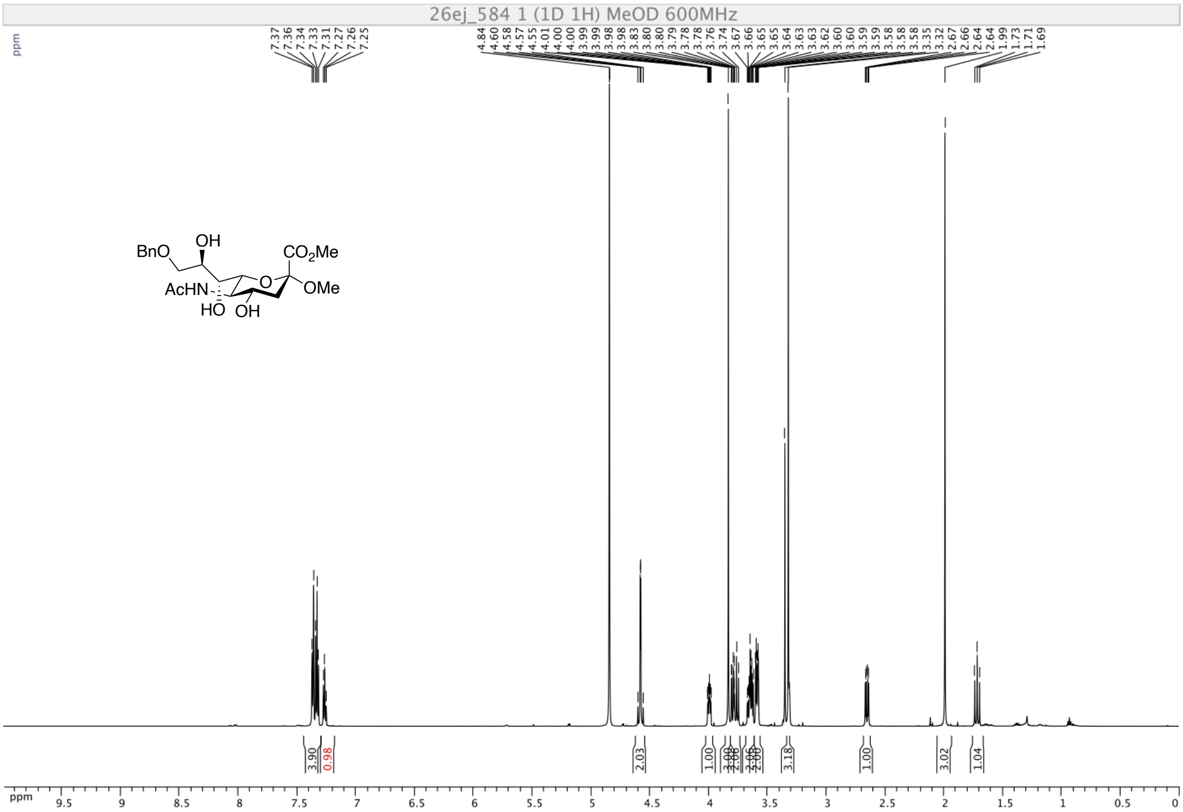
26ej_584 1 (1D 1H) MeOD 600MHz
BnO
OH
CO2Me
AcHN
O
OMe
HO
OH

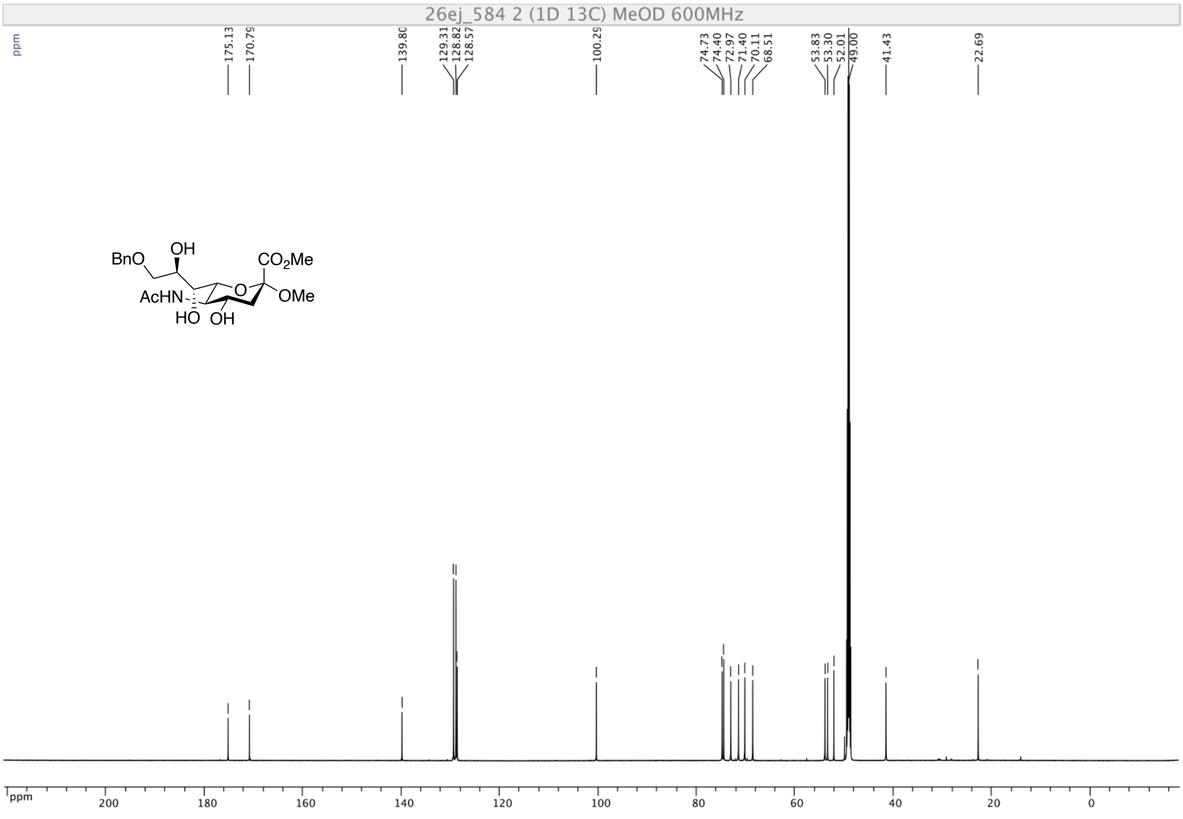
26ej_584 2 (1D 13C) MeOD 600MHz
175.13
170.79
139.80
129.31
128.82
128.57
100.29
74.73
74.40
72.97
71.40
70.11
68.51
53.83
53.30
52.01
49.00
41.43
22.69
BnO
OH
CO2Me
AcHN
O
OMe
HO
OH

Compound **18**

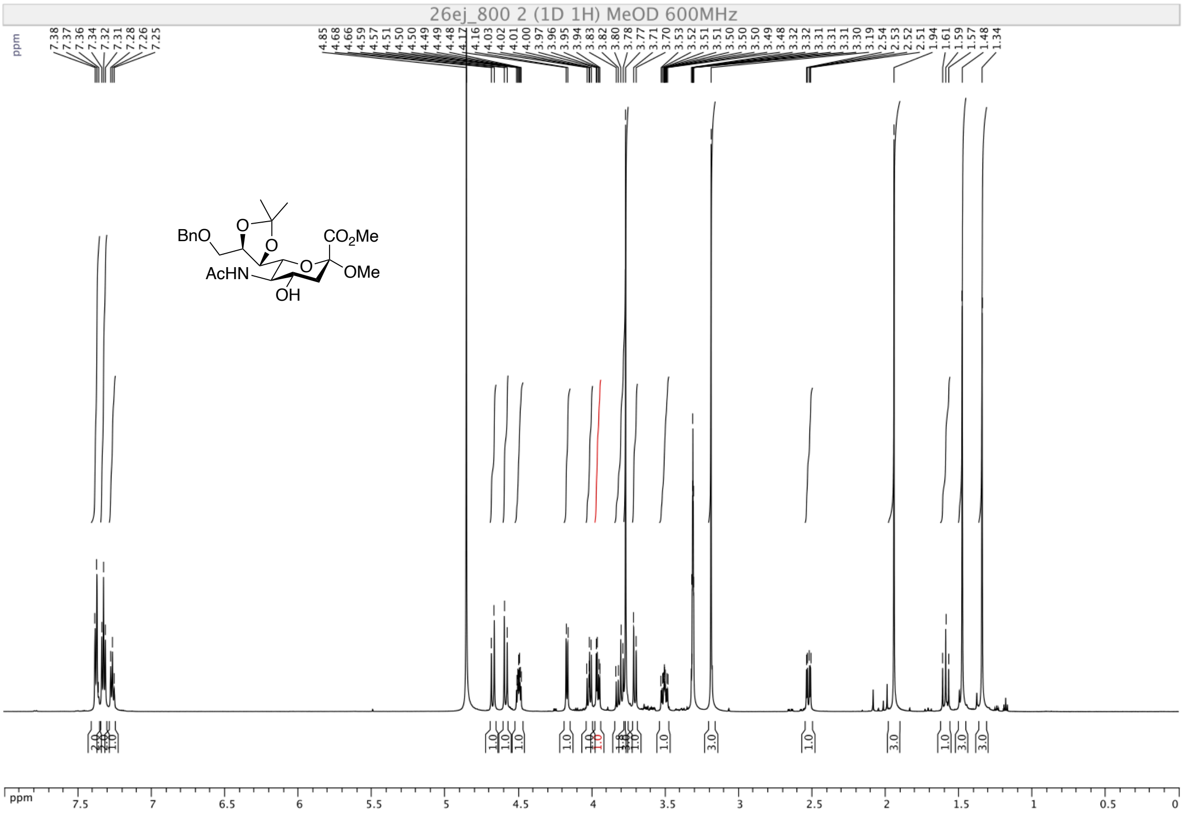
26ej_800 2 (1D 1H) MeOD 600MHz
BnO
AcHN
OH
CO2Me
OMe
ppm

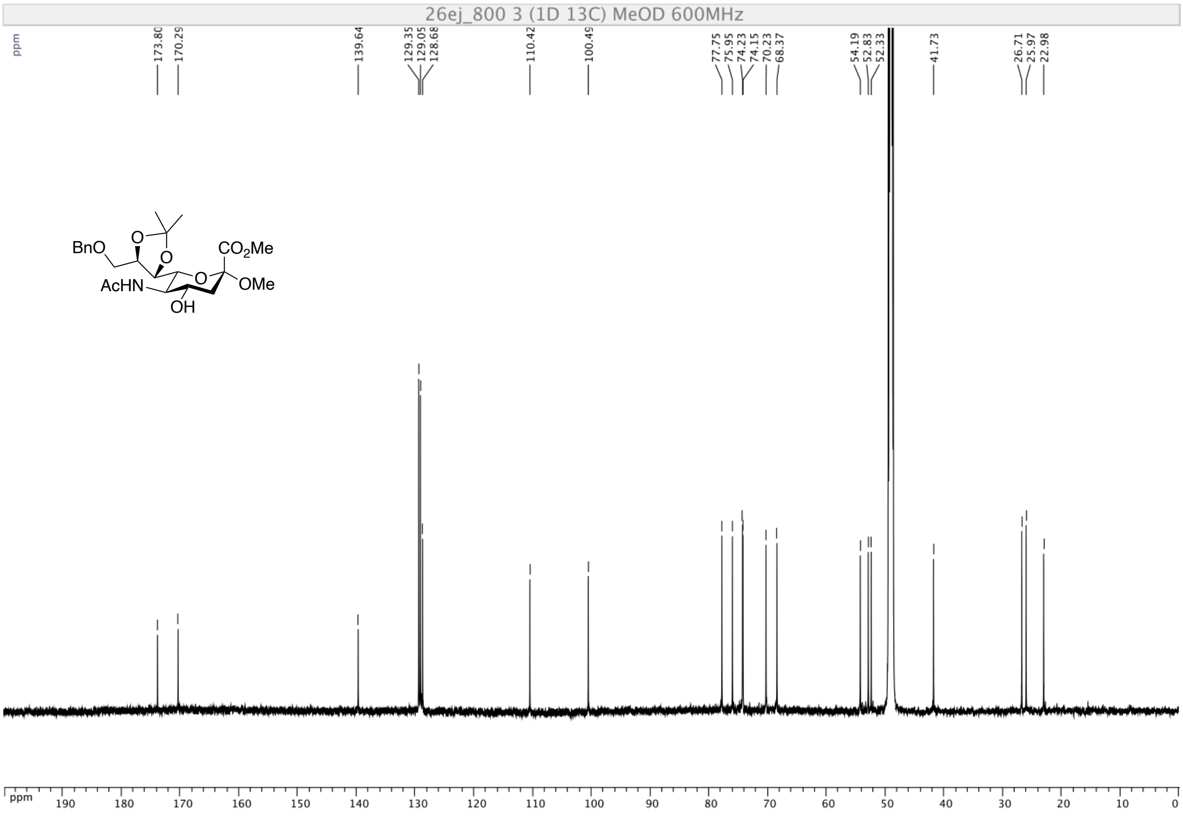
26ej_800 3 (1D 13C) MeOD 600MHz
173.80
170.29
139.64
129.35
129.05
128.68
110.42
100.49
77.75
75.95
74.23
74.15
70.23
68.37
54.19
52.83
52.33
41.73
26.71
25.97
22.98
BnO
AcHN
OH
CO2Me
OMe
ppm

Compound **22**

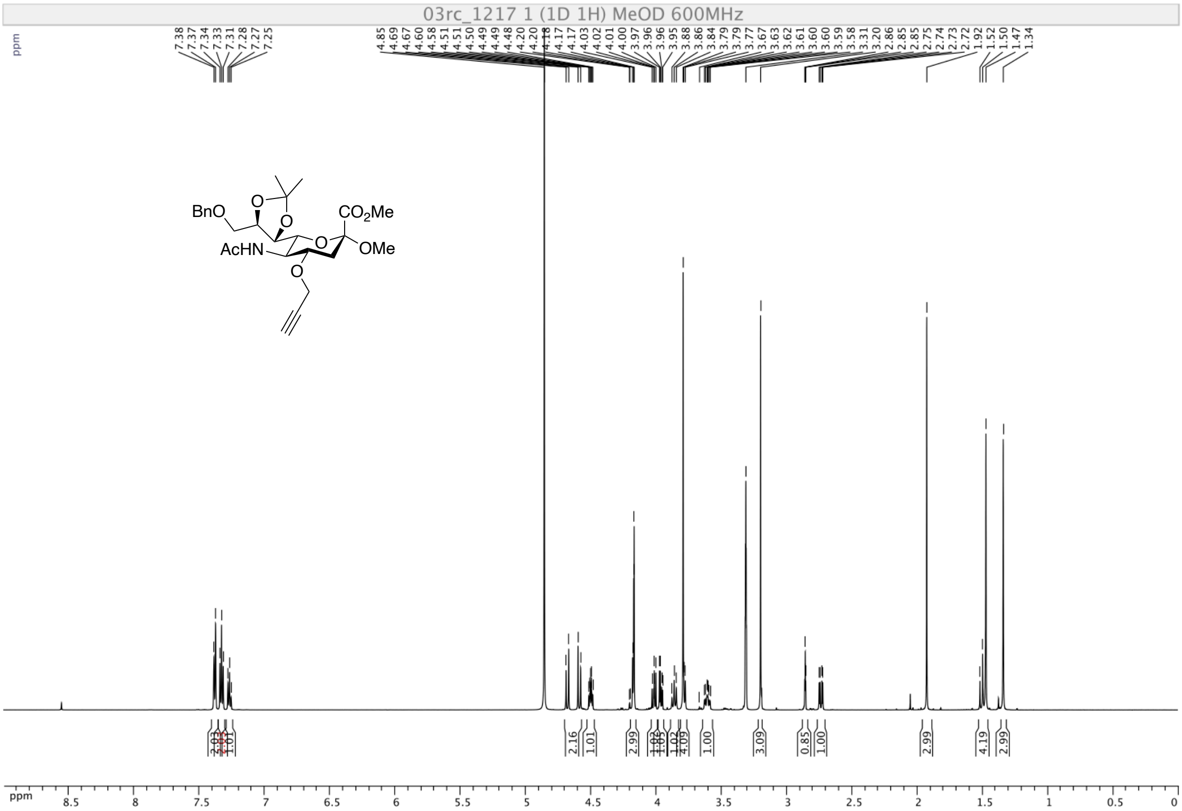
03rc_1217 1 (1D 1H) MeOD 600MHz
7.38 7.37 7.34 7.33 7.31 7.28 7.27 7.25
4.85 4.69 4.67 4.60 4.58 4.51 4.51 4.50 4.49 4.49 4.48 4.20 4.20 4.18 4.17 4.17 4.03 4.02 4.01 4.01 4.00 3.97 3.96 3.96 3.95 3.88 3.86 3.84 3.79 3.79 3.77 3.67 3.63 3.62 3.61 3.61 3.60 3.60 3.59 3.58 3.31 3.20 2.86 2.85 2.85 2.75 2.74 2.73 2.72 1.92 1.52 1.50 1.47 1.34
BnO
O
O
CO2Me
AcHN
O
OMe
O
ppm

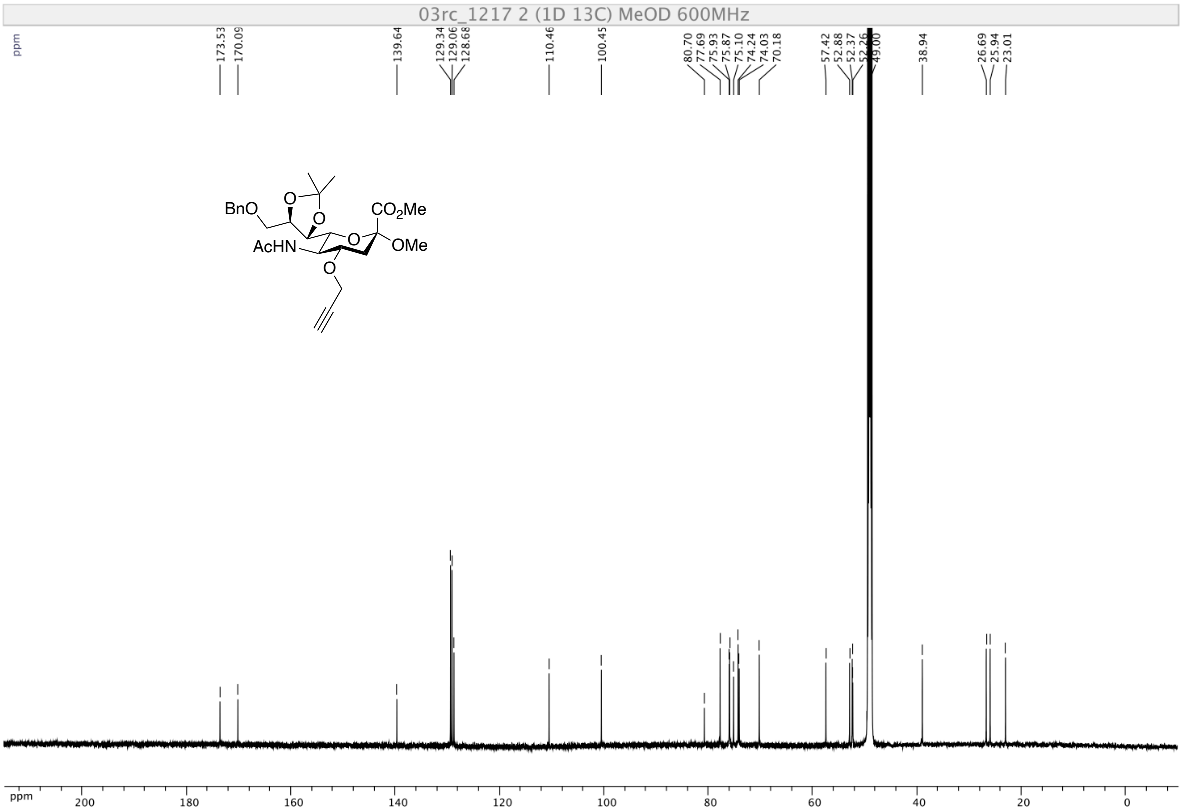
03rc_1217 2 (1D 13C) MeOD 600MHz
173.53 170.09 139.64 129.34 129.06 128.68 110.46 100.45 80.70 77.69 75.93 75.87 75.10 74.24 74.03 70.18 57.42 52.88 52.37 49.00 38.94 26.69 25.94 23.01
BnO
O
O
CO2Me
AcHN
O
OMe
O
ppm

Compound **23**

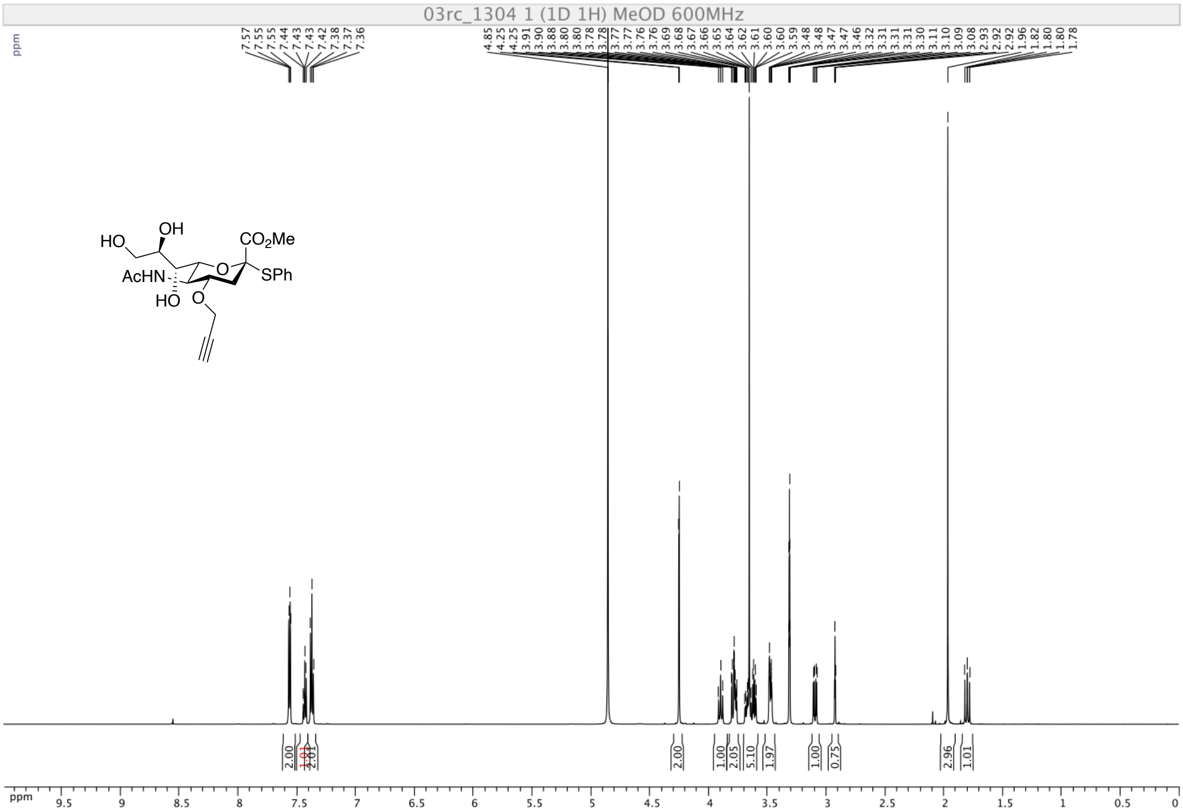
03rc_1304 1 (1D 1H) MeOD 600MHz
HO
OH
CO_2Me
O
AcHN
SPh
HO
O
ppm

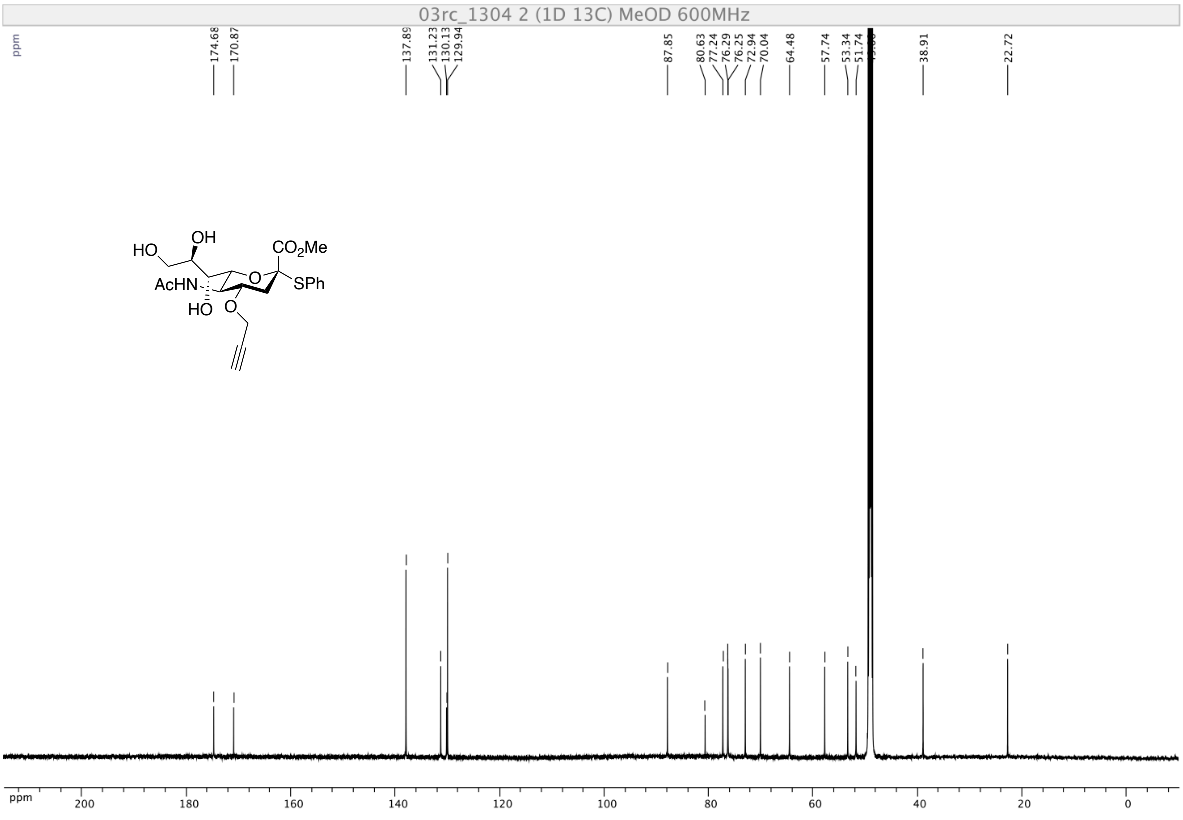
03rc_1304 2 (1D 13C) MeOD 600MHz
174.68
170.87
137.89
131.23
130.13
129.94
87.85
80.63
77.24
76.29
76.25
72.94
70.04
64.48
57.74
53.34
51.74
38.91
22.72
HO
OH
CO_2Me
O
AcHN
SPh
HO
O
ppm

Compound **24**

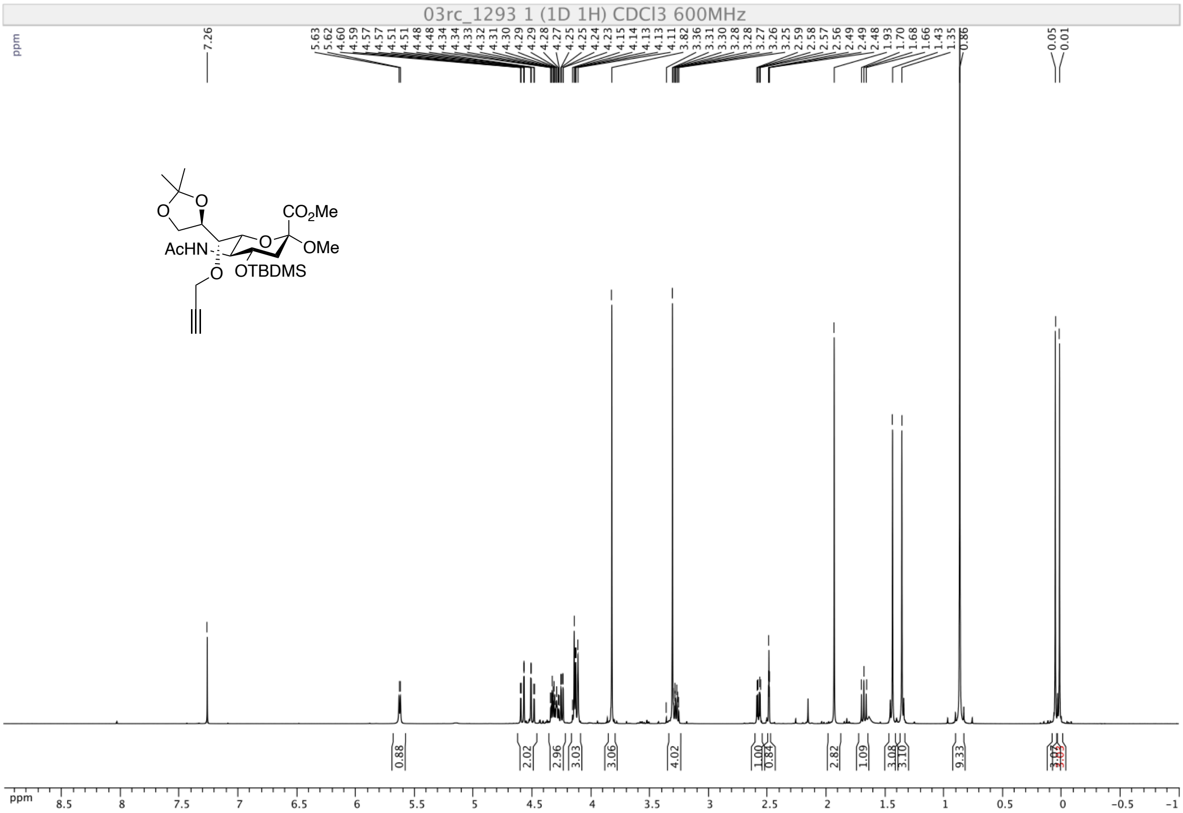
03rc_1293 1 (1D 1H) CDCl3 600MHz
CO2Me
OMe
AcHN
OTBDMS
O
ppm

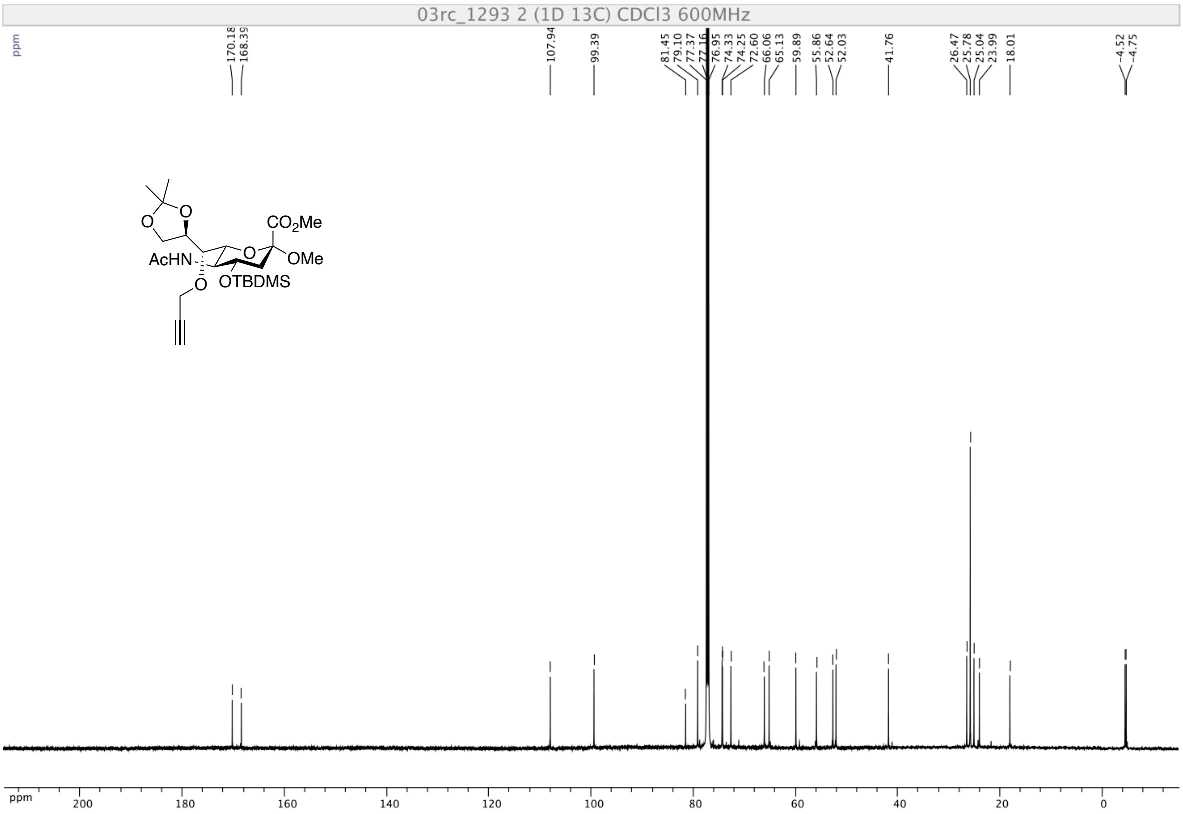
03rc_1293 2 (1D 13C) CDCl3 600MHz
170.18
168.39
107.94
99.39
81.45
79.10
77.37
77.16
76.95
74.33
74.25
72.60
66.06
65.13
59.89
55.86
52.64
52.03
41.76
26.47
25.78
25.04
23.99
18.01
-4.52
-4.75
CO2Me
OMe
AcHN
OTBDMS
O
ppm

Compound **25**

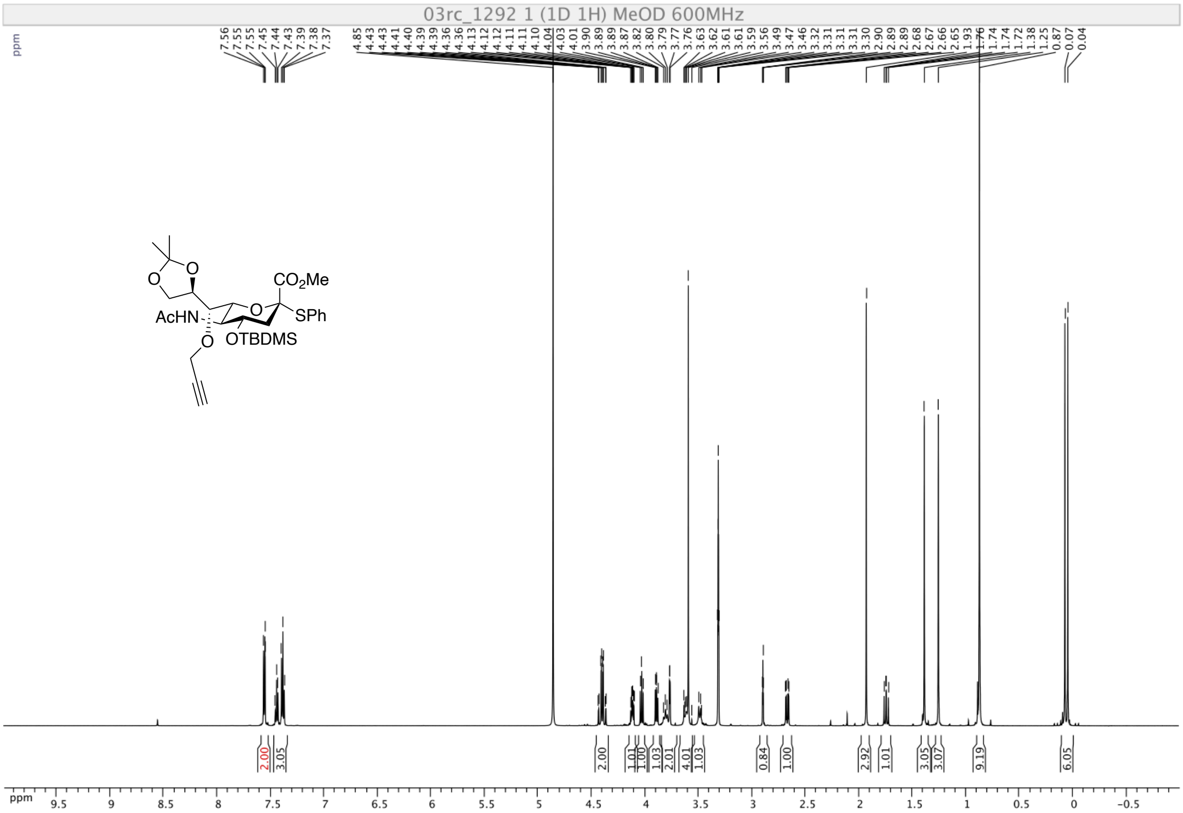
03rc_1292 1 (1D 1H) MeOD 600MHz
ppm
7.56 7.55 7.55 7.45 7.44 7.43 7.39 7.38 7.37 4.85 4.43 4.43 4.41 4.40 4.39 4.39 4.36 4.36 4.13 4.12 4.12 4.11 4.11 4.10 4.04 4.03 4.01 3.90 3.89 3.89 3.87 3.82 3.80 3.79 3.77 3.76 3.63 3.62 3.61 3.61 3.59 3.56 3.49 3.47 3.46 3.32 3.31 3.31 3.31 3.30 2.90 2.89 2.89 2.68 2.67 2.66 2.65 1.93 1.76 1.74 1.72 1.38 1.25 0.87 0.07 0.04
CO2Me
SPh
AcHN
O
OTBDMS
2.00 3.05 2.00 1.01 1.00 1.03 2.01 4.01 1.03 0.84 1.00 2.92 1.01 3.05 3.07 9.19 6.05
ppm 9.5 9 8.5 8 7.5 7 6.5 6 5.5 5 4.5 4 3.5 3 2.5 2 1.5 1 0.5 0 -0.5

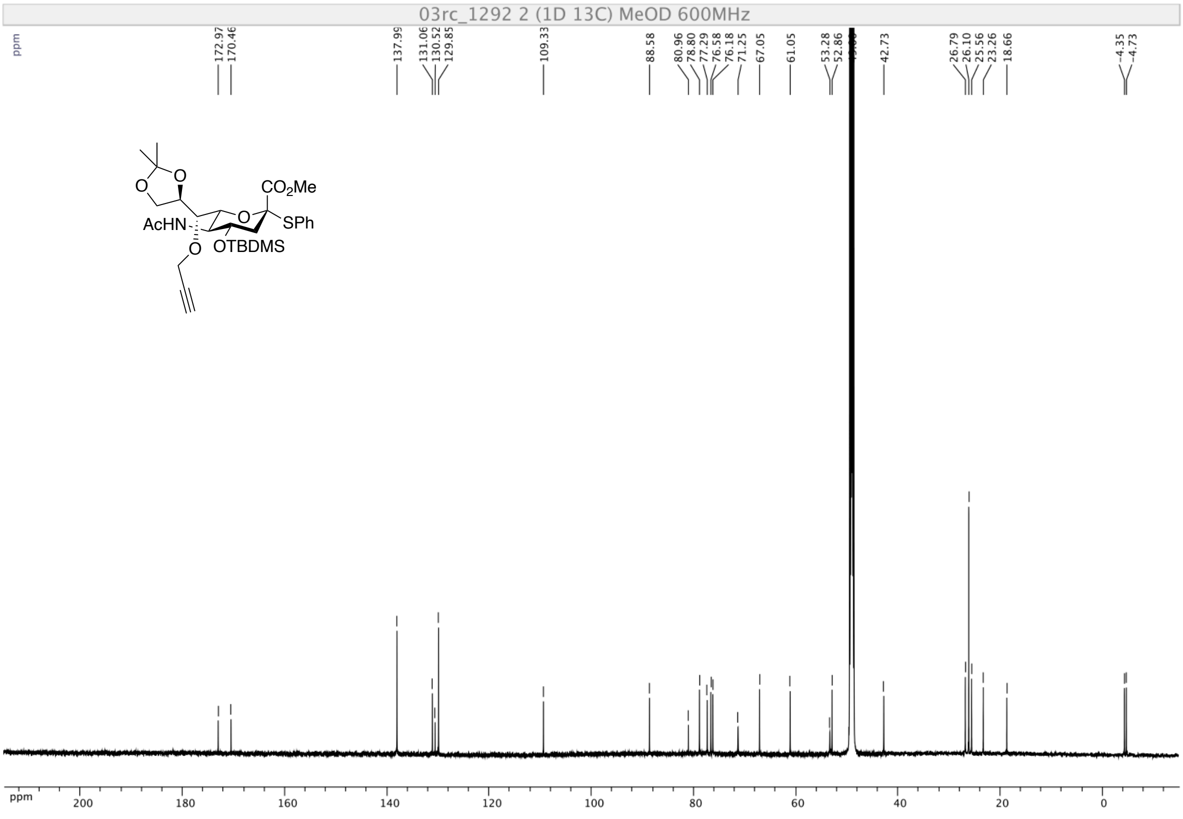
03rc_1292 2 (1D 13C) MeOD 600MHz
ppm
172.97 170.46 137.99 131.06 130.52 129.85 109.33 88.58 80.96 78.80 77.29 76.58 76.18 71.25 67.05 61.05 53.28 52.86 42.73 26.79 26.10 25.56 23.26 18.66 -4.35 -4.73
CO2Me
SPh
AcHN
O
OTBDMS
ppm 200 180 160 140 120 100 80 60 40 20 0

Compound **26**

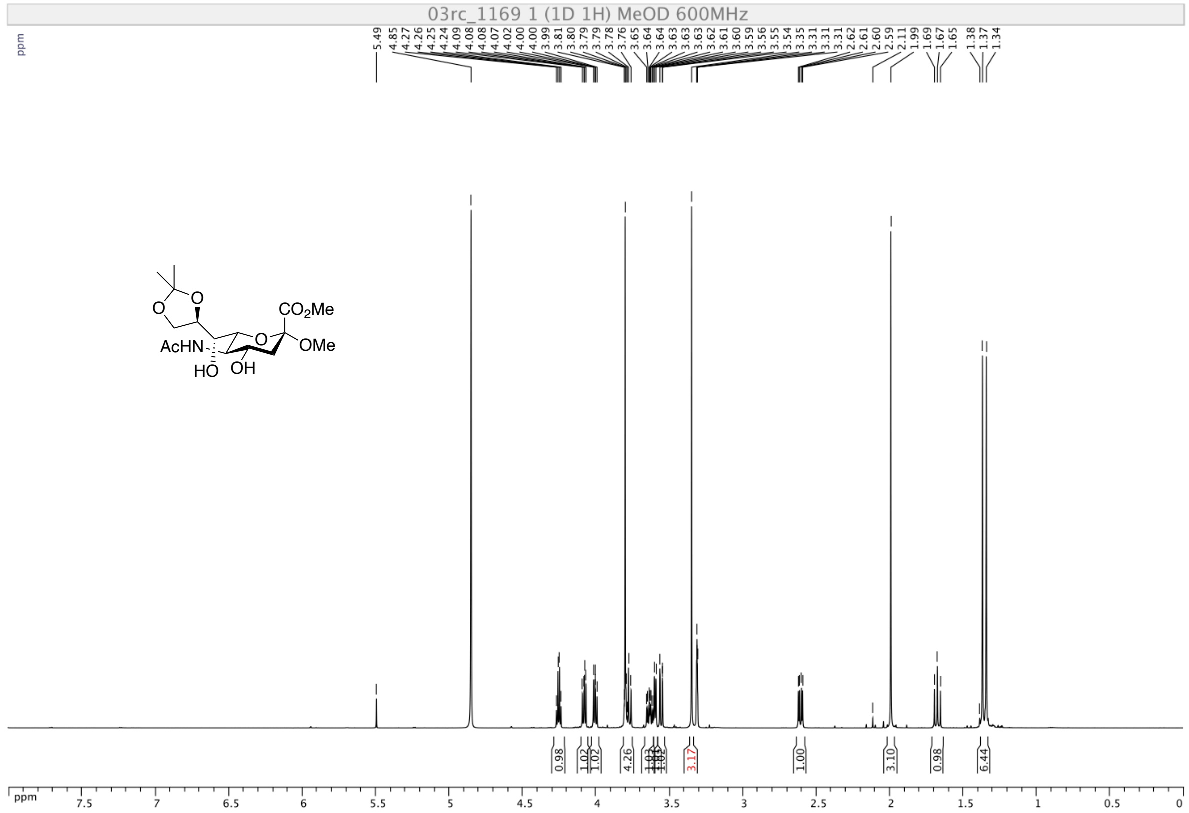
03rc_1169 1 (1D 1H) MeOD 600MHz
ppm
5.49 4.85 4.27 4.26 4.25 4.24 4.09 4.08 4.08 4.07 4.02 4.00 4.00 3.99 3.81 3.80 3.79 3.79 3.78 3.76 3.65 3.64 3.64 3.63 3.63 3.63 3.62 3.61 3.60 3.59 3.56 3.55 3.54 3.35 3.31 3.31 3.31 2.62 2.61 2.60 2.59 2.11 1.99 1.69 1.67 1.65 1.38 1.37 1.34
O
O
CO2Me
AcHN
O
OMe
HO
OH
0.98
1.02
1.02
4.26
3.17
1.00
3.10
0.98
6.44
ppm
7.5
7
6.5
6
5.5
5
4.5
4
3.5
3
2.5
2
1.5
1
0.5
0

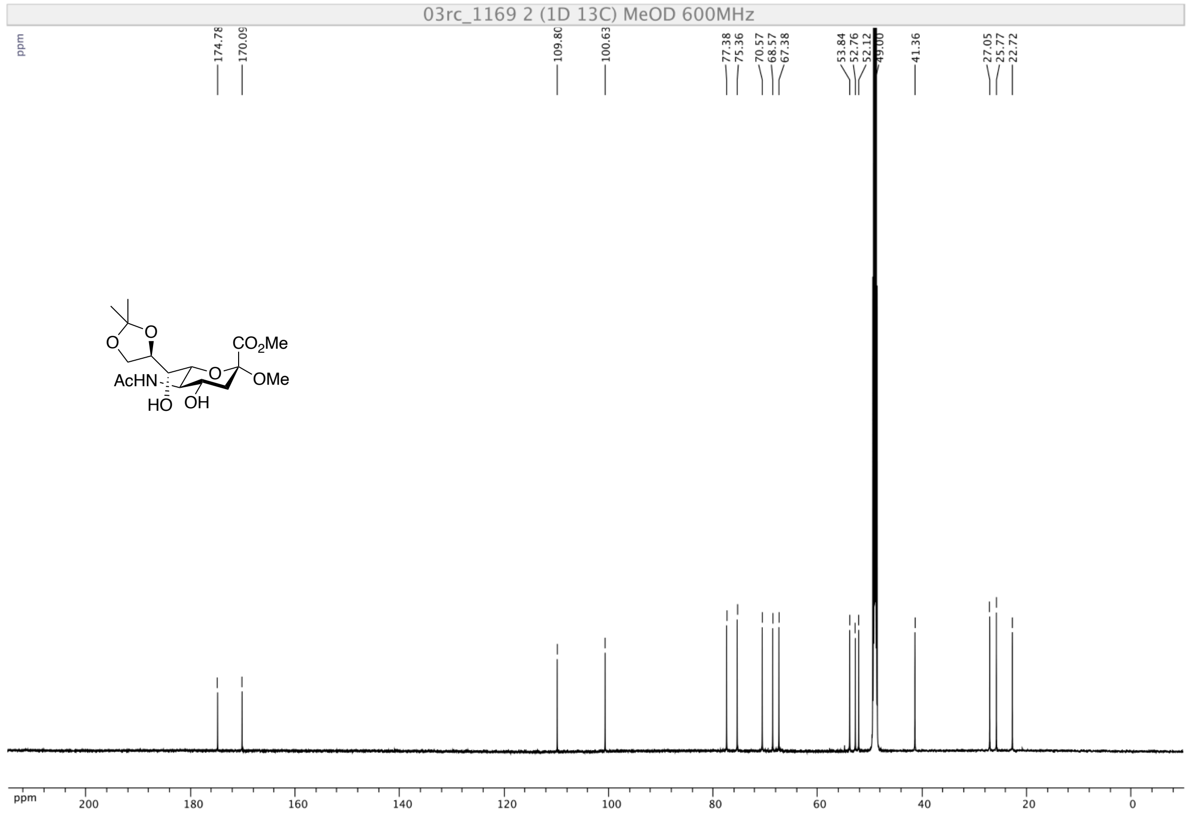
03rc_1169 2 (1D 13C) MeOD 600MHz
ppm
174.78 170.09 109.80 100.63 77.38 75.36 70.57 68.57 67.38 53.84 52.76 52.12 49.00 41.36 27.05 25.77 22.72
O
O
CO2Me
AcHN
O
OMe
HO
OH
ppm
200
180
160
140
120
100
80
60
40
20
0

Compound **27**

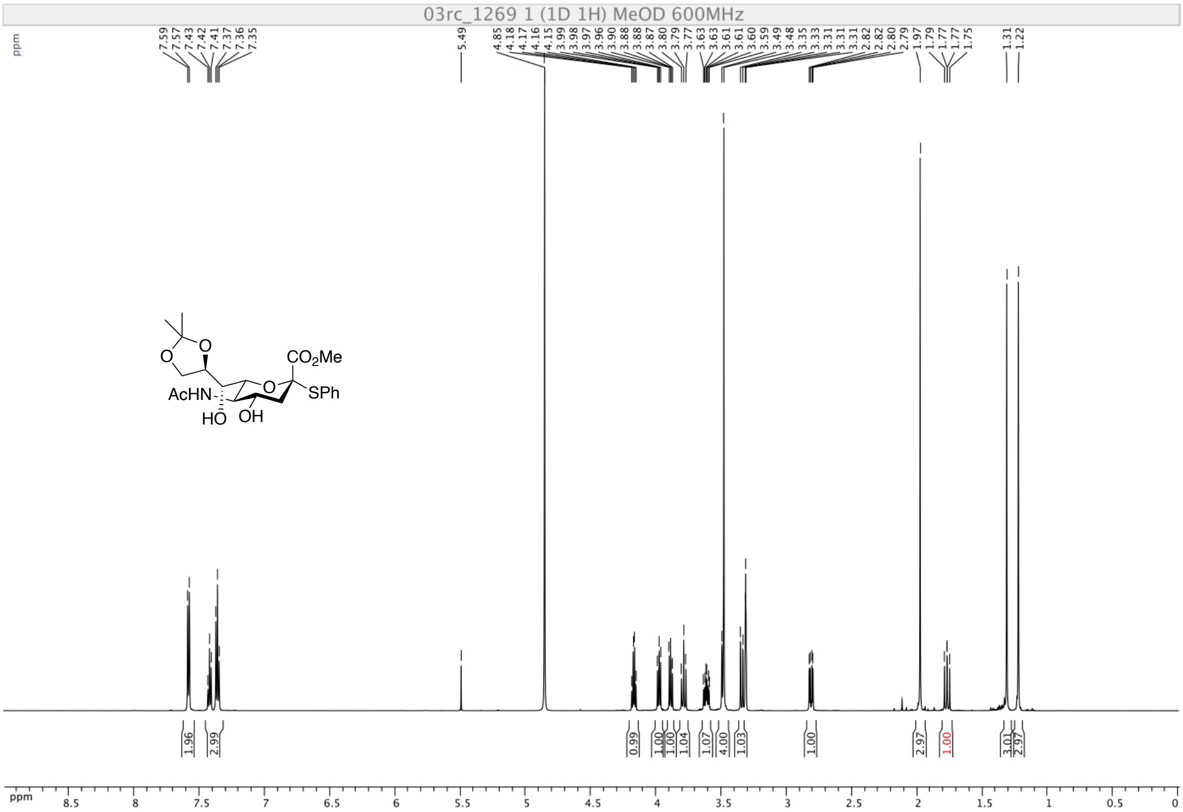
03rc_1269 1 (1D 1H) MeOD 600MHz
CO2Me
AcHN
SPh
HO
OH
ppm

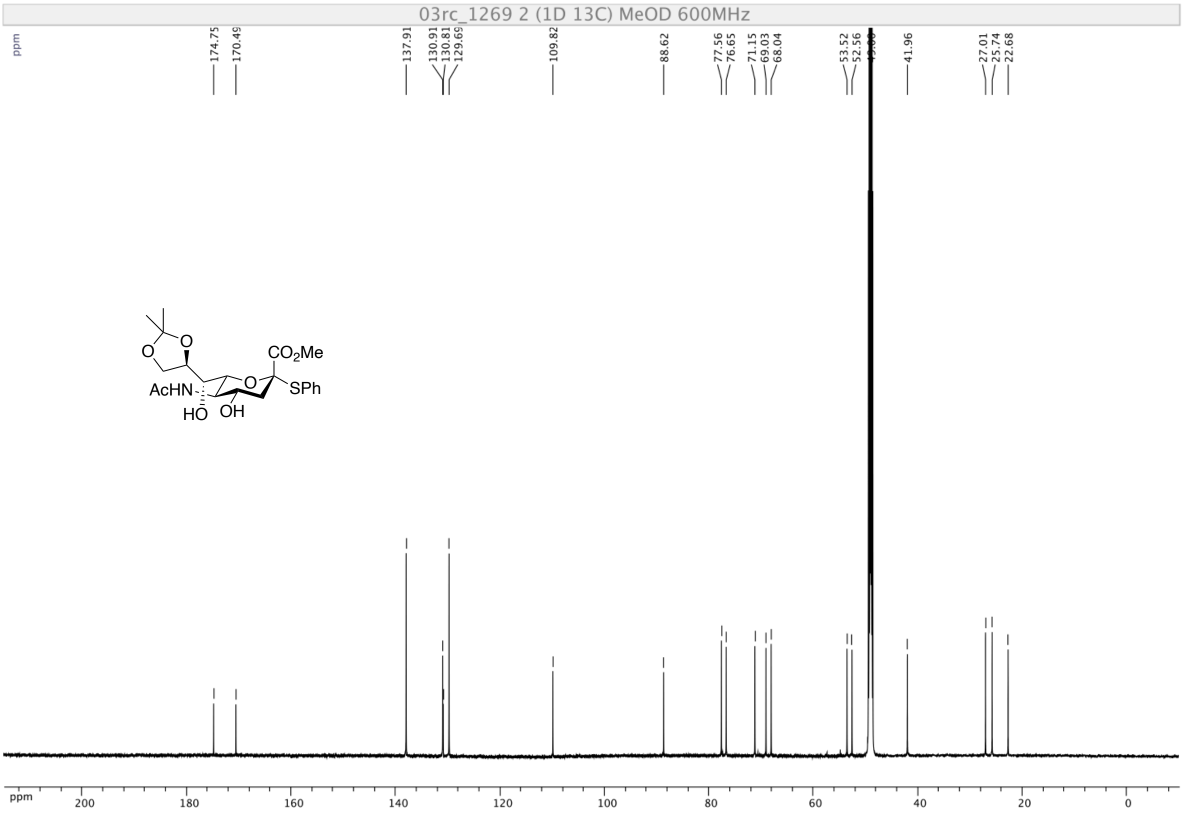
03rc_1269 2 (1D 13C) MeOD 600MHz
174.75
170.49
137.91
130.91
130.81
129.69
109.82
88.62
77.56
76.65
71.15
69.03
68.04
53.52
52.56
41.96
27.01
25.74
22.68
CO2Me
AcHN
SPh
HO
OH
ppm

Compound **28**

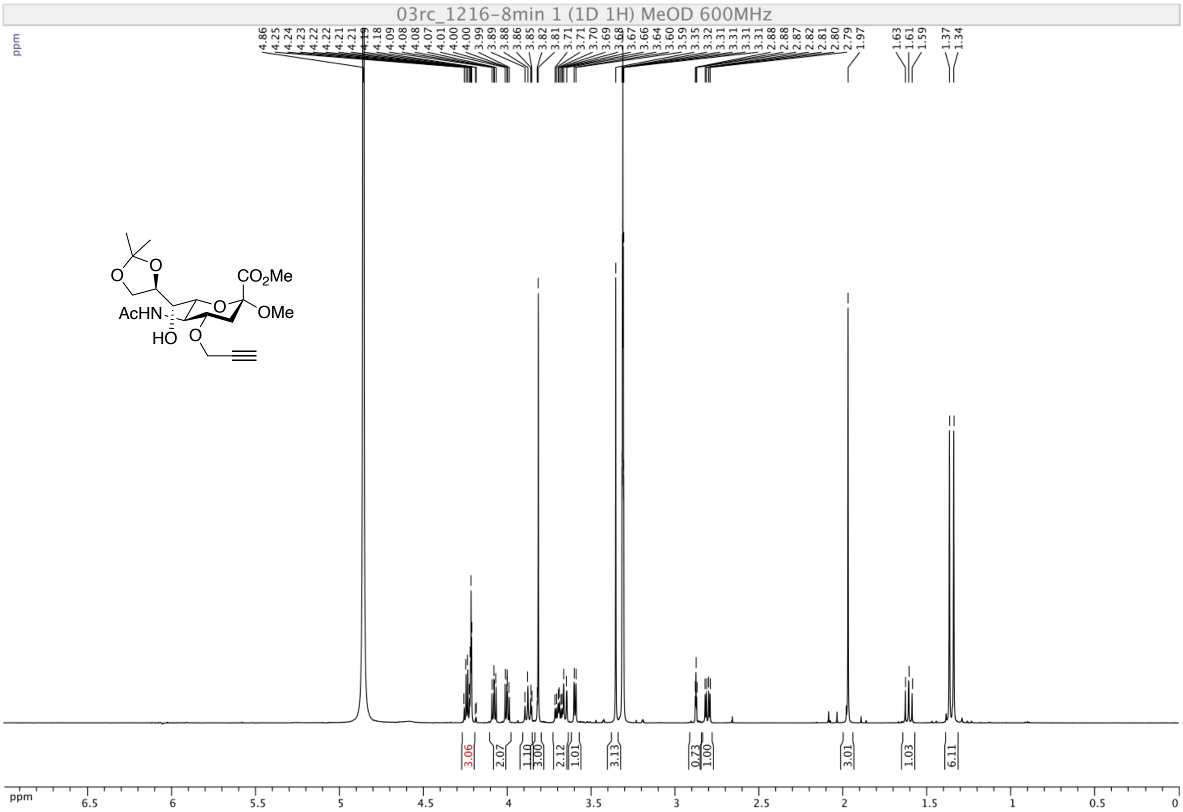
03rc_1216-8min 1 (1D 1H) MeOD 600MHz
ppm
4.86 4.25 4.24 4.23 4.22 4.22 4.21 4.21 4.19 4.18 4.09 4.08 4.08 4.07 4.01 4.00 4.00 3.99 3.89 3.88 3.86 3.85 3.82 3.81 3.71 3.71 3.70 3.69 3.68 3.67 3.66 3.64 3.60 3.59 3.35 3.32 3.31 3.31 3.31 3.31 3.31 2.88 2.88 2.87 2.82 2.81 2.80 2.79 1.97 1.63 1.61 1.59 1.37 1.34
O
O
CO2Me
OMe
AcHN
HO
3.06 2.07 1.10 3.00 2.12 1.01 3.13 0.73 1.00 3.01 1.03 6.11
ppm 6.5 6 5.5 5 4.5 4 3.5 3 2.5 2 1.5 1 0.5 0

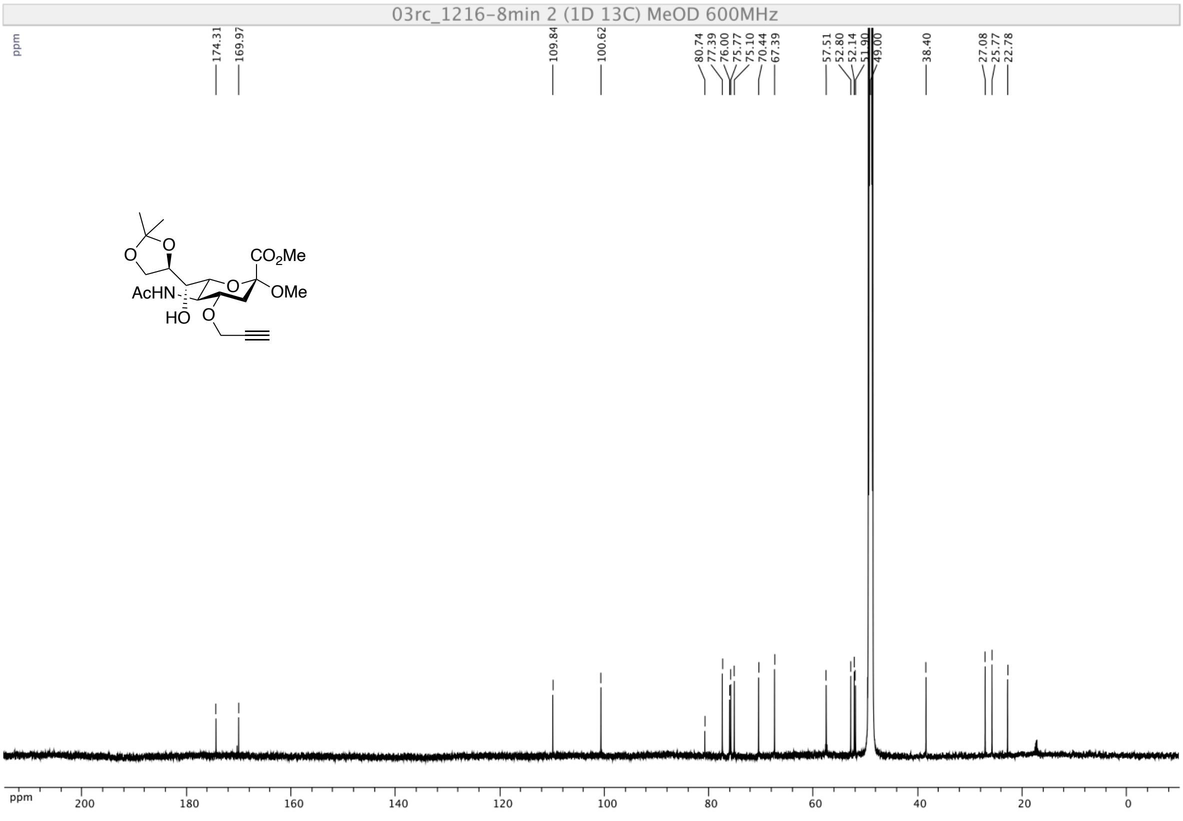
03rc_1216-8min 2 (1D 13C) MeOD 600MHz
ppm
174.31 169.97 109.84 100.62 80.74 77.39 76.00 75.77 75.10 70.44 67.39 57.51 52.80 52.14 51.90 49.00 38.40 27.08 25.77 22.78
O
O
CO2Me
OMe
AcHN
HO
ppm 200 180 160 140 120 100 80 60 40 20 0

Compound **29**

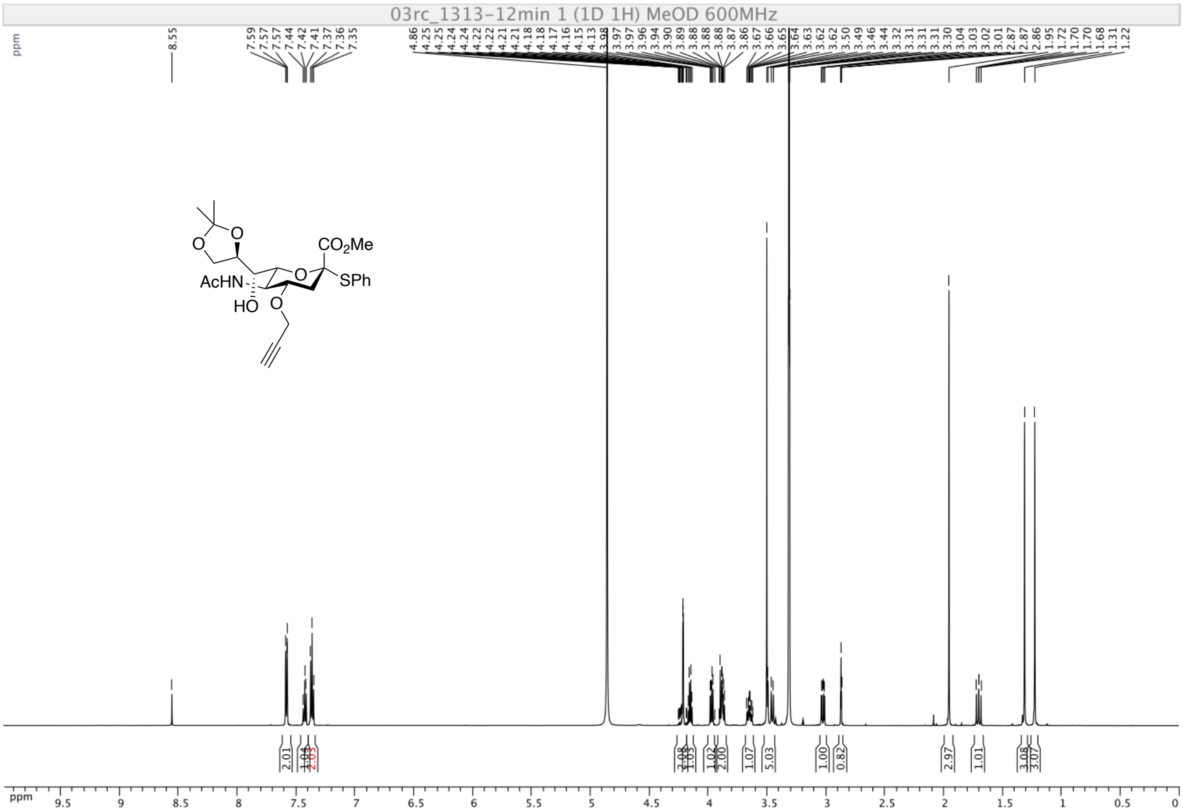
03rc_1313-12min 1 (1D 1H) MeOD 600MHz
CO2Me
SPh
AcHN
HO
O
ppm

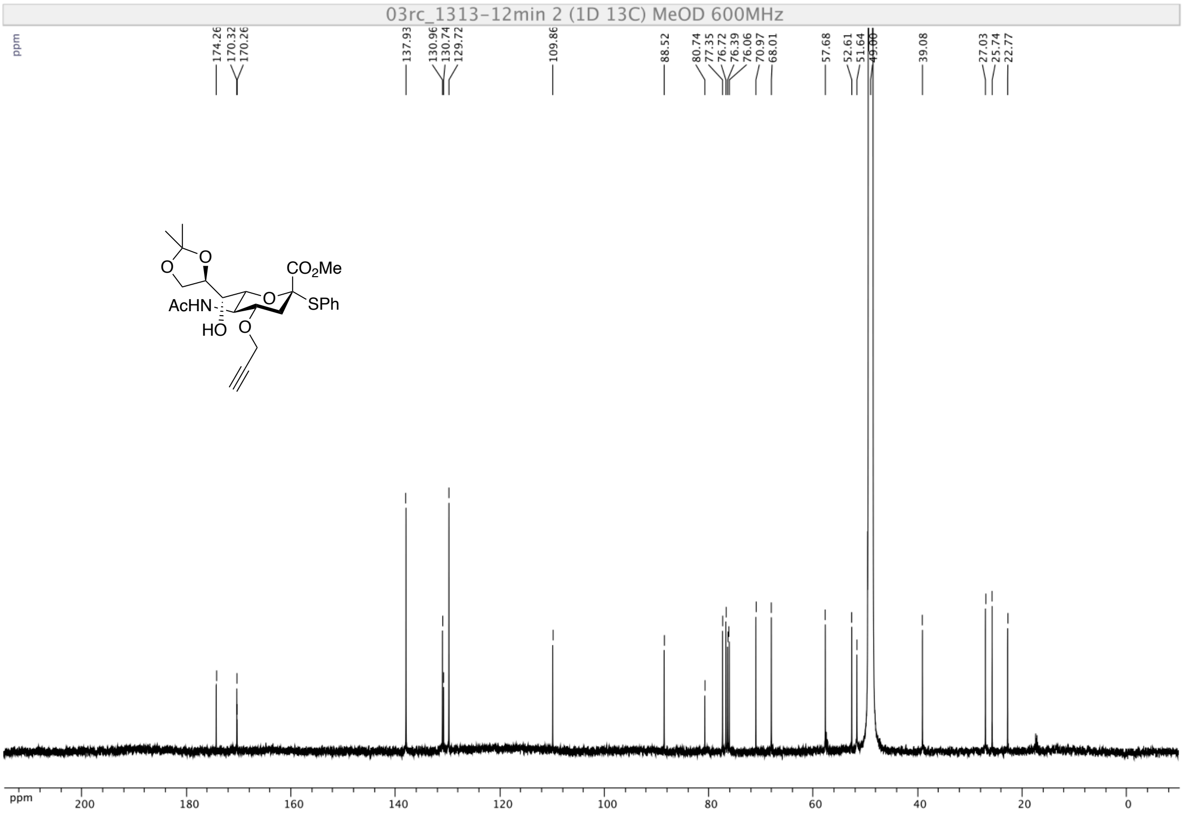
03rc_1313-12min 2 (1D 13C) MeOD 600MHz
174.26
170.32
170.26
137.93
130.96
130.74
129.72
109.86
88.52
80.74
77.35
76.72
76.39
76.06
70.97
68.01
57.68
52.61
51.64
39.08
27.03
25.74
22.77
CO2Me
SPh
AcHN
HO
O
ppm

Compound **30**

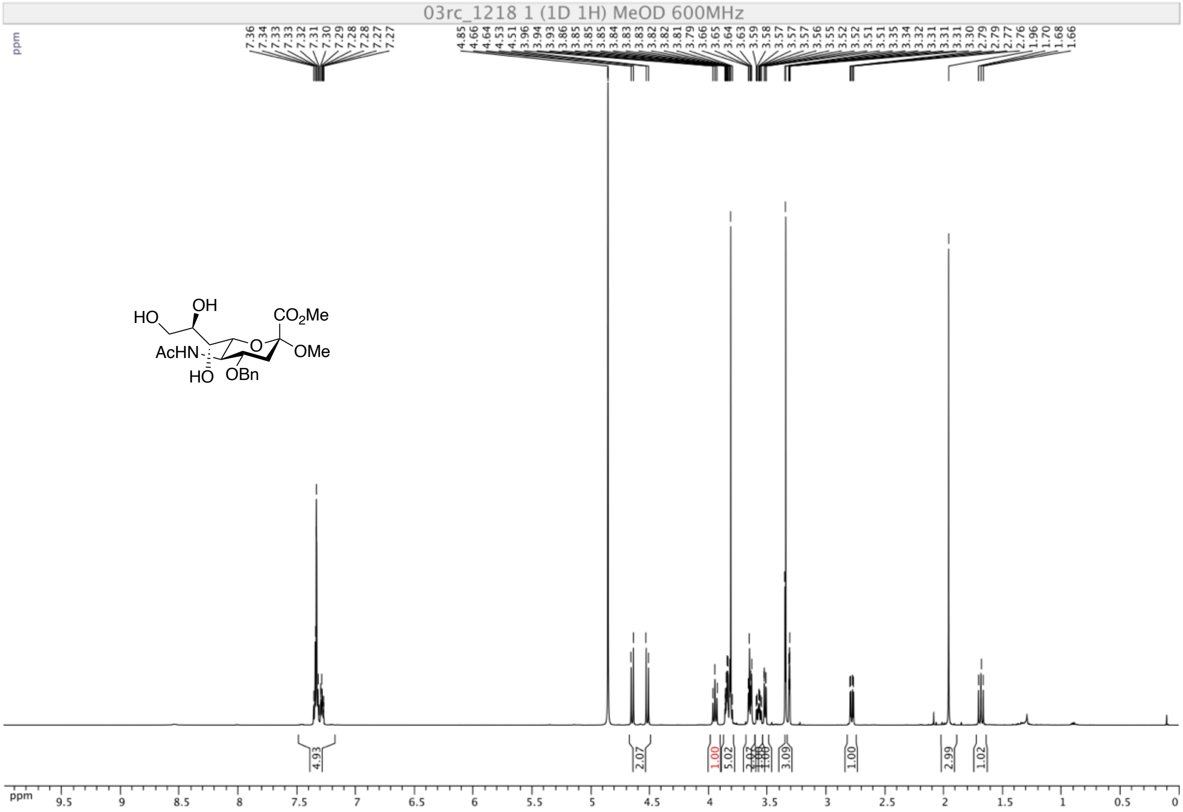
03rc_1218 1 (1D 1H) MeOD 600MHz
7.36 7.34 7.33 7.32 7.31 7.30 7.29 7.28 7.28 7.27 7.27
4.85 4.66 4.64 4.53 4.51 3.96 3.94 3.93 3.86 3.85 3.85 3.84 3.83 3.83 3.82 3.82 3.81 3.79 3.66 3.65 3.64 3.63 3.59 3.58 3.57 3.57 3.57 3.56 3.55 3.52 3.52 3.51 3.51 3.35 3.34 3.32 3.31 3.31 3.31 3.30 2.79 2.79 2.77 2.76 1.96 1.70 1.68 1.66
HO
OH
CO2Me
AcHN
O
OMe
HO
OBn
4.93
2.07
1.00
5.02
3.09
1.00
2.99
1.02
ppm

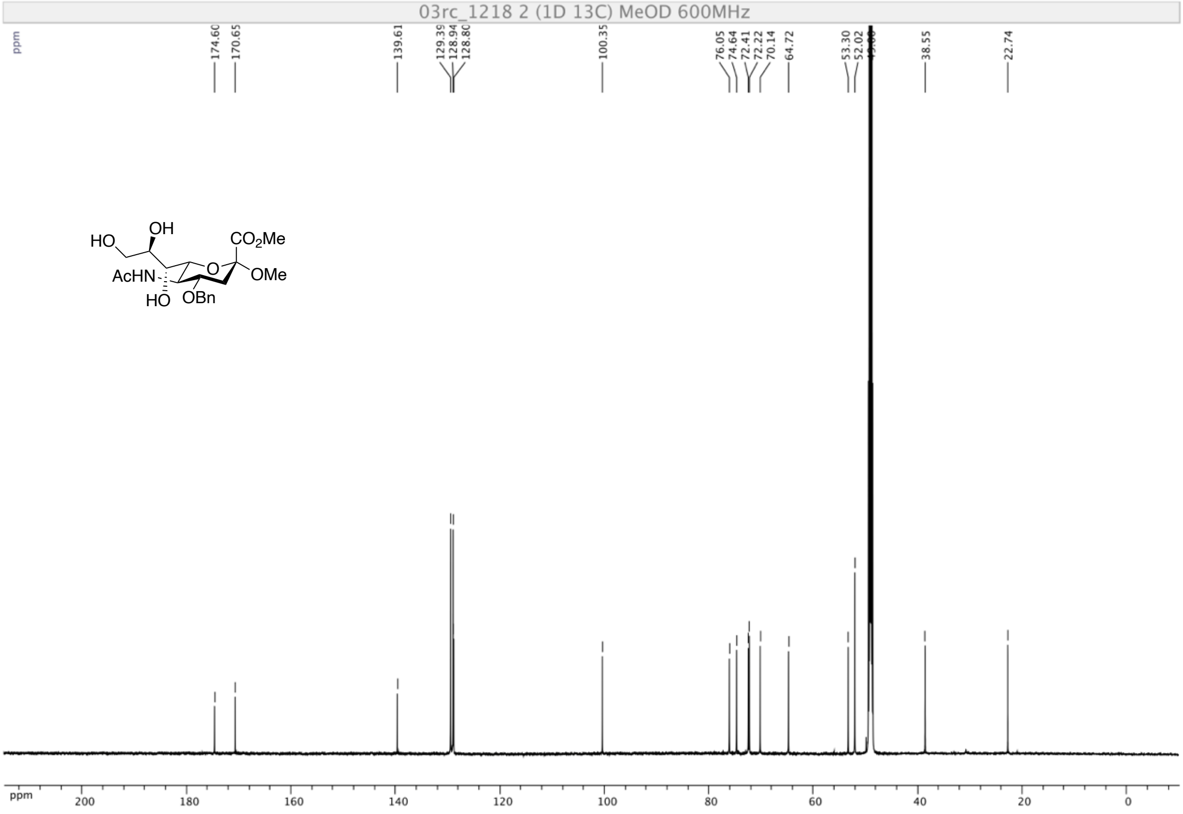
03rc_1218 2 (1D 13C) MeOD 600MHz
174.60 170.65 139.61 129.39 128.94 128.80 100.35 76.05 74.64 72.41 72.22 70.14 64.72 53.30 52.02 38.55 22.74
HO
OH
CO2Me
AcHN
O
OMe
HO
OBn
ppm

Compound **31**

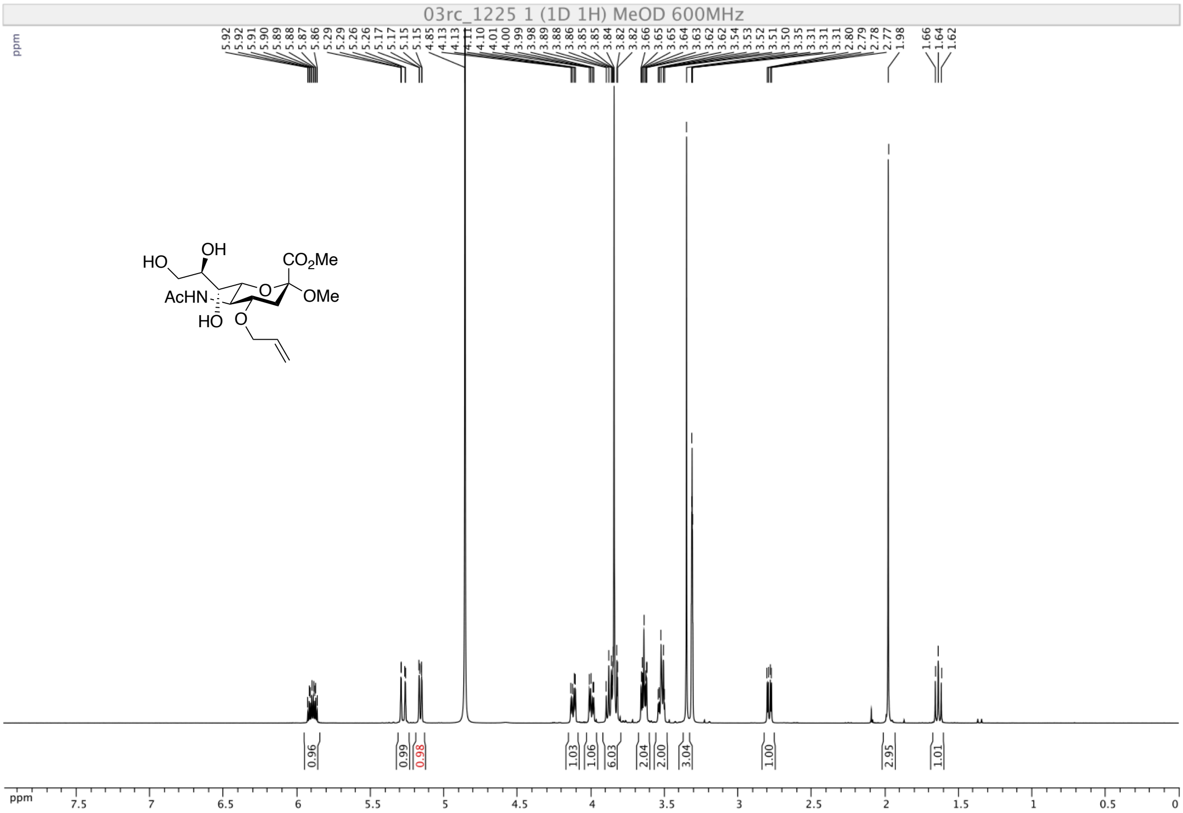
03rc_1225 1 (1D 1H) MeOD 600MHz
ppm
5.92 5.92 5.91 5.90 5.89 5.88 5.87 5.86 5.29 5.29 5.26 5.26 5.17 5.17 5.15 5.15 4.85 4.13 4.13 4.11 4.10 4.01 4.00 3.99 3.98 3.89 3.88 3.86 3.85 3.85 3.84 3.82 3.82 3.66 3.65 3.65 3.64 3.63 3.62 3.62 3.54 3.53 3.52 3.51 3.50 3.35 3.31 3.31 3.31 2.80 2.79 2.78 2.77 1.98 1.66 1.64 1.62
HO
OH
CO2Me
O
OMe
AcHN
HO
O
0.96 0.99 0.98 1.03 1.06 6.03 2.04 2.00 3.04 1.00 2.95 1.01
ppm 7.5 7 6.5 6 5.5 5 4.5 4 3.5 3 2.5 2 1.5 1 0.5 0

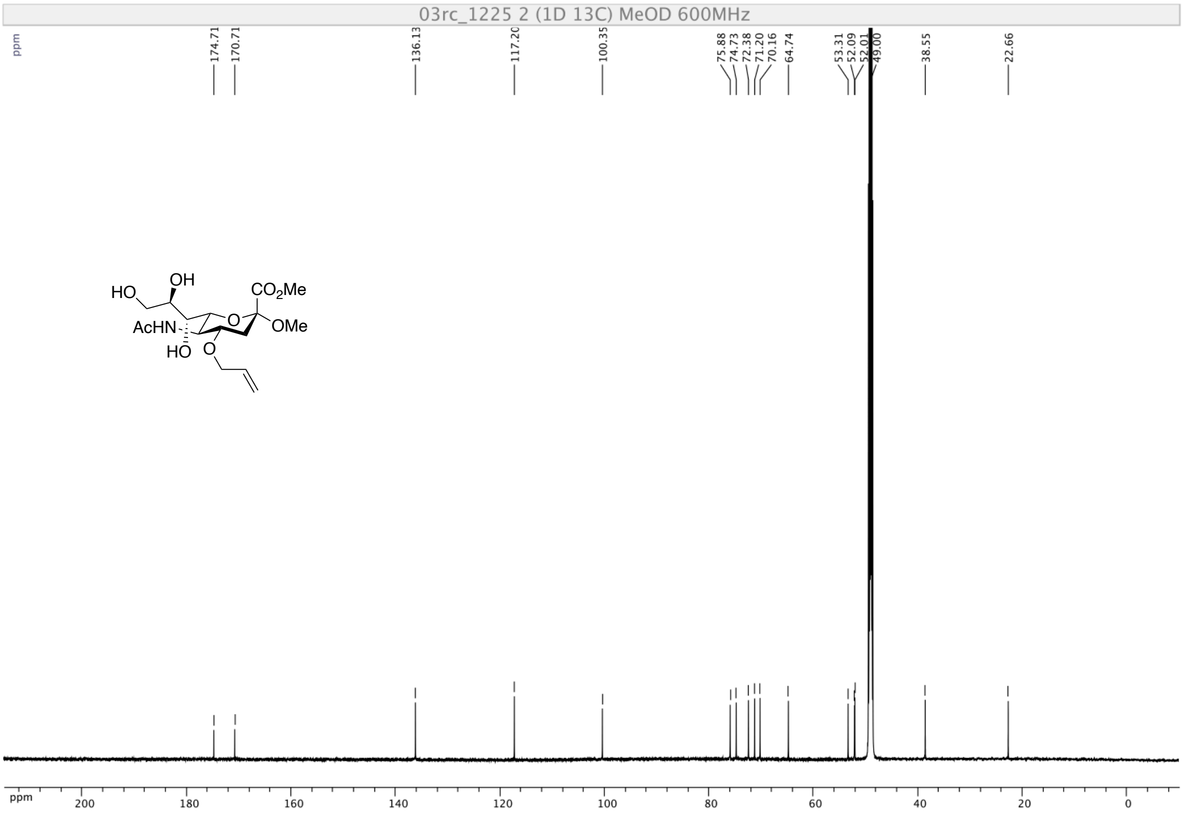
03rc_1225 2 (1D 13C) MeOD 600MHz
ppm
174.71 170.71 136.13 117.20 100.35 75.88 74.73 72.38 71.20 70.16 64.74 53.31 52.09 52.01 49.00 38.55 22.66
HO
OH
CO2Me
O
OMe
AcHN
HO
O
ppm 200 180 160 140 120 100 80 60 40 20 0

Compound **32**

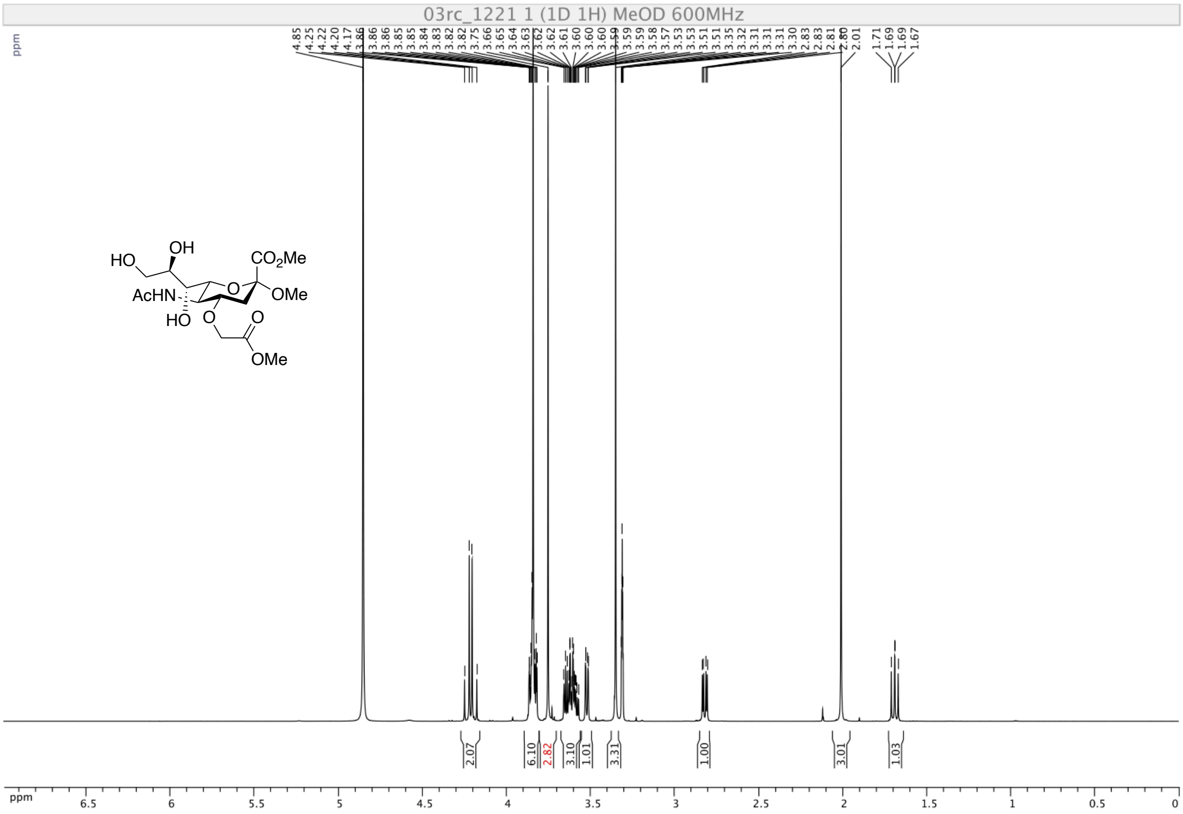
03rc_1221 1 (1D 1H) MeOD 600MHz
HO
OH
CO2Me
AcHN
OMe
HO
O
OMe

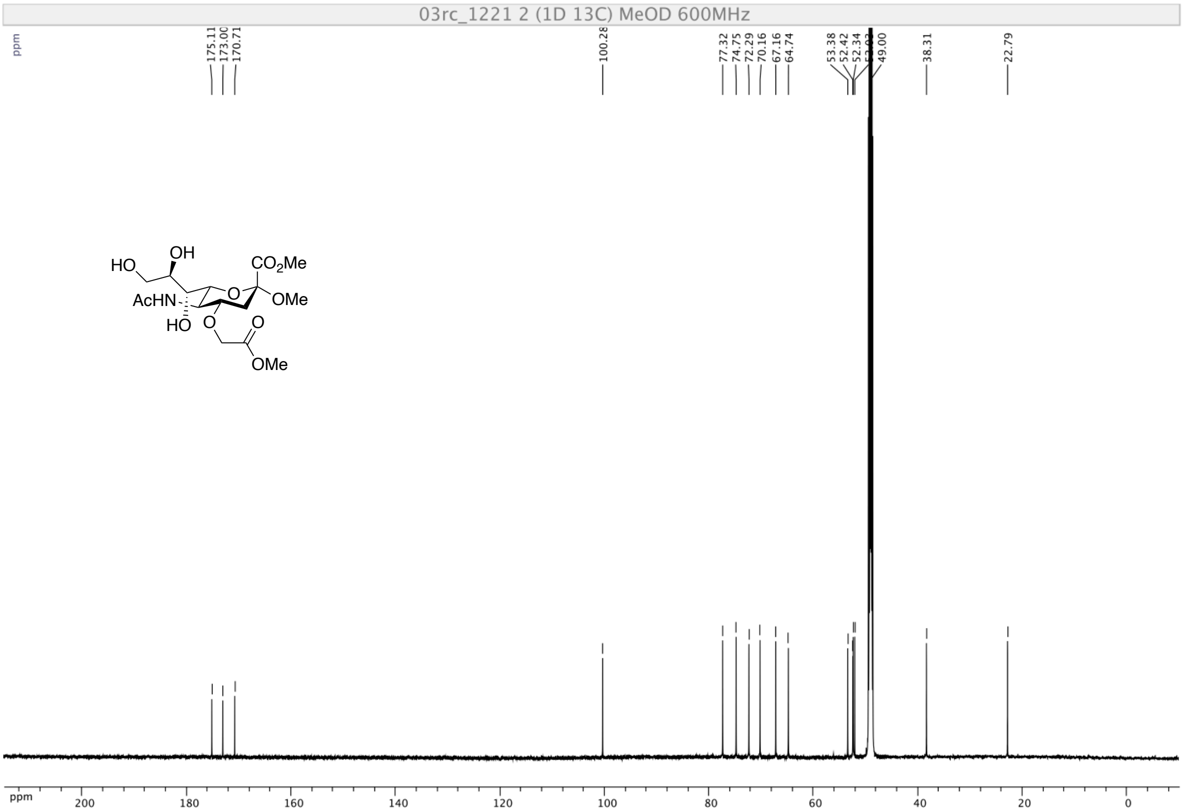
03rc_1221 2 (1D 13C) MeOD 600MHz
175.11
173.00
170.71
100.28
77.32
74.75
72.29
70.16
67.16
64.74
53.38
52.42
52.34
49.00
38.31
22.79
HO
OH
CO2Me
AcHN
OMe
HO
O
OMe

Compound **33**

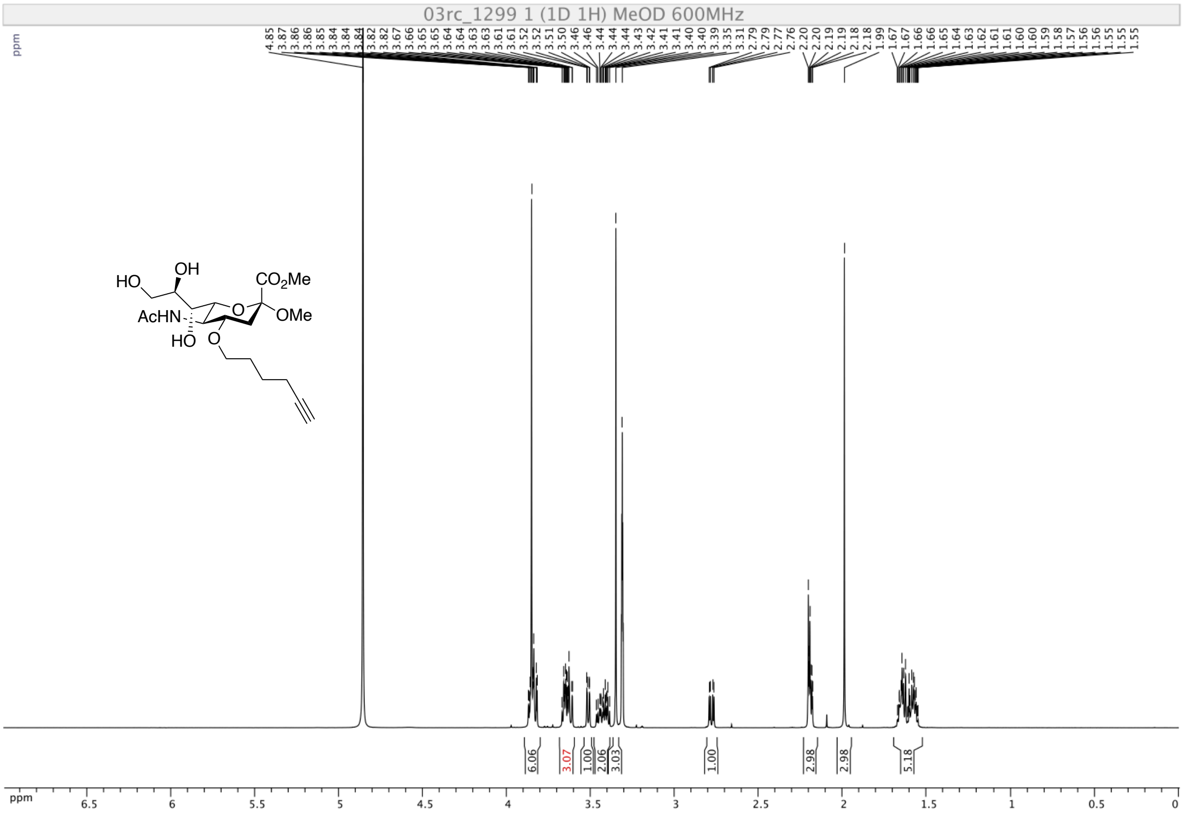
03rc_1299 1 (1D 1H) MeOD 600MHz
4.85 3.87 3.86 3.86 3.85 3.84 3.84 3.84 3.82 3.82 3.67 3.66 3.65 3.65 3.64 3.64 3.63 3.63 3.61 3.61 3.52 3.52 3.51 3.50 3.46 3.46 3.44 3.44 3.44 3.43 3.42 3.41 3.41 3.40 3.40 3.39 3.35 3.31 2.79 2.79 2.77 2.76 2.20 2.20 2.19 2.19 2.18 2.18 1.99 1.67 1.67 1.66 1.65 1.64 1.63 1.62 1.61 1.61 1.60 1.60 1.59 1.58 1.57 1.56 1.56 1.55 1.55 1.55
HO
OH
CO2Me
AcHN
O
OMe
HO
O
6.06
3.07
1.00
2.06
3.03
1.00
2.98
2.98
5.18
ppm
6.5
6
5.5
5
4.5
4
3.5
3
2.5
2
1.5
1
0.5
0

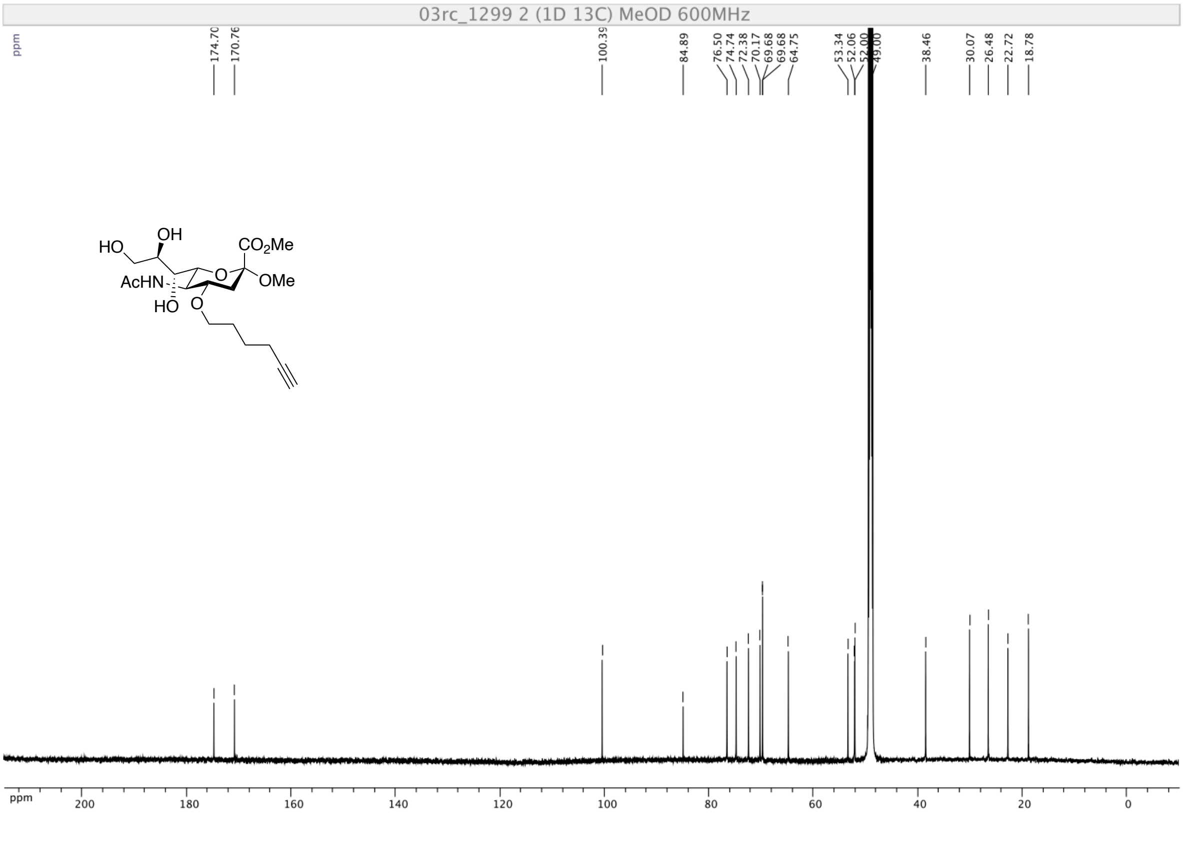
03rc_1299 2 (1D 13C) MeOD 600MHz
174.70 170.76 100.39 84.89 76.50 74.74 72.38 70.17 69.68 69.68 64.75 53.34 52.06 52.00 49.00 38.46 30.07 26.48 22.72 18.78
HO
OH
CO2Me
AcHN
O
OMe
HO
O
ppm
200
180
160
140
120
100
80
60
40
20
0

Compound **34**

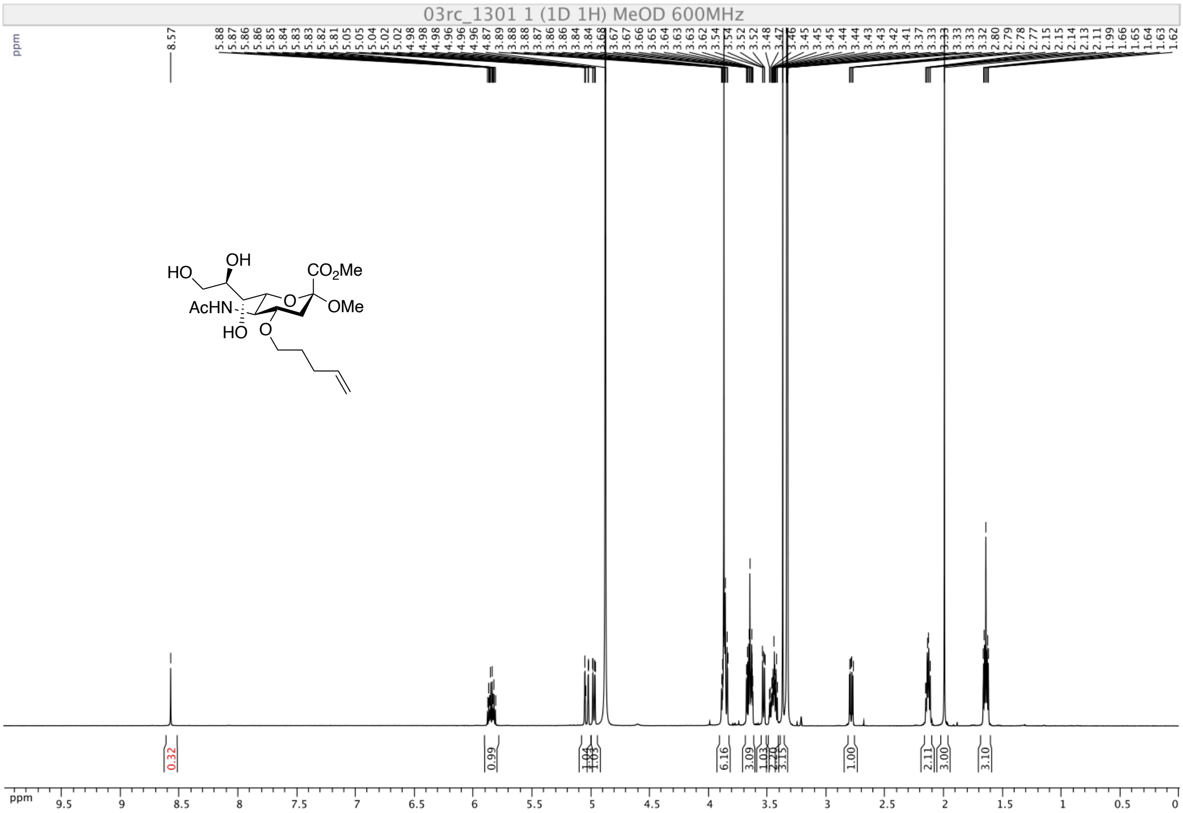
03rc_1301 1 (1D 1H) MeOD 600MHz
8.57
5.88 5.87 5.86 5.86 5.85 5.84 5.83 5.83 5.82 5.81 5.05 5.05 5.04 5.02 5.02 4.98 4.98 4.98 4.96 4.96 4.96 4.87 3.89 3.88 3.88 3.87 3.86 3.86 3.84 3.68 3.67 3.67 3.66 3.65 3.64 3.63 3.63 3.62 3.54 3.54 3.52 3.52 3.48 3.47 3.46 3.45 3.45 3.45 3.44 3.44 3.43 3.43 3.42 3.41 3.37 3.33 3.33 3.33 3.32 2.80 2.79 2.78 2.77 2.15 2.15 2.14 2.13 2.11 1.99 1.66 1.65 1.64 1.63 1.62
HO
OH
CO2Me
O
OMe
AcHN
HO
O
0.32
0.99
1.04
1.03
6.16
3.09
1.03
2.70
3.15
1.00
2.11
3.00
3.10

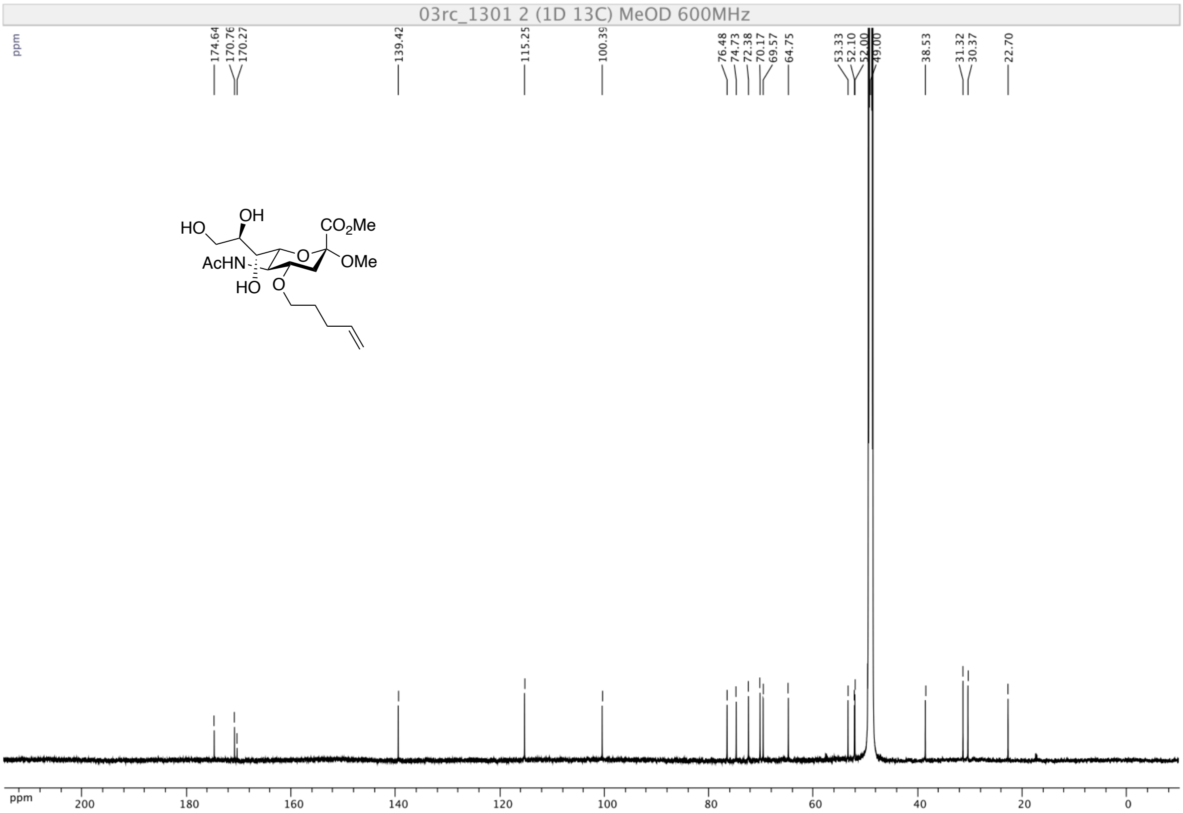
03rc_1301 2 (1D 13C) MeOD 600MHz
174.64
170.76
170.27
139.42
115.25
100.39
76.48
74.73
72.38
70.17
69.57
64.75
53.33
52.10
52.00
49.00
38.53
31.32
30.37
22.70
HO
OH
CO2Me
O
OMe
AcHN
HO
O

Compound **35**

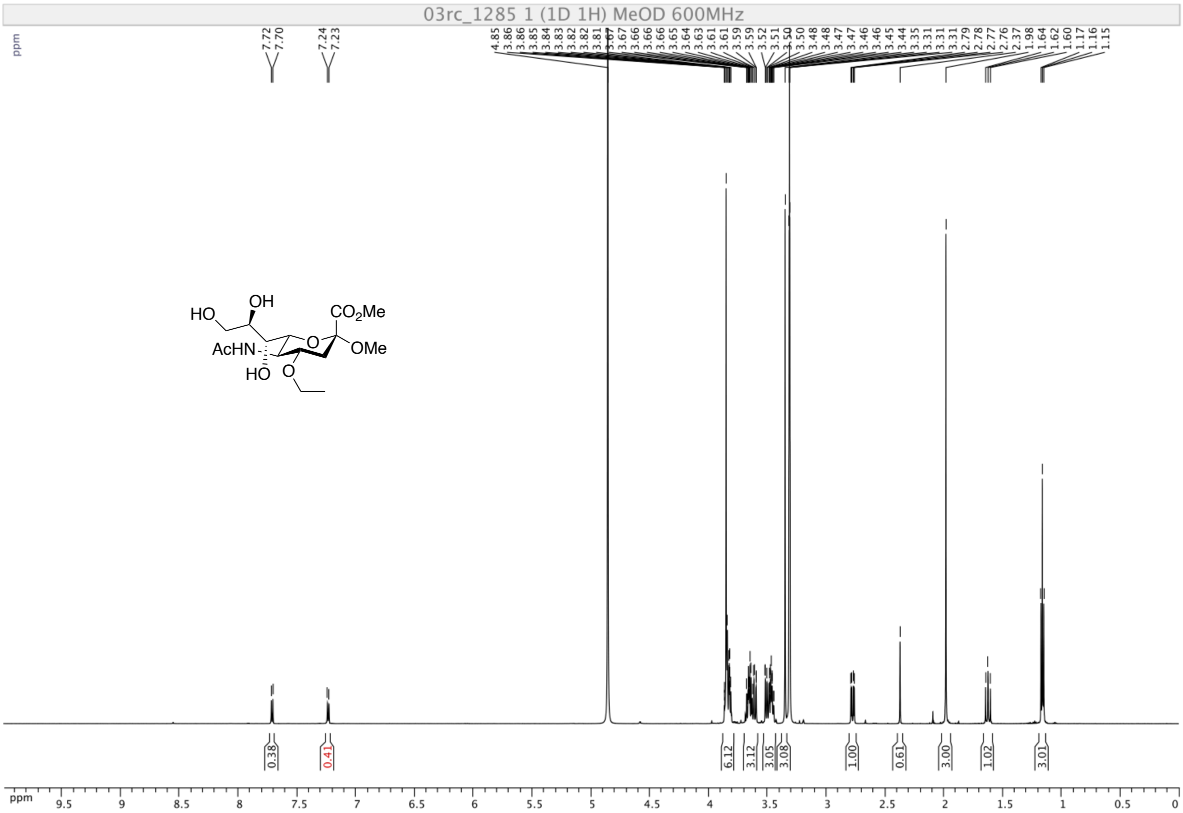
03rc_1285 1 (1D 1H) MeOD 600MHz
ppm
7.72
7.70
7.24
7.23
4.85 3.86 3.86 3.85 3.84 3.83 3.82 3.82 3.81 3.67 3.67 3.66 3.66 3.66 3.65 3.64 3.63 3.61 3.61 3.59 3.59 3.52 3.51 3.50 3.50 3.48 3.48 3.47 3.46 3.46 3.45 3.44 3.35 3.31 3.31 3.31 2.79 2.78 2.77 2.76 2.37 1.98 1.64 1.62 1.60 1.17 1.16 1.15
HO
OH
CO2Me
O
AcHN
OMe
HO
O
0.38
0.41
6.12
3.12
3.05
3.08
1.00
0.61
3.00
1.02
3.01
ppm 9.5 9 8.5 8 7.5 7 6.5 6 5.5 5 4.5 4 3.5 3 2.5 2 1.5 1 0.5 0

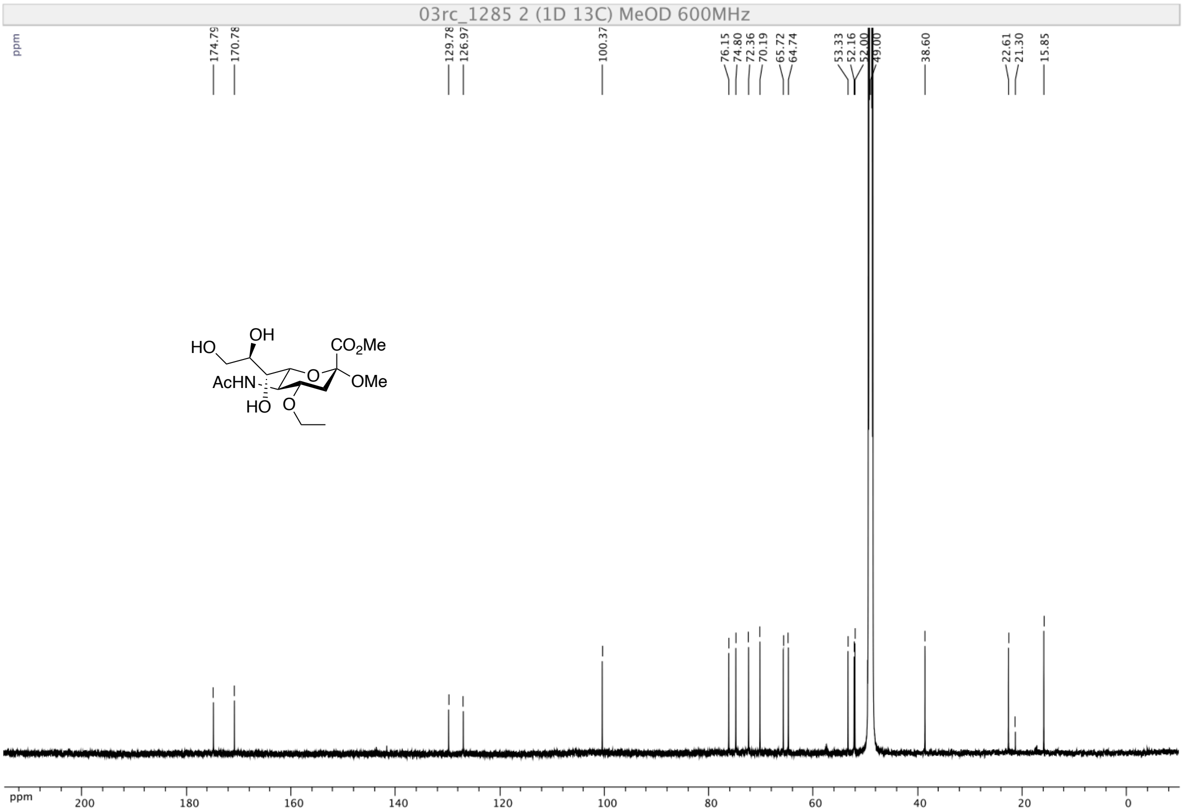
03rc_1285 2 (1D 13C) MeOD 600MHz
ppm
174.79
170.78
129.78
126.97
100.37
76.15
74.80
72.36
70.19
65.72
64.74
53.33
52.16
52.00
49.00
38.60
22.61
21.30
15.85
HO
OH
CO2Me
O
AcHN
OMe
HO
O
ppm 200 180 160 140 120 100 80 60 40 20 0

Compound **37**

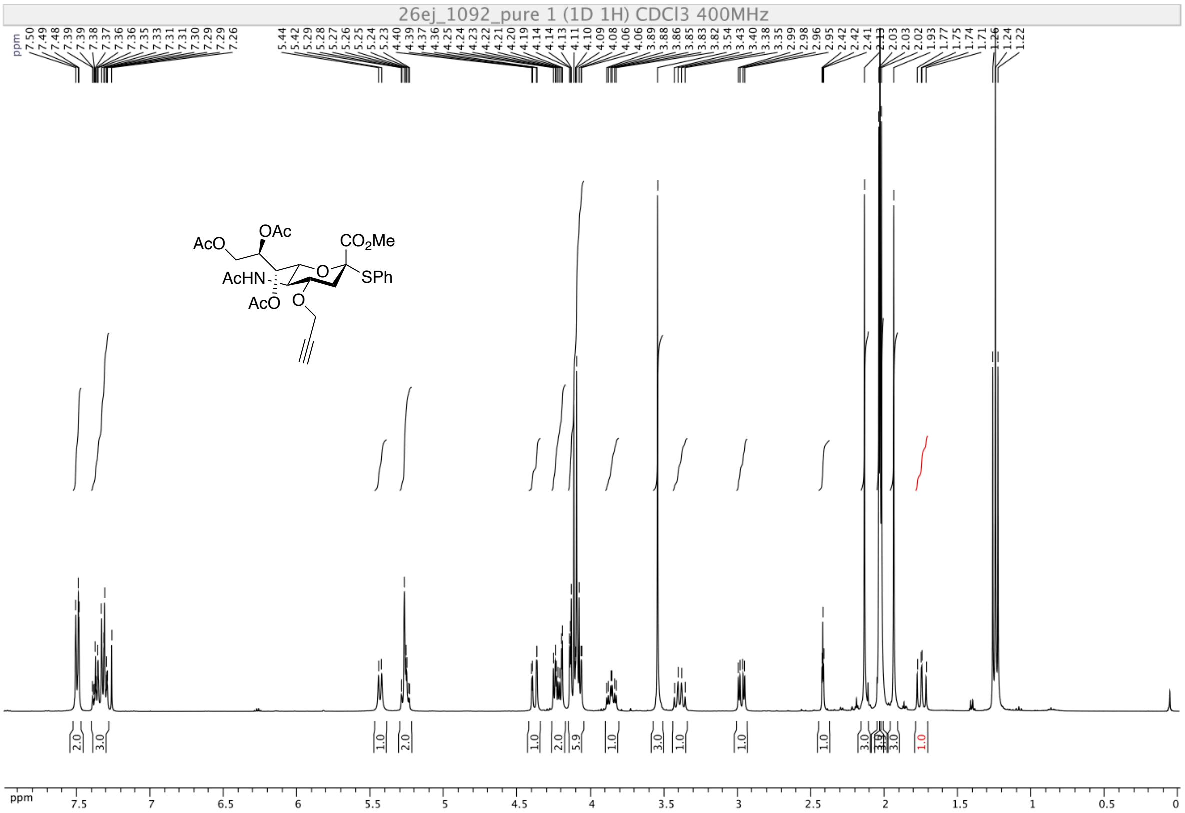
26ej_1092_pure 1 (1D 1H) CDCl3 400MHz
ppm
7.50 7.49 7.48 7.39 7.39 7.38 7.37 7.36 7.35 7.33 7.31 7.31 7.30 7.29 7.29 7.26 5.44 5.42 5.29 5.28 5.27 5.26 5.25 5.24 5.23 4.40 4.39 4.37 4.36 4.25 4.24 4.23 4.22 4.21 4.20 4.19 4.14 4.13 4.11 4.10 4.09 4.08 4.06 4.06 3.89 3.88 3.86 3.85 3.83 3.82 3.54 3.43 3.40 3.38 3.35 2.99 2.98 2.96 2.95 2.42 2.42 2.41 2.13 2.03 2.03 2.02 1.93 1.77 1.75 1.74 1.71 1.26 1.24 1.22
AcO
OAc
CO2Me
AcHN
O
SPh
AcO
O
2.0 3.0 1.0 2.0 1.0 2.0 5.9 1.0 3.0 1.0 1.0 1.0 3.0 3.0 3.0 1.0
ppm 7.5 7 6.5 6 5.5 5 4.5 4 3.5 3 2.5 2 1.5 1 0.5 0

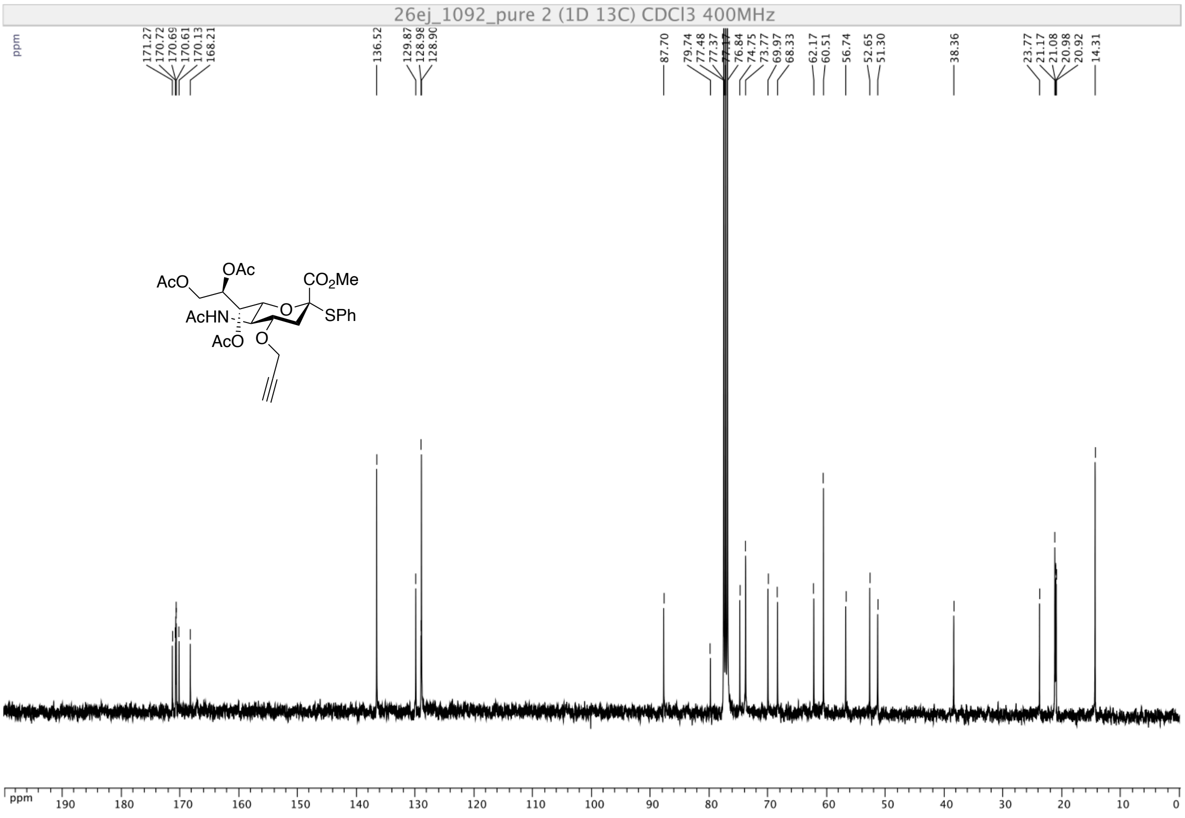
26ej_1092_pure 2 (1D 13C) CDCl3 400MHz
ppm
171.27 170.72 170.69 170.61 170.13 168.21 136.52 129.87 128.98 128.90 87.70 79.74 77.48 77.37 76.84 74.75 73.77 69.97 68.33 62.17 60.51 56.74 52.65 51.30 38.36 23.77 21.17 21.08 20.98 20.92 14.31
AcO
OAc
CO2Me
AcHN
O
SPh
AcO
O
ppm 190 180 170 160 150 140 130 120 110 100 90 80 70 60 50 40 30 20 10 0

Compound **38**

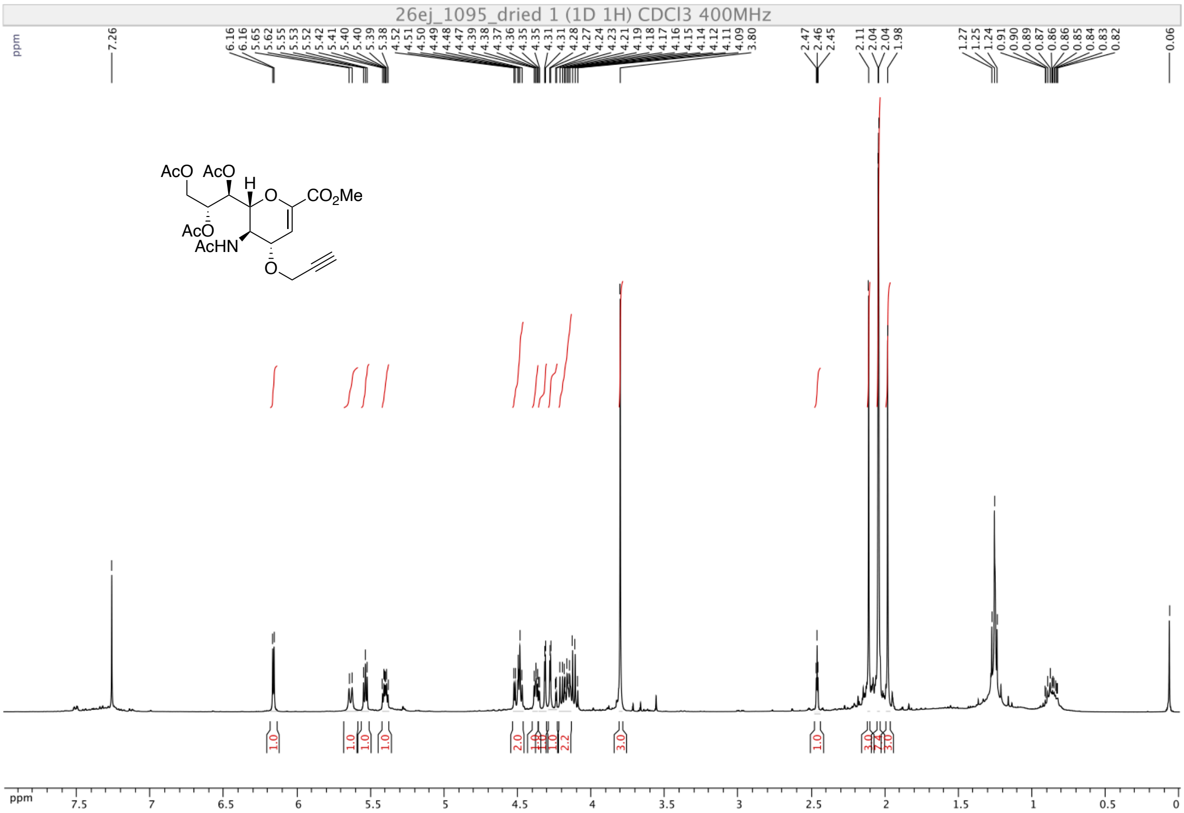
26ej_1095_dried 1 (1D 1H) CDCl3 400MHz
AcO AcO
H
O
CO2Me
AcO
AcHN
O
ppm

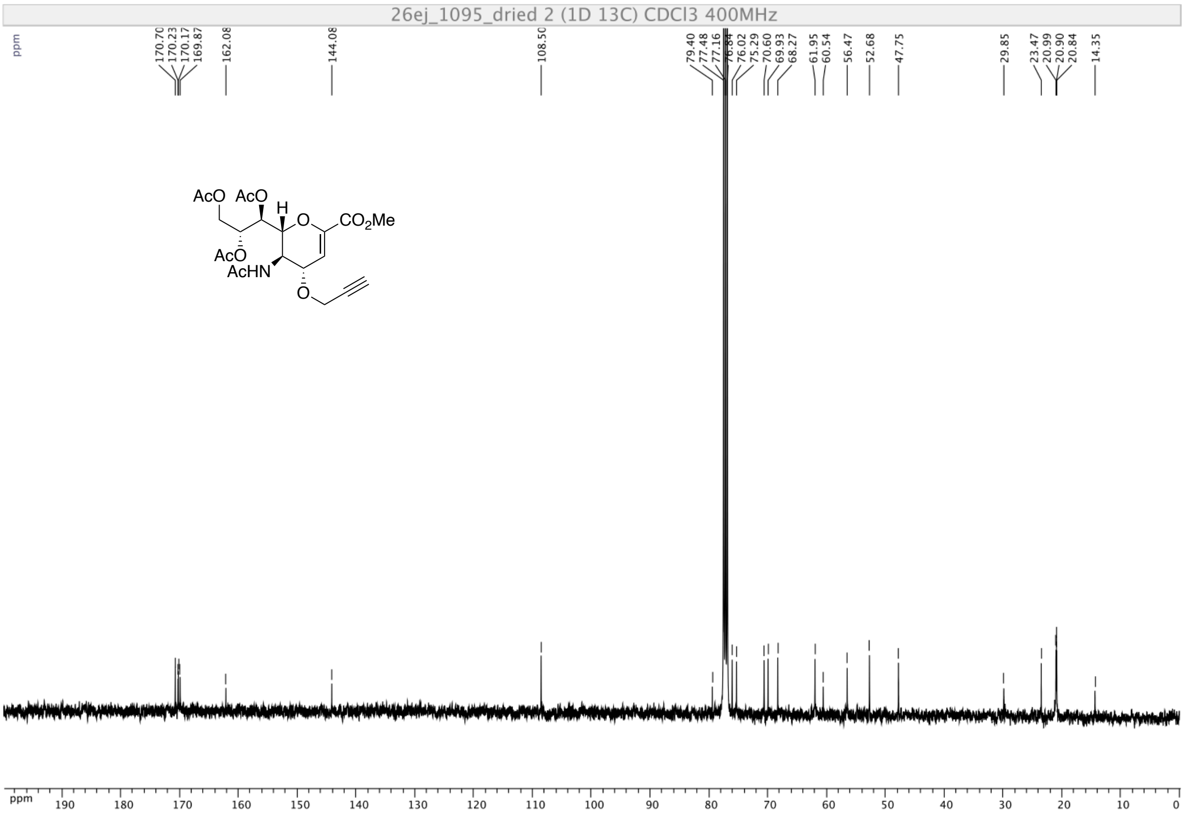
26ej_1095_dried 2 (1D 13C) CDCl3 400MHz
170.70
170.23
170.17
169.87
162.08
144.08
108.50
79.40
77.48
77.16
76.84
76.02
75.29
70.60
69.93
68.27
61.95
60.54
56.47
52.68
47.75
29.85
23.47
20.99
20.90
20.84
14.35
AcO AcO
H
O
CO2Me
AcO
AcHN
O
ppm
