# Supplementary figures and images for: Synthesis of 4-O-Alkylated N-Acetylneuraminic Acid Derivatives
Source: J Org Chem. 2021 Jun 17;86(13):9145–54. doi: 10.1021/acs.joc.1c00235 (PMC8279483; doi:10.1021/acs.joc.1c00235)

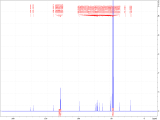

Supplement: Supplementary file 2 — jo1c00235_si_002.zip [file jo1c00235_si_002.zip › FID for publication/C30/13C/pdata/1/thumb.png]

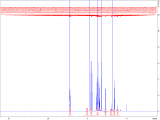

Supplement: Supplementary file 2 — jo1c00235_si_002.zip [file jo1c00235_si_002.zip › FID for publication/C30/1H/pdata/1/thumb.png]

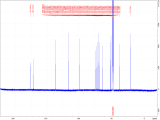

Supplement: Supplementary file 2 — jo1c00235_si_002.zip [file jo1c00235_si_002.zip › FID for publication/C31/13C/pdata/1/thumb.png]
