# Supplementary material for: Synthesis of 4-O-Alkylated N-Acetylneuraminic Acid Derivatives
Source: J Org Chem. 2021 Jun 17;86(13):9145–54. doi: 10.1021/acs.joc.1c00235 (PMC8279483; doi:10.1021/acs.joc.1c00235)
Supplement: Supplementary file 2 — jo1c00235_si_002.zip [file jo1c00235_si_002.zip › FID for publication/NMR Degradation THF/1H 60 min THF/pdata/1/email_03rc_1245_5_1.pdf]

03rc_1245, starting material + NaH 1.1 eq, 2h

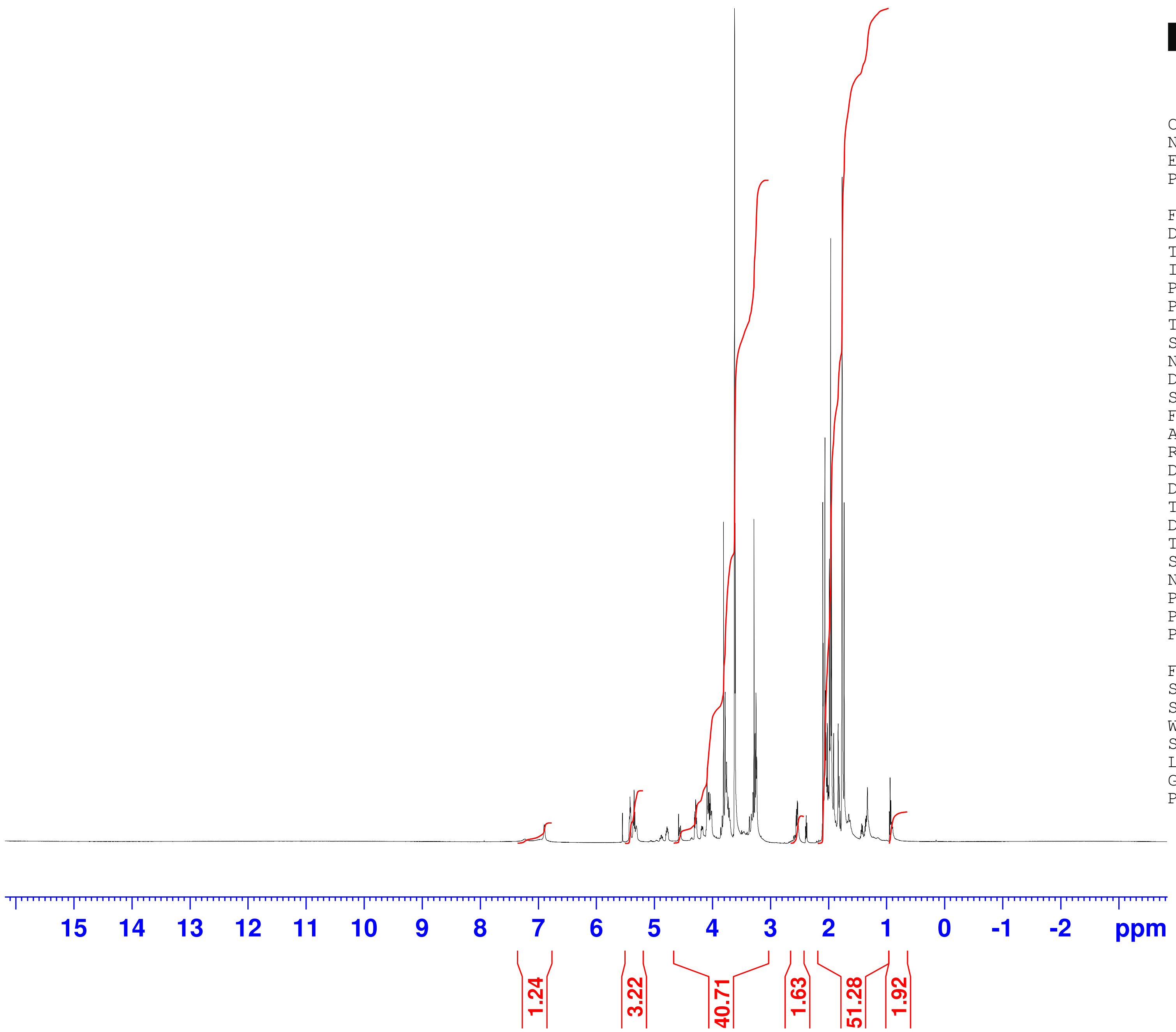


BRUKER

Current Data Parameters

| | |
|---|---|
| NAME | 03rc_1245 |
| EXPNO | 5 |
| PROCNO | 1 |

F2 - Acquisition Parameters

| | |
|---|---|
| Date_ | 20200512 |
| Time | 12.00 h |
| INSTRUM | spect |
| PROBHD | Z132572_0007 ( |
| PULPROG | zg30 |
| TD | 65536 |
| SOLVENT | THF |
| NS | 8 |
| DS | 2 |
| SWH | 12019.230 Hz |
| FIDRES | 0.366798 Hz |
| AQ | 2.7262976 sec |
| RG | 132.85 |
| DW | 41.600 usec |
| DE | 40.00 usec |
| TE | 298.0 K |
| D1 | 1.00000000 sec |
| TD0 | 1 |
| SFO1 | 600.1737063 MHz |
| NUC1 | 1H |
| P0 | 4.17 usec |
| P1 | 12.50 usec |
| PLW1 | 21.00000000 W |

F2 - Processing parameters

| | |
|---|---|
| SI | 65536 |
| SF | 600.1700000 MHz |
| WDW | EM |
| SSB | 0 |
| LB | 0.30 Hz |
| GB | 0 |
| PC | 1.00 |
