# Supplementary material for: Synthesis of 4-O-Alkylated N-Acetylneuraminic Acid Derivatives
Source: J Org Chem. 2021 Jun 17;86(13):9145–54. doi: 10.1021/acs.joc.1c00235 (PMC8279483; doi:10.1021/acs.joc.1c00235)
Supplement: Supplementary file 2 — jo1c00235_si_002.zip [file jo1c00235_si_002.zip › FID for publication/C7/1H/pdata/1/email_03rc_1171_1_1.pdf]

03rc_1171, after column

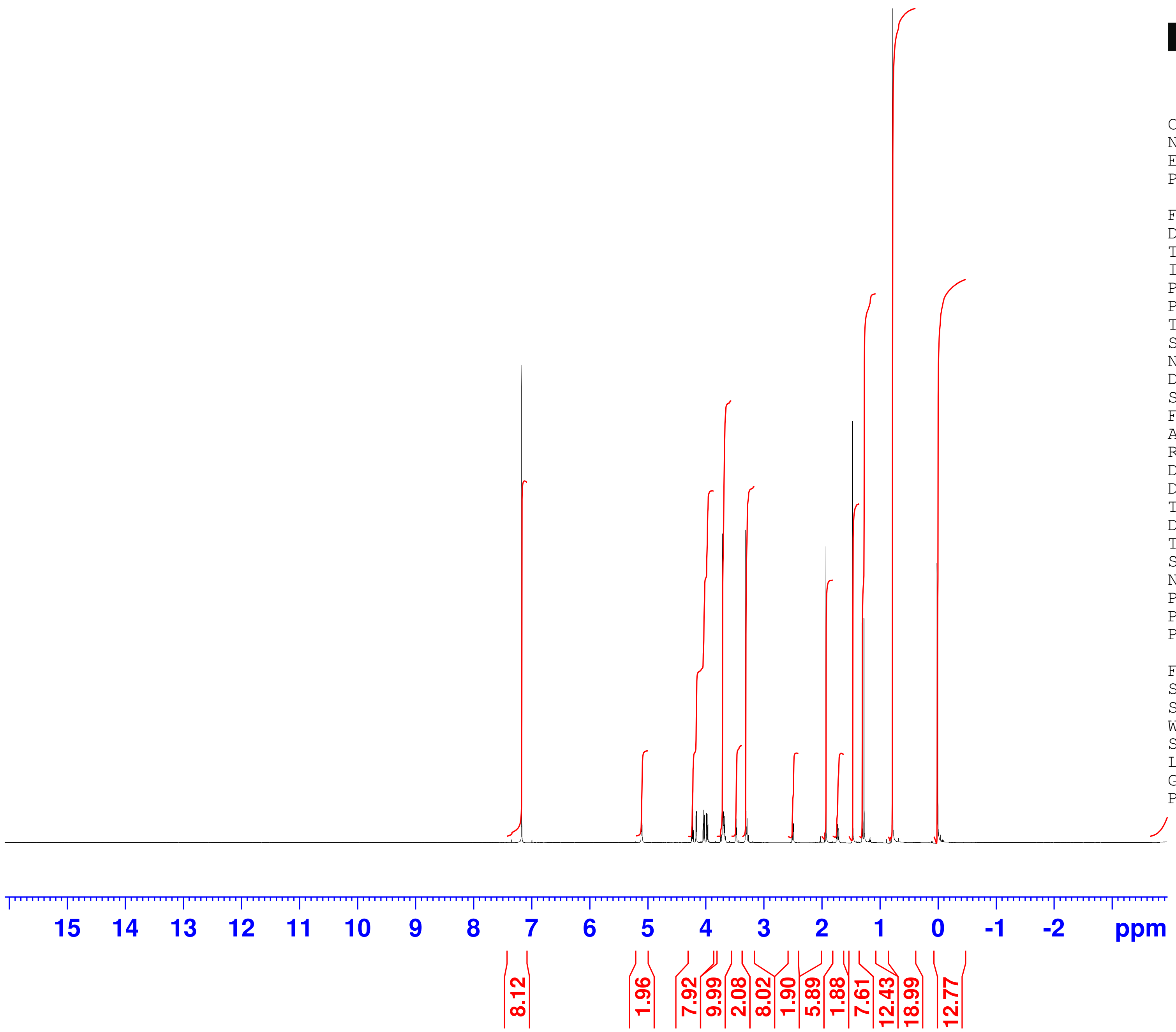

BRUKER

Current Data Parameters	
NAME	03rc_1171
EXPNO	1
PROCNO	1
F2 - Acquisition Parameters	
Date_	20200130
Time	15.34 h
INSTRUM	spect
PROBHD	Z132572_0007 (
PULPROG	zg30
TD	65536
SOLVENT	CDCl3
NS	8
DS	2
SWH	12019.230 Hz
FIDRES	0.366798 Hz
AQ	2.7262976 sec
RG	182.66
DW	41.600 usec
DE	40.00 usec
TE	298.0 K
D1	1.00000000 sec
TD0	1
SFO1	600.1737063 MHz
NUC1	1H
P0	4.15 usec
P1	12.46 usec
PLW1	21.00000000 W
F2 - Processing parameters	
SI	65536
SF	600.1700682 MHz
WDW	EM
SSB	0
LB	0.30 Hz
GB	0
PC	1.00
