# Supplementary material for: Synthesis of 4-O-Alkylated N-Acetylneuraminic Acid Derivatives
Source: J Org Chem. 2021 Jun 17;86(13):9145–54. doi: 10.1021/acs.joc.1c00235 (PMC8279483; doi:10.1021/acs.joc.1c00235)
Supplement: Supplementary file 2 — jo1c00235_si_002.zip [file jo1c00235_si_002.zip › FID for publication/C24/13C/pdata/1/email_03rc_1293_2_1.pdf]

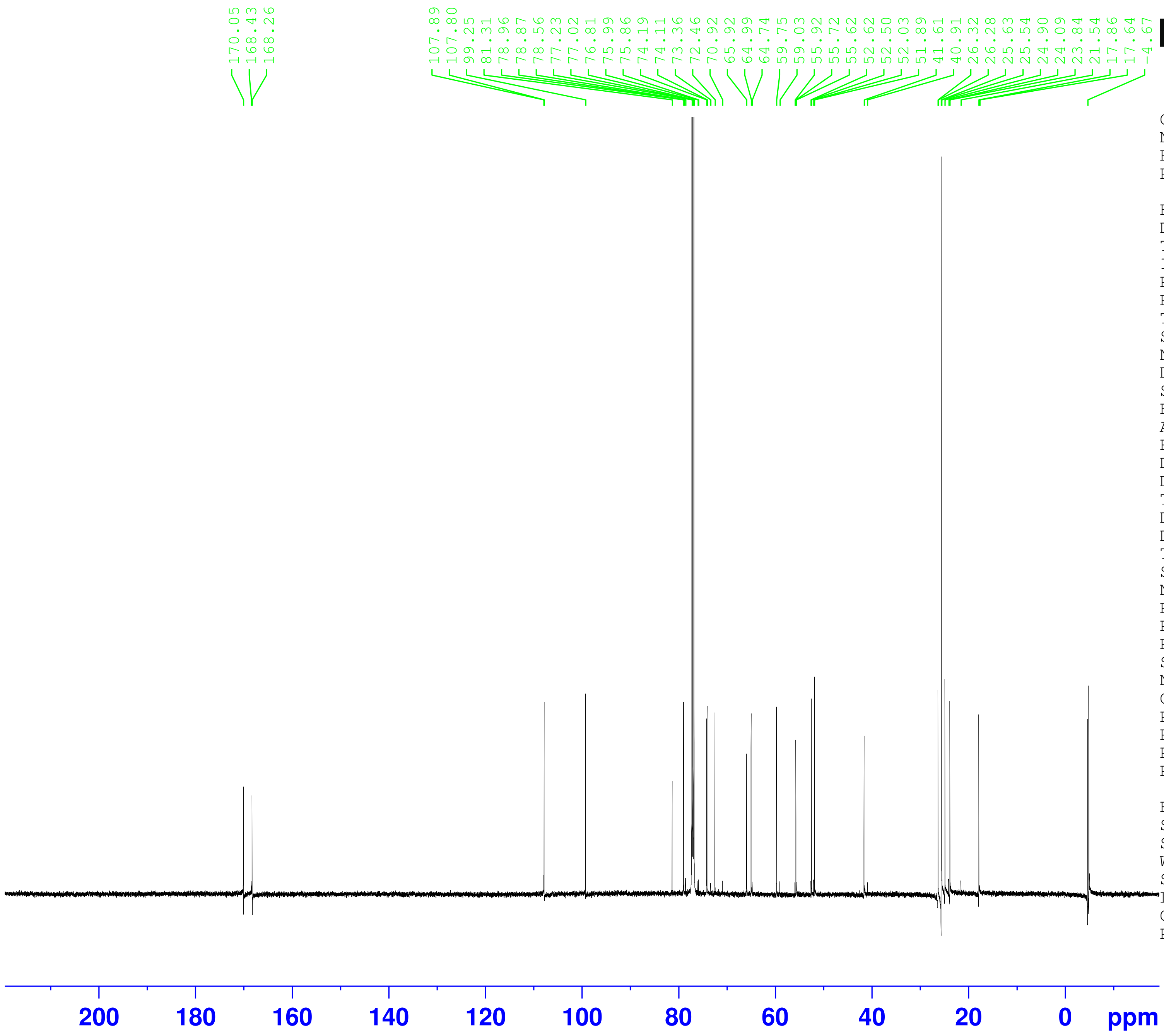

BRUKER

Current Data Parameters		
NAME	03rc_1293	
EXPNO	2	
PROCNO	1	
F2 - Acquisition Parameters		
Date_	20201017	
Time	6.13	h
INSTRUM	spect	
PROBHD	Z132572_0007 (	
PULPROG	zgpg30	
TD	65536	
SOLVENT	CDCl3	
NS	256	
DS	4	
SWH	36057.691	Hz
FIDRES	1.100393	Hz
AQ	0.9087659	sec
RG	182.66	
DW	13.867	usec
DE	18.00	usec
TE	298.0	K
D1	2.00000000	sec
D11	0.03000000	sec
TD0	1	
SFO1	150.9279571	MHz
NUC1	13C	
P0	3.23	usec
P1	9.70	usec
PLW1	35.09999847	W
SFO2	600.1724007	MHz
NUC2	1H	
CPDPRG[2	waltz16	
PCPD2	70.00	usec
PLW2	21.00000000	W
PLW12	0.59069002	W
PLW13	0.28944001	W
F2 - Processing parameters		
SI	65536	
SF	150.9128670	MHz
WDW	EM	
SSB	0	
LB	1.00	Hz
GB	0	
PC	1.40	
