# Supplementary material for: Synthesis of 4-O-Alkylated N-Acetylneuraminic Acid Derivatives
Source: J Org Chem. 2021 Jun 17;86(13):9145–54. doi: 10.1021/acs.joc.1c00235 (PMC8279483; doi:10.1021/acs.joc.1c00235)
Supplement: Supplementary file 2 — jo1c00235_si_002.zip [file jo1c00235_si_002.zip › FID for publication/C6/13C/pdata/1/email_03rc_1239_2_1.pdf]

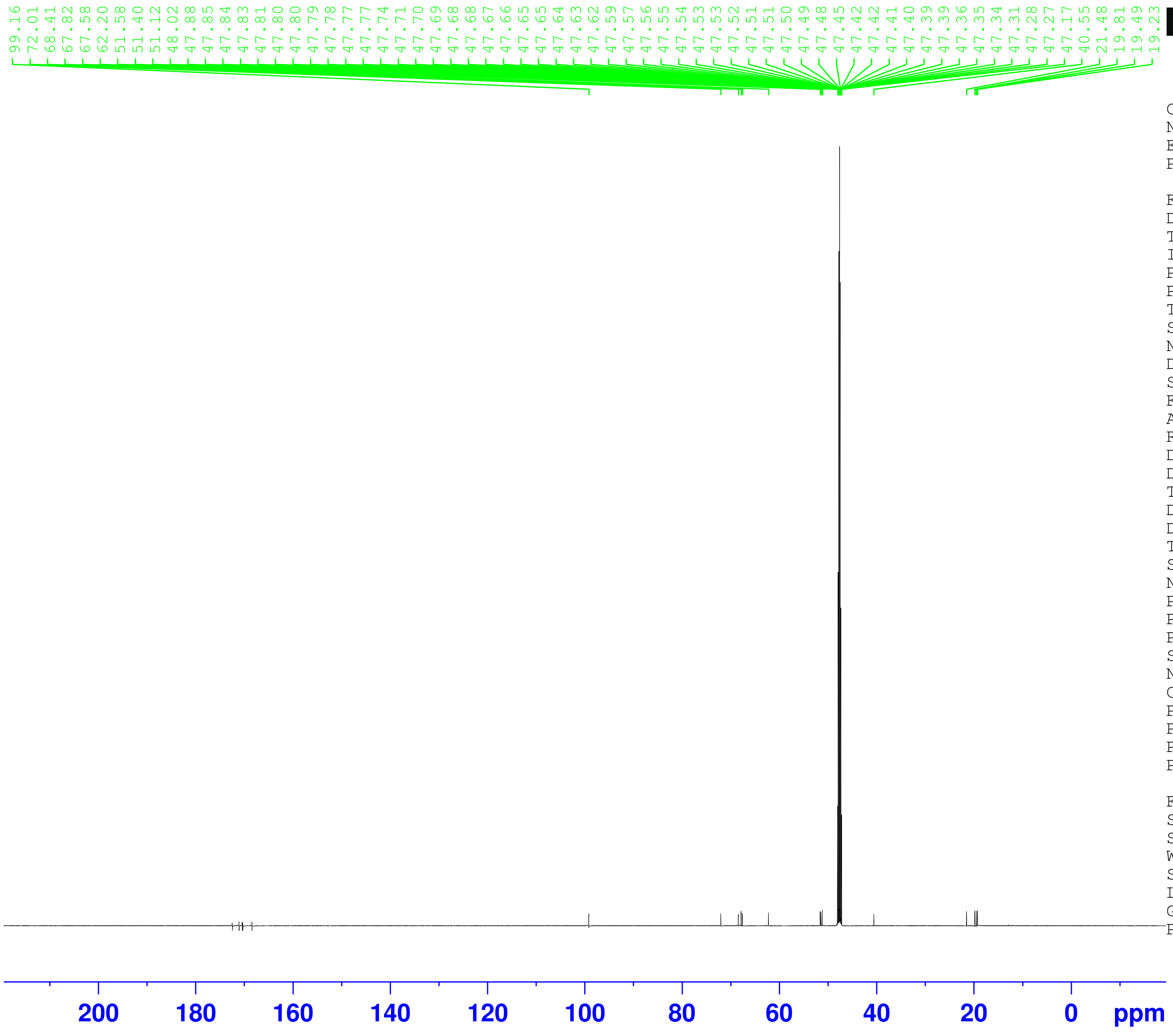

Current Data Parameters
NAME 03rc_1239
EXPNO 2
PROCNO 1

F2 - Acquisition Parameters
Date_ 20201017
Time 5.29 h
INSTRUM spect
PROBHD Z132572_0007 (
PULPROG zgpg30
TD 65536
SOLVENT MeOD
NS 256
DS 4
SWH 36057.691 Hz
FIDRES 1.100393 Hz
AQ 0.9087659 sec
RG 182.66
DW 13.867 usec
DE 18.00 usec
TE 298.0 K
D1 2.00000000 sec
D11 0.03000000 sec
TD0 1
SFO1 150.9279571 MHz
NUC1 13C
P0 3.23 usec
P1 9.70 usec
PLW1 35.09999847 W
SFO2 600.1724007 MHz
NUC2 1H
CPDPRG[2 waltz16
PCPD2 70.00 usec
PLW2 21.00000000 W
PLW12 0.59069002 W
PLW13 0.28944001 W

F2 - Processing parameters
SI 65536
SF 150.9128670 MHz
WDW EM
SSB 0
LB 1.00 Hz
GB 0
PC 1.40
