# Supplementary material for: Synthesis of 4-O-Alkylated N-Acetylneuraminic Acid Derivatives
Source: J Org Chem. 2021 Jun 17;86(13):9145–54. doi: 10.1021/acs.joc.1c00235 (PMC8279483; doi:10.1021/acs.joc.1c00235)
Supplement: Supplementary file 2 — jo1c00235_si_002.zip [file jo1c00235_si_002.zip › FID for publication/C35/1H/pdata/1/email_03rc_1285_1_1.pdf]

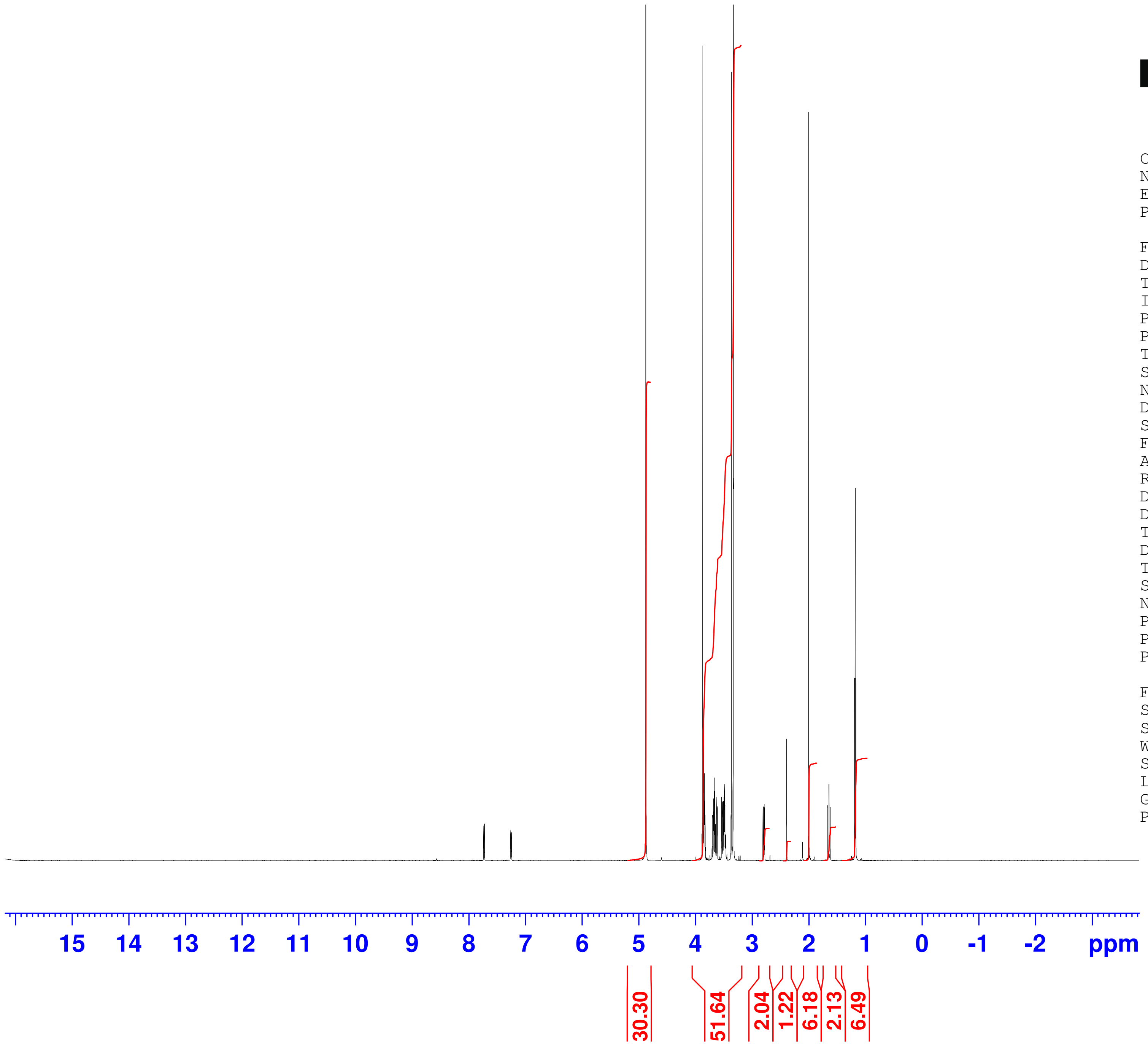


```
Current Data Parameters
NAME             03rc_1285
EXPNO                    1
PROCNO                   1

F2 - Acquisition Parameters
Date_             20201018
Time                  4.13 h
INSTRUM              spect
PROBHD   Z132572_0007 (
PULPROG               zg30
TD                   65536
SOLVENT               MeOD
NS                      32
DS                       2
SWH              12019.230 Hz
FIDRES            0.366798 Hz
AQ               2.7262976 sec
RG                  182.66
DW                  41.600 usec
DE                   40.00 usec
TE                   298.0 K
D1              1.00000000 sec
TD0                      1
SFO1           600.1737063 MHz
NUC1                    1H
P0                    4.16 usec
P1                   12.47 usec
PLW1           21.00000000 W

F2 - Processing parameters
SI                   65536
SF             600.1700000 MHz
WDW                     EM
SSB                      0
LB                    0.30 Hz
GB                       0
PC                    1.00
```
