# Supplementary material for: Synthesis of 4-O-Alkylated N-Acetylneuraminic Acid Derivatives
Source: J Org Chem. 2021 Jun 17;86(13):9145–54. doi: 10.1021/acs.joc.1c00235 (PMC8279483; doi:10.1021/acs.joc.1c00235)
Supplement: Supplementary file 2 — jo1c00235_si_002.zip [file jo1c00235_si_002.zip › FID for publication/C27/1H/pdata/1/email_03rc_1269_1_1.pdf]

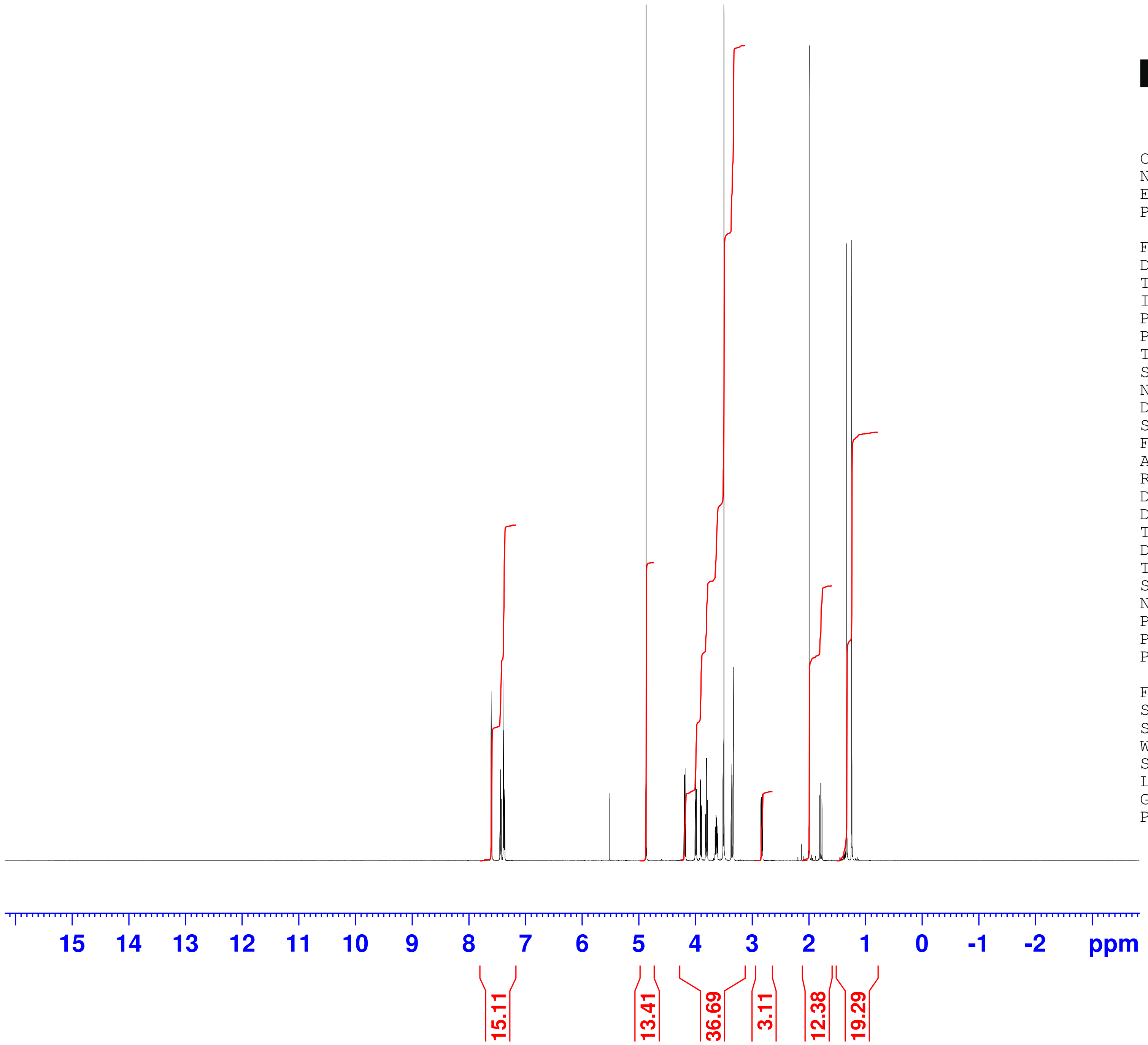

Current Data Parameters

NAME	03rc_1269
EXPNO	1
PROCNO	1

F2 - Acquisition Parameters

Date_	20201018	
Time	7.06	h
INSTRUM	spect	
PROBHD	Z132572_0007 (	
PULPROG	zg30	
TD	65536	
SOLVENT	MeOD	
NS	16	
DS	2	
SWH	12019.230	Hz
FIDRES	0.366798	Hz
AQ	2.7262976	sec
RG	145.45	
DW	41.600	usec
DE	40.00	usec
TE	298.0	K
D1	1.00000000	sec
TD0	1	
SFO1	600.1737063	MHz
NUC1	1H	
P0	4.12	usec
P1	12.36	usec
PLW1	21.00000000	W

F2 - Processing parameters

SI	65536	
SF	600.1700000	MHz
WDW	EM	
SSB	0	
LB	0.30	Hz
GB	0	
PC	1.00	
