# Supplementary material for: Synthesis of 4-O-Alkylated N-Acetylneuraminic Acid Derivatives
Source: J Org Chem. 2021 Jun 17;86(13):9145–54. doi: 10.1021/acs.joc.1c00235 (PMC8279483; doi:10.1021/acs.joc.1c00235)
Supplement: Supplementary file 2 — jo1c00235_si_002.zip [file jo1c00235_si_002.zip › FID for publication/C29/13C/pdata/1/email_03rc_1313-12min_2_1.pdf]

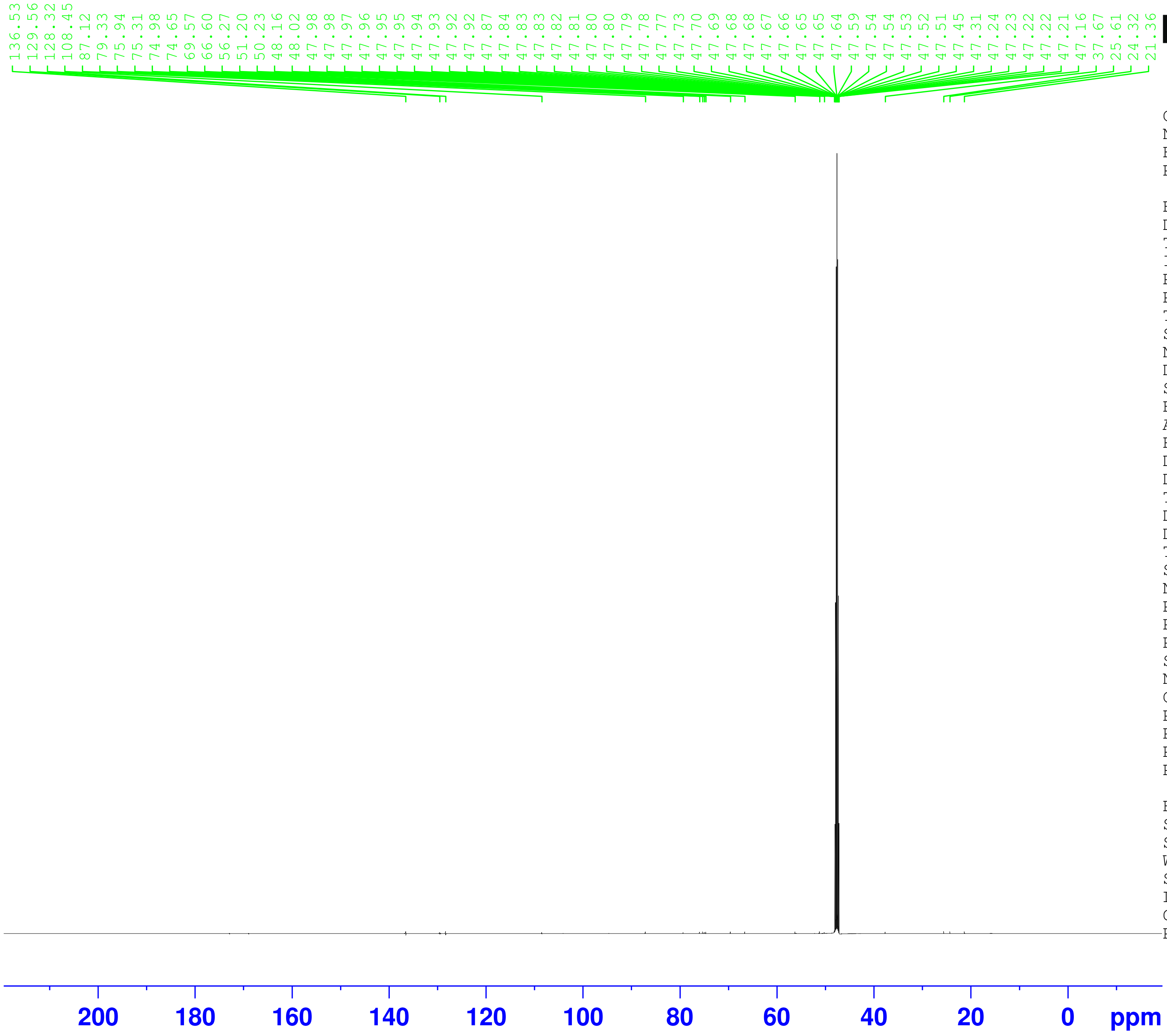

BRUKER

Current Data Parameters

NAME	03rc_1313-12min
EXPNO	2
PROCNO	1

F2 - Acquisition Parameters

Date_	20201017
Time	5.09 h
INSTRUM	spect
PROBHD	Z132572_0007 (
PULPROG	zgpg30
TD	65536
SOLVENT	MeOD
NS	3072
DS	4
SWH	36057.691 Hz
FIDRES	1.100393 Hz
AQ	0.9087659 sec
RG	182.66
DW	13.867 usec
DE	18.00 usec
TE	298.0 K
D1	2.00000000 sec
D11	0.03000000 sec
TD0	1
SFO1	150.9279571 MHz
NUC1	13C
P0	3.23 usec
P1	9.70 usec
PLW1	35.09999847 W
SFO2	600.1724007 MHz
NUC2	1H
CPDPRG[2	waltz16
PCPD2	70.00 usec
PLW2	21.00000000 W
PLW12	0.59069002 W
PLW13	0.28944001 W

F2 - Processing parameters

SI	65536
SF	150.9128670 MHz
WDW	EM
SSB	0
LB	1.00 Hz
GB	0
PC	1.40
