# Supplementary material for: Synthesis of 4-O-Alkylated N-Acetylneuraminic Acid Derivatives
Source: J Org Chem. 2021 Jun 17;86(13):9145–54. doi: 10.1021/acs.joc.1c00235 (PMC8279483; doi:10.1021/acs.joc.1c00235)
Supplement: Supplementary file 2 — jo1c00235_si_002.zip [file jo1c00235_si_002.zip › FID for publication/C29/1H/pdata/1/email_03rc_1313-12min_1_1.pdf]

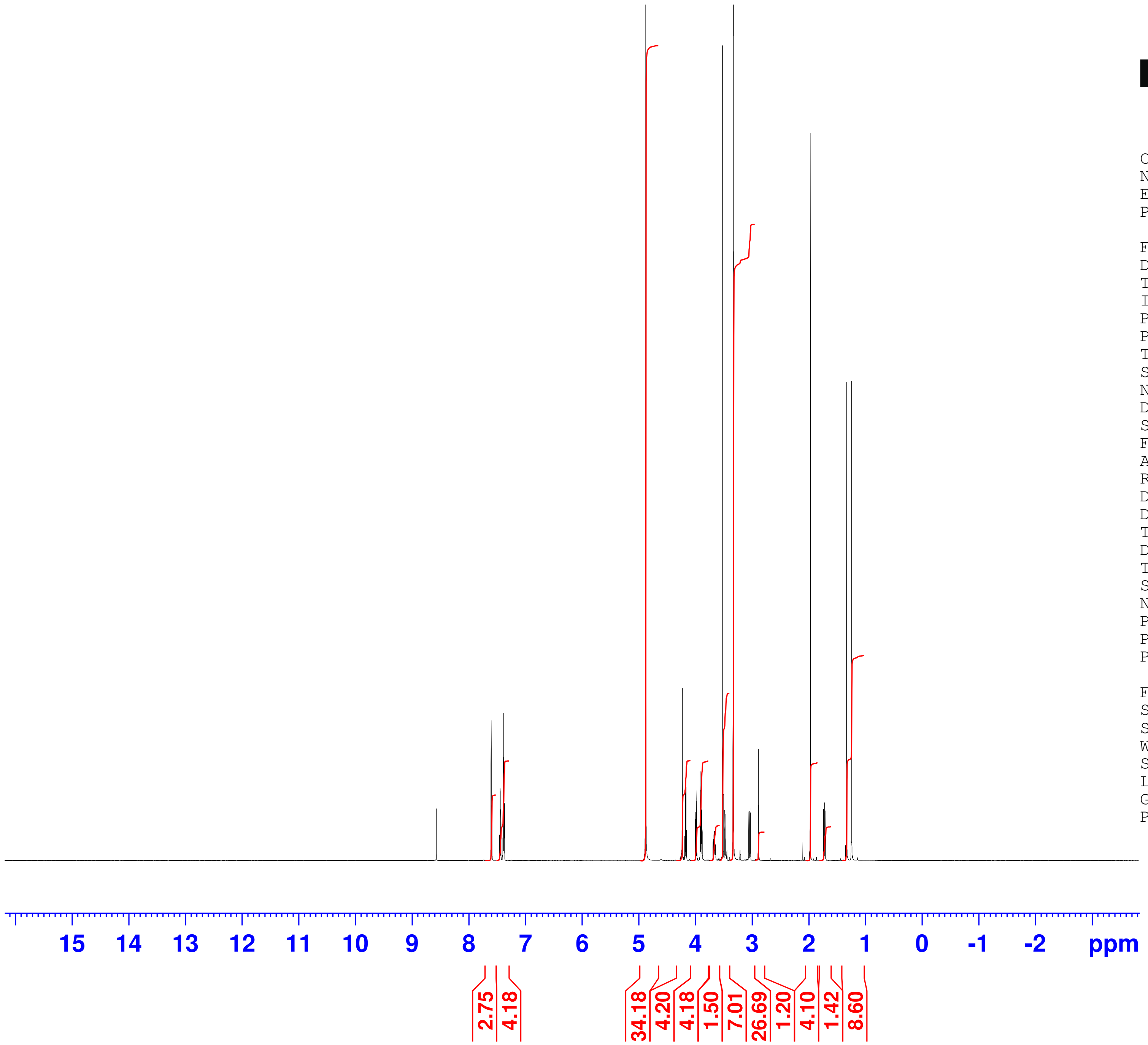


```
Current Data Parameters
NAME      03rc_1313-12min
EXPNO                  1
PROCNO                 1

F2 - Acquisition Parameters
Date_           20201017
Time                2.35 h
INSTRUM            spect
PROBHD   Z132572_0007 (
PULPROG             zg30
TD                 65536
SOLVENT             MeOD
NS                    64
DS                     2
SWH            12019.230 Hz
FIDRES          0.366798 Hz
AQ             2.7262976 sec
RG                182.66
DW                41.600 usec
DE                 40.00 usec
TE                 298.0 K
D1            1.00000000 sec
TD0                    1
SFO1         600.1737063 MHz
NUC1                  1H
P0                  4.12 usec
P1                 12.36 usec
PLW1         21.00000000 W

F2 - Processing parameters
SI                 65536
SF           600.1700000 MHz
WDW                   EM
SSB                    0
LB                  0.30 Hz
GB                     0
PC                  1.00
```
