# Supplementary material for: Synthesis of 4-O-Alkylated N-Acetylneuraminic Acid Derivatives
Source: J Org Chem. 2021 Jun 17;86(13):9145–54. doi: 10.1021/acs.joc.1c00235 (PMC8279483; doi:10.1021/acs.joc.1c00235)
Supplement: Supplementary file 2 — jo1c00235_si_002.zip [file jo1c00235_si_002.zip › FID for publication/C16/1H/pdata/1/email_03rc_1227_1_1.pdf]

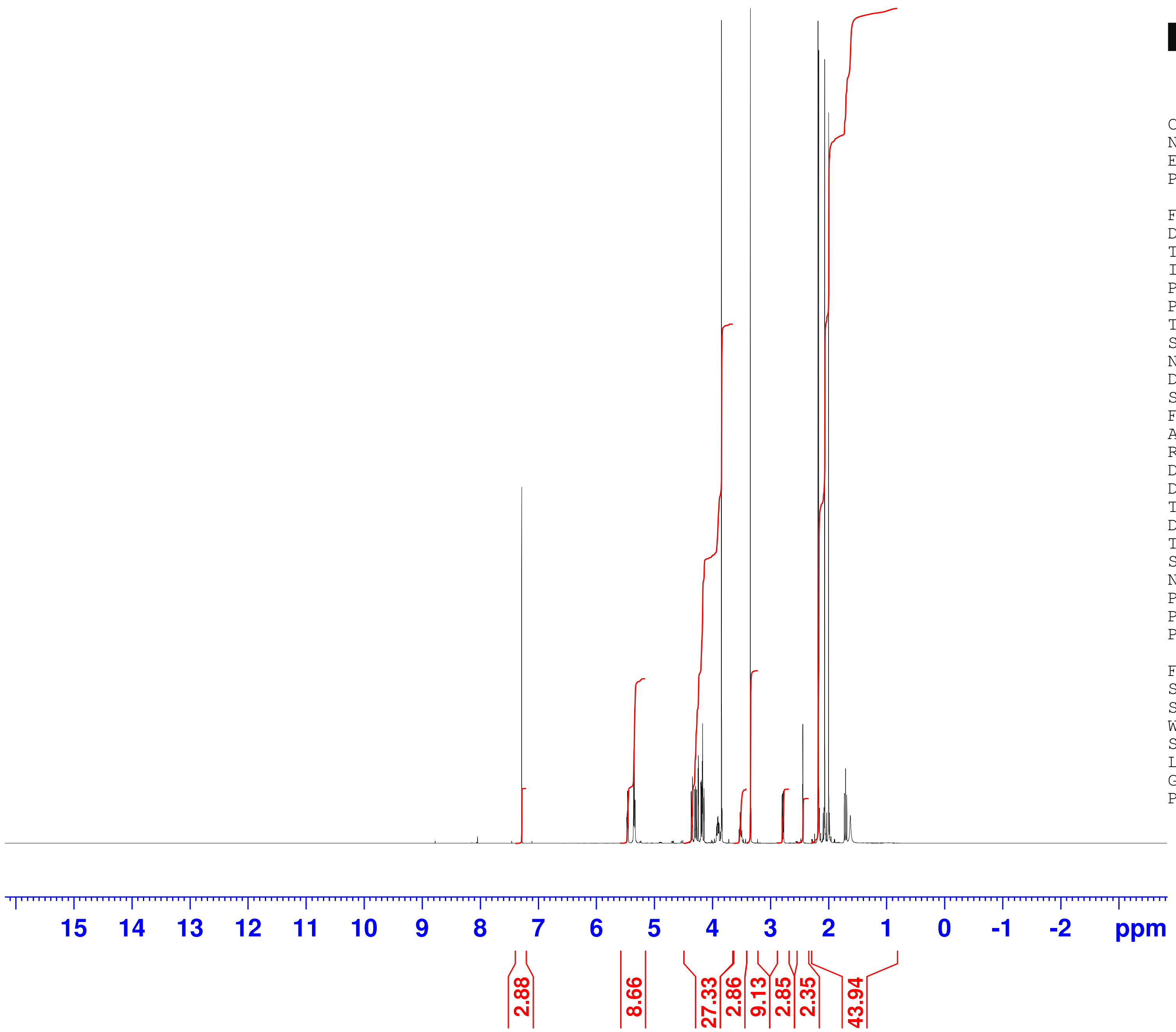


BRUKER

Current Data Parameters
NAME 03rc_1227
EXPNO 1
PROCNO 1

F2 - Acquisition Parameters
Date_ 20201017
Time 6.30 h
INSTRUM spect
PROBHD Z132572_0007 (
PULPROG zg30
TD 65536
SOLVENT CDCl3
NS 16
DS 2
SWH 12019.230 Hz
FIDRES 0.366798 Hz
AQ 2.7262976 sec
RG 182.66
DW 41.600 usec
DE 40.00 usec
TE 298.0 K
D1 1.00000000 sec
TD0 1
SFO1 600.1737063 MHz
NUC1 1H
P0 3.78 usec
P1 11.34 usec
PLW1 21.00000000 W

F2 - Processing parameters
SI 65536
SF 600.1700000 MHz
WDW EM
SSB 0
LB 0.30 Hz
GB 0
PC 1.00
