# Supplementary material for: Synthesis of 4-O-Alkylated N-Acetylneuraminic Acid Derivatives
Source: J Org Chem. 2021 Jun 17;86(13):9145–54. doi: 10.1021/acs.joc.1c00235 (PMC8279483; doi:10.1021/acs.joc.1c00235)
Supplement: Supplementary file 2 — jo1c00235_si_002.zip [file jo1c00235_si_002.zip › FID for publication/C11/1H/pdata/1/email_03rc_1237_1_1.pdf]

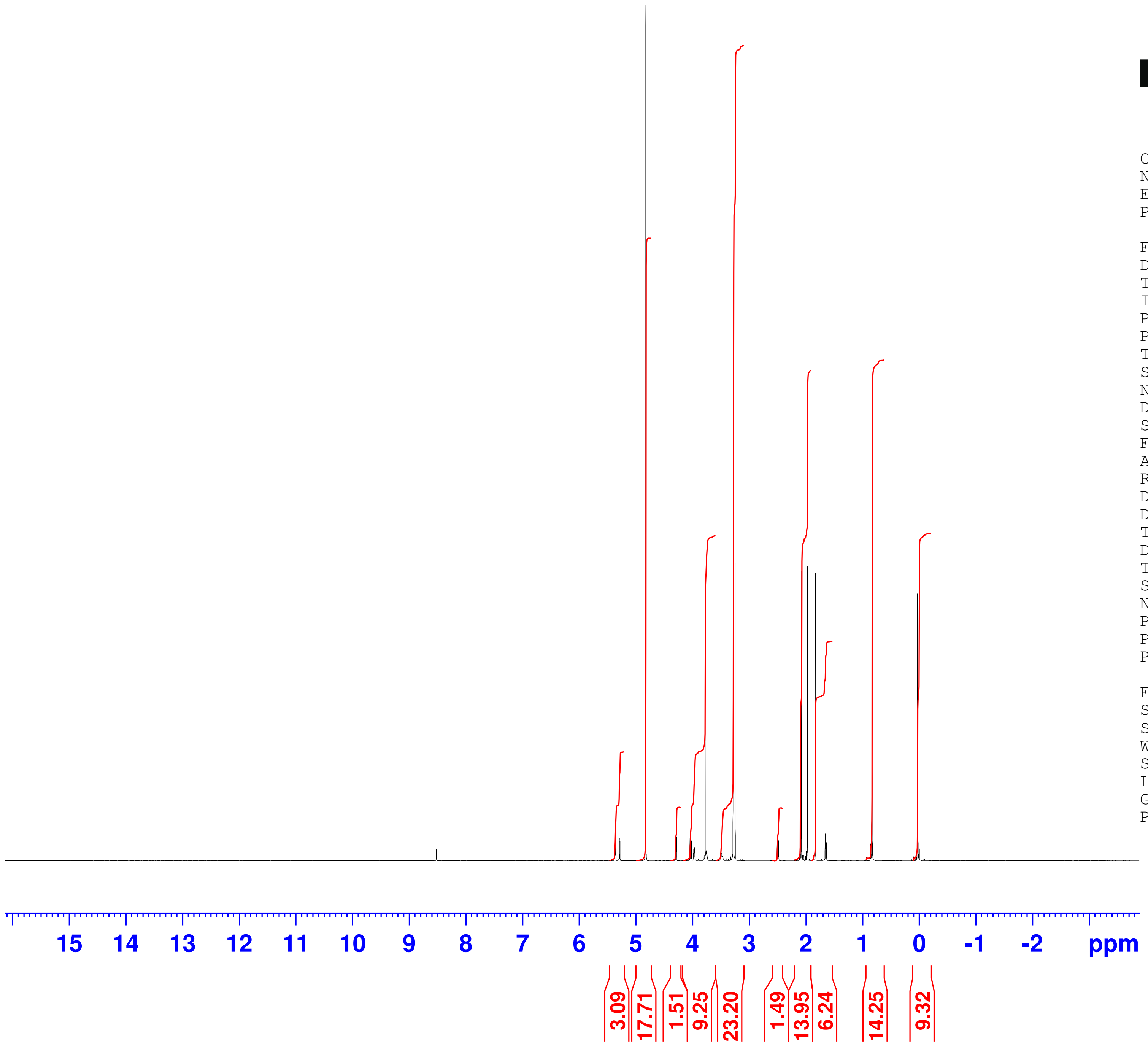

BRUKER

```
Current Data Parameters
NAME              03rc_1237
EXPNO                     1
PROCNO                    1

F2 - Acquisition Parameters
Date_              20201018
Time                   2.58 h
INSTRUM               spect
PROBHD   Z132572_0007 (
PULPROG                zg30
TD                    65536
SOLVENT                MeOD
NS                       32
DS                        2
SWH               12019.230 Hz
FIDRES             0.366798 Hz
AQ                2.7262976 sec
RG                   182.66
DW                   41.600 usec
DE                    40.00 usec
TE                    298.0 K
D1               1.00000000 sec
TD0                       1
SFO1            600.1737063 MHz
NUC1                     1H
P0                     4.16 usec
P1                    12.49 usec
PLW1            21.00000000 W

F2 - Processing parameters
SI                    65536
SF              600.1700306 MHz
WDW                      EM
SSB                       0
LB                     0.30 Hz
GB                        0
PC                     1.00
```
